# Synthesis, Fungitoxic Activity against *Botrytis cinerea* and Phytotoxicity of Alkoxyclovanols and Alkoxyisocaryolanols

**DOI:** 10.3390/jof7121079

**Published:** 2021-12-15

**Authors:** Adriana de Almeida Pinto Bracarense, Jociani Ascari, Giovanni Gontijo de Souza, Thays Silva Oliveira, Antonio Ruano-González, Ana A. Pinto, Maria Amélia Diamantino Boaventura, Jacqueline Aparecida Takahashi, Isidro G. Collado, Rosa Durán-Patrón, Antonio J. Macías-Sánchez

**Affiliations:** 1 Departamento de Química, Instituto de Ciências Exatas, Universidade Federal de Minas Gerais, Av. Presidente Antônio Carlos, 6627, Belo Horizonte 31270-901, MG, Brazil; adrianabracarense@gmail.com (A.d.A.P.B.); jascari@utfpr.edu.br (J.A.); giovannigs1@yahoo.com.br (G.G.d.S.); tsoliveira3@yahoo.com.br (T.S.O.); dianadb@netuno.lcc.ufmg.br (M.A.D.B.); jat@qui.ufmg.br (J.A.T.); 2 Departamento de Química, Centro Federal de Educação Tecnológica de Minas Gerais, Av. Amazonas, 5253, Belo Horizonte 30421-169, MG, Brazil; 3 Coordenaçao Ciências Biológicas, Universidade Tecnológica Federal do Paraná, Prolongamento da Rua Cerejeira, s/n, Santa Helena 85892-000, PR, Brazil; 4 Departamento de Química Orgánica, Facultad de Ciencias, Universidad de Cádiz, Puerto Real, 11510 Cádiz, Spain; antonio.ruano@uca.es (A.R.-G.); ana.pinto@mail.uca.es (A.A.P.); isidro.gonzalez@uca.es (I.G.C.); 5 Instituto de Investigación en Biomoléculas (INBIO), Universidad de Cádiz, Puerto Real, 11510 Cádiz, Spain

**Keywords:** *Botrytis cinerea*, antifungal, phytotoxic activity, clovane, isocaryolane, *Lactuca sativa*

## Abstract

Clovane and isocaryolane derivatives have been proven to show several levels of activity against the phytopathogenic fungus *Botrytis cinerea*. Both classes of sesquiterpenes are reminiscent of biosynthetic intermediates of botrydial, a virulence factor of *B. cinerea*. Further development of both classes of antifungal agent requires exploration of the structure–activity relationships for the antifungal effects on *B. cinerea* and phytotoxic effects on a model crop. In this paper, we report on the preparation of a series of alkoxy-clovane and -isocaryolane derivatives, some of them described here for the first time (**2b**, **2d**, **2f**–**2h**, and **4c**–**4e**); the evaluation of their antifungal properties against *B. cinerea*, and their phytotoxic activites on the germination of seeds and the growth of radicles and shoots of *Lactuca sativa* (lettuce). Both classes of compound show a correlation of antifungal activity with the nature of side chains, with the best activity against *B. cinerea* for **2d**, **2h**, **4c** and **4d**. In general terms, while 2-alkoxyclovan-9-ols (**2a**–**2e**) exert a general phytotoxic effect, this is not the case for 2-arylalkoxyclovan-9-ols (**2f**–**2i**) and 8-alkoxyisocaryolan-9-ols (**4a**–**4d**), where stimulating effects would make them suitable candidates for application to plants.

## 1. Introduction

Fungi of the *Botrytis* (Sclerotiniaceae) genus are involved in substantial crop losses in economically relevant cultivars. While most species are host specific, *B. cinerea* has a wide host range which involves hundreds of vascular plant genera [1]. Infection is usually promoted by high humidity conditions, involving a variety of pathways to achieve infection and colonization [2]. Chemical control based on synthetic fungicides [3] is still the prevalent protection option for many crops, but, their increasing impact on the environment [4], concerns on residue levels and effective delivery [5], and the ever-present acquired resistance phenomenon [6], are forcing a rethinking of the way strategies of chemical control are used [7].

On the other hand, in the context of integrated pest management [8], there is an increasing interest in the use of biopesticides [9], which comprises the use of living microorganisms and substances of natural origin, such as plant and microbial extracts, as well as natural products isolated from the abovementioned sources or closely related derivatives (selected examples can be found from plants [10,11,12], microorganisms [13,14,15,16] and endophytes [17,18,19]). Therefore, there is a need for the development of novel chemical control agents, capable of being used in conjunction with living microorganisms and with the ability to antagonize *B. cinerea*, which calls for aiming at selective targets in the fungal infection cycle. Evidence of the protective role of endophytic microorganisms against the attack of *B. cinerea* on relevant cultivars [20,21] stresses the need for chemical control agents with selectivity against the phytopathogen, as this would reduce the impact on the plant microbiome.

It has been reported that commercial fungicides have an impact on plant physiology [22]. For instance, the treatment of *Vitis vinifera* with fludioxonil and pyrimethanil, used in the control of *B. cinerea*, impairs photosynthesis [23,24], while mancozeb, an extensively used contact fungicide, induces oxidative damage on *L. sativa* [25]. Therefore, the development of bioinspired antifungal agents should be undertaken keeping in mind these undesired effects.

Botrydial is a low molecular weight phytotoxin [26], with a carbon skeleton not following the isoprene rule, which originates from farnesyl diphosphate by folding, cyclization, and a sequence of oxidative transformations [27,28,29,30]. Genes encoding for the relevant steps of the metabolic pathway to botrydial and related compounds have also been elucidated [31,32]. This secondary metabolite is part of the interaction of the fungus with the host plant, inducing cell death [33]. The modulation of its biosynthesis may lead to the control of the fungus through a selective mechanism, as symptoms of the disease can be decreased by inhibition of the production of the toxin in deleted mutants [34].

Our research group has approached the control of the fungus *B. cinerea* by developing nonphytotoxic analogs of the biosynthetic intermediates of the phytotoxin botrydial. A variety of compounds with different sesquiterpenic carbon skeletons have been prepared for this purpose [26].

**Figure 1:**
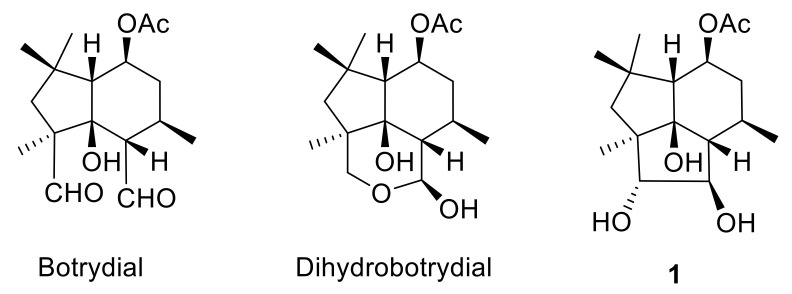
Metabolites from *Botrytis cinerea*.

Clovane and isocaryolane skeleton compounds are caryophyllene cyclization derivatives [35,36], which are reminiscent of botrydial biosynthetic intermediates such as **1** (Figure 1). Within this context, 2-alkoxyclovan-9-ols, obtained by the cyclization of caryophyllene oxide in alcohols, catalyzed by TCNE [37], have shown different levels of activity, which are correlated with the nature of the substituent at C-2 [38]. On the other hand, the metabolism of selected 2-alkoxyclovan-9-ols has shown the preference of the fungus for the dealkylation of the side chain at C-2, yielding less active compounds [39,40].

Reported antifungal activities of clovane derivatives show a general decrease of the activity when further hydroxyl groups are introduced, or when the side chain at C-2 is absent [39,40]. On the other hand, QSAR calculations suggest an increase in the fungal activity if the lipophilicity of the side chain at C-2 is increased [38]. Evaluation of the antifungal activity of a homologous series of 2-alkoxyclovan-9-ol derivatives and some systematic variations of active 2-(aryl)alkoxyclovan-9-ols described before [38], should shed some light on the extent and limitations of this lipophylic effect.

8-Methoxyisocaryolan-9-ol, an isocaryolane derivative, has also shown an interesting level of antifungal activity against the phytopathogen *B. cinerea* [41]. Incubation of this compound and related substrates like isocaryolane-8,9-diol and isocaryolan-9-ol showed, on one hand, that these compounds interfered with the production of botryane metabolites, like dihydrobotrydial (Figure 1), a metabolic sink of botryane metabolism [42]. On the other hand, it was apparent that the presence of a methoxy group at C-8 was a key feature for the antifungal activity of isocaryolane derivatives; this ether was cleaved by fungal metabolism and antifungal activity decreased as the hydroxylation level increased [41,43]. Therefore, evaluation of a homologous series of 8-alkoxyisocaryolan-9-ol derivatives should shed some light on the suitability of isocaryolane compounds as antifungal leads.

Another aspect to consider in the development of clovane or isocaryolane derivatives as effective fungal leads, is the extent of the phytotoxic effect exerted by these compounds, which should be reduced as much as possible if the compounds of interest are to find practical applications in the field.

Therefore, in this paper, we report the preparation of a homologous series of 2-alkoxyclovan-9-ols (**2a**–**2e**), some related to 2-arylethoxyclovan-9-ols (**2f**–**2i**), as well as a homologous series of 8-alkoxyisocaryolan-9-ols (**4a**–**4e**). We also evaluate their antifungal properties against *B. cinerea*, and their phytotoxic activities on the germination of seeds and growth of radicles and shoots of *L. sativa* (lettuce) [44,45,46,47], a plant successfully used in the evaluation of the phytotoxicity of bioactive natural products.

## 2. Materials and Methods

### 2.1. General Experimental Procedure

Melting points were measured with a Reichert-Jung Kofler block and are uncorrected. Optical rotations were determined on a Perkin-Elmer 341 polarimeter. IR spectra were recorded on a Perkin-Elmer Spectrum BX FT-IR spectrophotometer. ^1^H and ^13^C NMR spectra were obtained on a Varian INOVA 400 NMR spectrometer using tetramethylsilane as an internal reference. NMR assignments were made by a combination of 1D and 2D techniques and by comparison with assignments available in the literature for previously described compounds, where appropriate. Mass spectra were recorded on a Finnigan Voyager spectrometer at 70 eV. High resolution mass spectra were recorded on a Micromass Autospec spectrometer at 70 eV or on a Waters Synapt G2 QTOF spectrometer in ESI mode. HPLC was performed with a Hitachi/Merck L-6270 apparatus equipped with a UV-VIS detector (L 4250) and a differential refractometer detector (RI-71). TLC was performed on Merck Kiesegel 60 F254 plates, with a 0.2 mm thick film. Silica gel (Merck) was used for column chromatography. Purification by HPLC was performed using a Si gel column (LiChrospher Si 60, 10 μm, 1 cm wide, 25 cm long).

### 2.2. General Procedure for the Alcoholysis of Caryophyllene Oxide Catalyzed by TCNE

**Figure 2:**
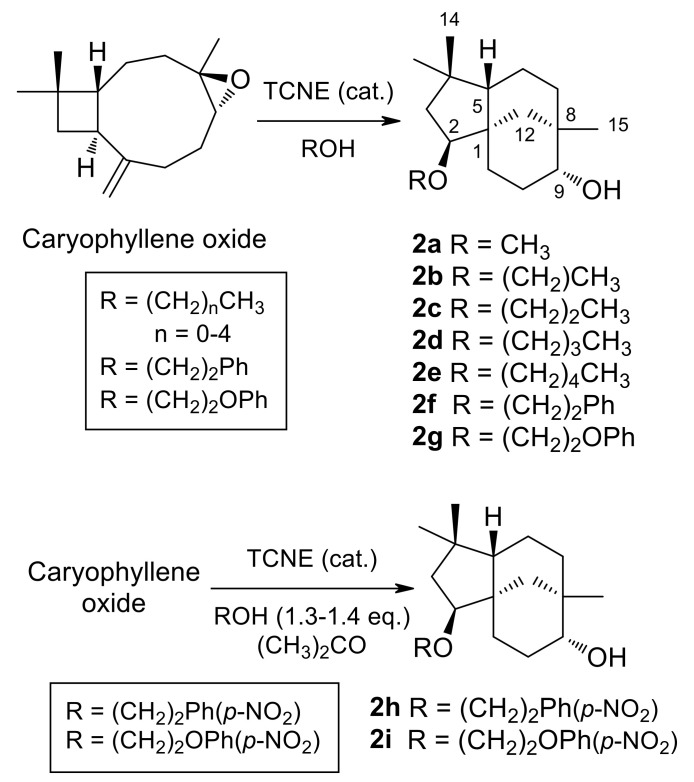
Synthesis of 2-Alkoxyclovan-9-ols (**2a**–**2e**) and 2-Arylethoxyclovan-9-ols (**2f**–**2i**).

(-)-Caryophyllene oxide, dissolved in alcohol, was treated with a catalytic amount of TCNE at room temperature (see Table 1 for details). When the epoxide was consumed, as shown by TLC monitoring, the solvent was either evaporated under vacuum or washed with brine. The resulting gum was dissolved in ethyl acetate and dried over anhydrous Na_2_SO_4_. After filtration, evaporation under reduced pressure of the solvent afforded a crude reaction product. Purification by column chromatography on silica gel, with increasing gradients of ethyl acetate in petroleum ether, combined with HPLC purification, afforded **2a**–**2g** (Figure 2).

(2*S*,9*R*)-2-Methoxyclovan-9-ol (**2a**), (2*S*,9*R*)-2-propoxyclovan-9-ol (**2c**), and (2*S*,9*R*)-2-pentoxyclovan-9-ol (**2e**) were obtained according to procedures described in the literature [37,48]. Reaction conditions, products and yields for **2b**, **2d**, and **2f**, **2g** can be found in Table 1.

#### 2.2.1. Spectroscopic Data of Compounds **2b**,**2d**,**2f**,**2g**

*(2S,9R)-2-ethoxyclovan-9-ol* (**2b**): needles (CHCl_3_); 58–60 °C; ${\left[\alpha \right]}_{D\;}^{25}\;$+ 1.5 (*c* 3.8, CDCl_3_); IR (film) *ν*_max_ 3422, 2927, 2864, 1459, 1365, 1107 cm^−1^; ^1^H NMR data (CDCl_3_, 400 MHz), see Table 2; ^13^C NMR data (CDCl_3_, 100 MHz), see Table 3; HMBC (selected correlations) C-1 → H-3α, H-3β, H-5β, H-6b, H-10α, H-11a, H-12a; C-2 → H-3α, H-3β, H-12a, H-1′a,b; C-4 → H-3α, H-3β, H-5β, H-13α, H-14β; C-8 → H-5β, H-7a, H-7b, H-10α, H-12a; C-15 → H-7a, H-12a; C-1′ → H-2α, H-2β; C-2′ → H-1′a,b; EIMS *m*/*z* 266 [M]^+^ (19), 251 (16), 220 (13), 205 (20), 192 (21), 161 (20), 151 (12), 135 (26), 113 (100), 95 (16), 85 (33); HREIMS *m*/*z* 266.2268 [M]^+^ (calcd for C_17_H_30_O_2_, 266.2246).

*(2S,9R)-2-butoxyclovan-9-ol* (**2d**): needles (CHCl_3_); 50–52 °C; ${\left[\alpha \right]}_{D\;}^{25}\;$+ 10.0 (*c* 3.8, CHCl_3_); IR (film) *ν*_max_ 3406, 2927, 2864, 1463, 1361, 1106 cm^−1^; ^1^H NMR data (CDCl_3_, 400 MHz), see Table 2; ^13^C NMR data (CDCl_3_, 100 MHz), see Table 3; HMBC (selected correlations) C-1 → H-2α, H-3α, H-3β, H-6a, H-6b, H-11a, H-12a; C-2 → H-3α, H-3β, H-12b, H-1′a,b; C-4 → H-3α, H-3β, H-13α, H-14β; C-8 → H-7a, H-12a, H-15; C-15 → H-7a, H-7b, H-12a, H-12b; C-1′ →, H-2α, H_2_-2′, H_2_-3′; C-2′ → H-1′a,b, H_2_-3′, H_3_-4′; C-3′ → H-1′a,b, H_2_-2′, H_3_-4′; C-4′ → H_2_-2′, H_2_-3′; EIMS *m*/*z* 294 [M]^+^ (3), 279 (3), 220 (7), 205 (7), 187 (5), 161 (9), 141 (100), 121 (7), 105 (13), 95 (10), 85 (44); HREIMS *m*/*z* 294.2520 [M]^+^ (calcd for C_19_H_34_O_2_, 294.2559).

*(2S,9R)-2-(2′-phenylethoxy)clovan-9-ol* (**2f**): needles (CHCl_3_); 78–80 °C; ${\left[\alpha \right]}_{D\;}^{25}\;$+ 16 (*c* 3.4, CDCl_3_); IR (film) *ν*_max_ 3526, 2926, 2862, 1456, 1106, 750, 698 cm^−1^; ^1^H NMR data (CDCl_3_, 400 MHz), see Table 2; ^13^C NMR data (CDCl_3_, 100 MHz), see Table 3; HMBC (selected correlations) C-1 → H-2α, H-3α, H-12a, H-12b; C-2 → H-3α, H-3β, H-1′a,b; C-15 → H-7a, H-7b, H-12a; C-1′ → H-2α, H_2_-2′; C-2′ → H-1′a,b, H-4′, H-8′; C-3′ → H-1′a,b, H_2_-2′; C-4′, C-8′ → H_2_-2′; HRESIMS *m*/*z* 365.2467 [M+Na]^+^ (calcd for C_23_H_34_O_2_Na, 365.2453), *m*/*z* 325.2521 [M+H-H_2_O]^+^ (calcd for C_23_H_33_O, 325.2531), *m*/*z* 221.1890 [M+H-C_8_H_10_O]^+^ (calcd for C_15_H_25_O, 221.1905).

*(2S,9R)-2-(2′-phenoxyethoxy)clovan-9-ol* (**2g**): yellow oil; ${\left[\alpha \right]}_{D\;}^{25}\;$+ 5 (*c* 3.4, CDCl_3_); IR (film) *ν*_max_ 3436, 2928, 2864, 1600, 1496, 752, 690 cm^−1^; ^1^H NMR data (CDCl_3_, 400 MHz), see Table 2; ^13^C NMR data (CDCl_3_, 100 MHz), see Table 3; HMBC (selected correlations) C-1 → H-3α, H-3β, H-5β, H-12a, H-12b; C-2 → H-3α, H-3β, H-1′a,b; C-15 → H-7a, H-7b, H-12a; C-1′ → H-2α, H_2_-2′; C-2′ → H-1′a,b; C-3′ → H_2_-2′, H-4′, H-8′, H-5′, H-7′; HRESIMS *m*/*z* 381.2404 [M+Na]^+^ (calcd for C_23_H_34_O_3_Na, 381.2406), *m*/*z* 341.2505 [M+H-H_2_O]^+^ (calcd for C_23_H_33_O_2_, 341.2481), *m*/*z* 221.1898 [M+H-C_8_H_10_O_2_]^+^ (calcd for C_15_H_25_O, 221.1905).

^1^H-NMR and ^13^C-NMR spectra can be found as Supplementary Materials for compounds **2b**, **2d**, **2f** and **2g** (Figures S1–S8 and S1a–S8a).

#### 2.2.2. Synthesis of (2*S*,9*R*)-2-(2′-(p-Nitrophenyl)Ethoxy)Clovan-9-ol (**2h**) and (2*S*,9*R*)-2-(2′-(p-Nitrophenoxy)Ethoxy)Clovan-9-ol (**2i**)

We dissolved 500 mg (2.27 mmol) of (-)-caryophyllene oxide by stirring in a solution of either 2-(p-nitrophenyl)ethanol (500 mg, 2.99 mmol) or 2-(p-nitrophenoxy)ethanol (600 mg, 3.27 mmol) in propan-2-one (5 mL) at room temperature. Then, 60 mg (0.47 mmol) of TCNE was added and the reaction was monitored by TLC. After 21 h the solvent was evaporated under reduced pressure. Purification by column chromatography of the crude reaction mixture on silica gel, eluted with increasing gradients of ethyl acetate in petroleum ether, and combined with HPLC purification, yielded compound **2h** (54 mg, 0.14 mmol, 6%) or compound **2i** [38] (64 mg, 0.16 mmol, 7%; see revised assignment of ^1^H and ^13^C NMR spectra and selected HMBC correlations in the Supporting Materials section, Table S1) (Figure 2).

*(2S,9R)-2-(2′-(p-nitrophenyl)ethoxy)clovan-9-ol* (**2h**): yellow oil; ${\left[\alpha \right]}_{D\;}^{25}\;$+ 26 (c 3.4, CDCl_3_); IR (film) *ν*_max_ 3430, 2946, 2926, 2860, 1510, 1344, 1108, 852 cm^−1^; ^1^H NMR (CDCl_3_, 400 MHz), see Table 2; ^13^C NMR (CDCl_3_, 100 MHz), see Table 3; HMBC (selected correlations): C-1 → H-2α, H-3α, H-12a; C-2 → H-3α, H-3β, H-5β, H-12b, H-1′a,b; C-9 → H_3_-15β, H-7a; C-15 → H-7a, H-7b; C-1′ → H-2α, H_2_-2′; C-2′ → H-1′a,b, H-4′, H-8′; C-3′ → H-1′a,b, H_2_-2′, H-5′, H-7′; C-4′, C-8′ → H_2_-2′; C-6′ → H-4′, H-8′; HRESIMS *m*/*z* 410.2300 [M+Na]^+^ (calcd for C_23_H_33_NO_4_Na, 410.2307), *m*/*z* 370.2388 [M+H-H_2_O]^+^ (calcd for C_23_H_32_NO_3_, 370.2382), *m*/z 203.1813 [M+H-C_8_H_9_NO_3_-H_2_O]^+^ (calcd for C_15_H_23_, 203.1800).

^1^H-NMR and ^13^C-NMR spectra can be found as Supplementary Materials for compounds **2h** and **2i** (Figures S9–S12 and S9a–S12a).

### 2.3. General Procedure for the Alcoholysis of (1R,5R,9S)-Caryophylla-4(12),8(13)-Diene-5-ol Catalyzed by Tin(II) Triflate

**Figure 3:**
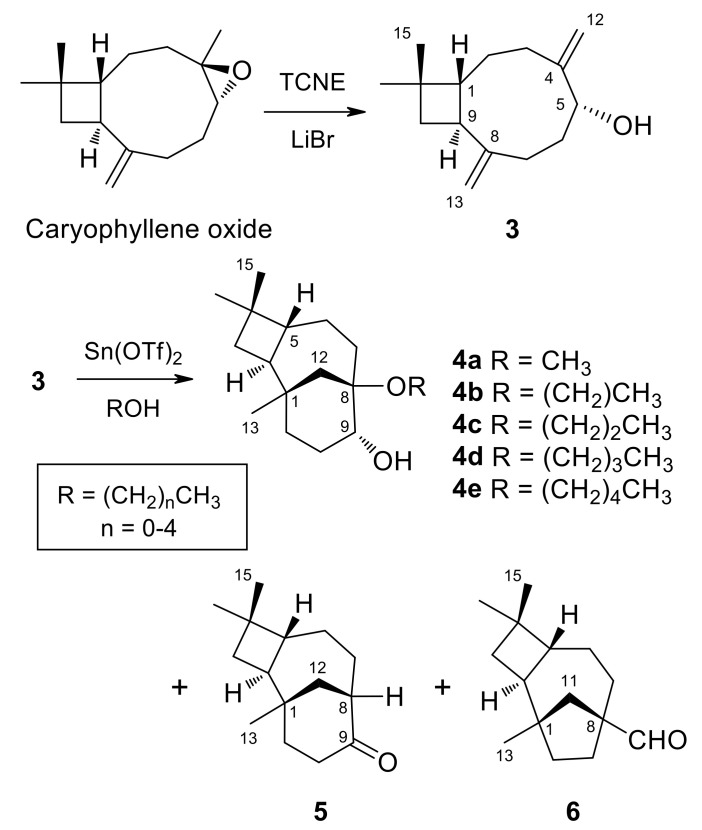
Synthesis of 2-Alkoxyisocaryolan-9-ols (**4a**–**4e**).

(1*R*,5*R*,9*S*)-caryophylla-4(12),8(13)-diene-5-ol (3) (250 mg, 1.14 mmol) dissolved in the alcohol (5 mL) was treated with a catalytic amount of Sn(OTf)_2_ (250 mg, 1.14 mmol) at room temperature under an argon atmosphere. After 24 h, once compound 3 was consumed, as shown by TLC monitoring, the solvent was either evaporated under vacuum or washed with brine. The resulting gum was redissolved in ethyl acetate and dried over anhydrous Na_2_SO_4_. After filtration, evaporation of the solvent afforded a crude reaction product. Purification by column chromatography on silica gel, with increasing gradients of ethyl acetate in petroleum ether, combined with HPLC purification, yielded **4a** [36], **4b** [49], **4c**–**4e**, as well as rearrangement compounds (8*R*,9*R*)-isocaryolan-9-one (5) [36] and (1*S*,2*S*,5*R*,8*S*)-8-methylene-1,4,4-trimethyltricyclo [6.2.1.0^2,5^]undecane-8-carbaldehyde (6) [36] (Figure 3); (reaction conditions, products and yields in Table 4).

#### Spectroscopic Data of Compounds **4c**–**4e**

*(8R,9R)-8-propoxyisocaryolan-9-ol* (**4c**): oil; ${\left[\alpha \right]}_{D\;}^{25}$ -4 (*c* 3.4, CHCl_3_); IR (film) *ν*_max_ 3435, 2947, 1458, 1078, 559 cm^−1^; ^1^H NMR (CDCl_3_, 400 MHz), see Table 5; ^13^C NMR (CDCl_3_, 100 MHz), see Table 5; HMBC (selected correlations): C-8 → H-9β, H-12a, H-12b; C-1′ → H_2_-2′, H_3_-3′; C-2′ → H-1′a,b, H_3_-3′; C-3′ → H-1′a,b, H_2_-2′; EIMS *m*/*z* 280 [M]^+^ (2), 230 (1), 221 (100), 168 (3) 164 (5), 130 (6), 108 (10); HREIMS *m*/*z* 280.2386 [M]^+^ (calcd for C_18_H_32_O_2_, 280.2402).

*(8R,9R)-8-butoxyisocaryolan-9-ol* (**4d**): oil; ${\left[\alpha \right]}_{D\;}^{25}$ -4 (*c* 4.1, CHCl_3_); IR (film) *ν*_max_ 3334, 2945, 1458, 1080, 559 cm^−1^; ^1^H NMR (CDCl_3_, 400 MHz), see Table 5; ^13^C NMR (CDCl_3_, 100 MHz), see Table 5; HMBC (selected correlations): C-8 → H-9β, H-1′a, H-1′b, H-12a, H-12b; C-1′ → H_2_-2′, H_2_-3′; C-2′ → H-1′a,b, H_2_-3′; C-3′ → H-1′a,b, H_2_-2′; C-4’→ H_2_-2′, H_2_-3′; EIMS *m*/*z* 294 [M]^+^ (3), 279 (3), 243 (6), 236 (42), 235 (100), 179 (26), 163 (19), 135 (11), 107 (18), 68 (16), 55 (8); HREIMS *m*/*z* 294.2592 [M]^+^ (calcd for C_19_H_34_O_2_, 294.2559).

*(8R,9R)-8-pentoxyisocaryolan-9-ol* (**4e**): oil; ${\left[\alpha \right]}_{D\;}^{25}$ -2 (*c* 4.9, CHCl_3_); IR (film) *ν*_max_ 3433, 2940, 1457, 1085, 560 cm^−1^; ^1^H NMR (CDCl_3_, 400 MHz), see Table 5; ^13^C NMR (CDCl_3_, 100 MHz), see Table 5; HMBC (selected correlations): C-8 → H-9β, H-12a, H-12b; C-1′ → H_2_-2′, H_2_-3′; C-2′ → H-1′a,b, H_2_-3′; C-3′ → H-1′a,b, H_2_-2′; EIMS *m*/*z* 308 [M]^+^ (15), 292 (12), 237 (16), 218 (20), 180 (14), 164 (28), 141 (60), 93 (26), 71 (100), 68 (2), 43 (42); HREIMS *m*/*z* 308.2733 [M]^+^ (calcd for C_20_H_36_O_2_, 308.2715).

^1^H-NMR and ^13^C-NMR spectra can be found as Supplementary Materials for compounds **4b**–**4e** (Figures S13–S20 and S13a–S20a).

### 2.4. Microorganism and Antifungal Assays

The culture of the *B. cinerea* strain UCA 992 employed in this work was isolated from Domecq vineyard grapes, Jerez de la Frontera, Cádiz, Spain. This culture of *B. cinerea* has been deposited at the Mycological Herbarium Collection (UCA), Facultad de Ciencias, Universidad de Cádiz. Antifungal bioassays were performed by measuring radial growth on agar medium in a Petri dish in the presence of test compounds. Test compounds were dissolved in EtOH to a final compound concentration in the culture medium of 10^−4^ M. Solutions of test compounds were added to glucose-malt-peptone-agar medium (61 g of glucose-malt-peptone-agar per L, pH 6.5–7.0). The final EtOH concentration was identical in both the control and treated cultures. The medium was poured into 6 or 9 cm diameter sterile plastic Petri dishes and a mycelia disk of *B. cinerea,* 5 mm in diameter and cut from an actively growing culture, was placed in the center of the agar plate. Radial growth was measured for five days. The fungal growth inhibition percentage was calculated as ((D1 − D2)/D1) × 100, where D1 is the average diameter of the control colony and D2 is the average diameter of the treated colony. Every concentration was evaluated in triplicate. Fungal growth inhibition percentages are given as averages +/− standard deviations

### 2.5. Bioassay for the Germination and Growth of Lettuce Seeds

*L. sativa* (cv. Grand Rapids) seeds were purchased from Isla Pak, RS, Brazil. All undersized and damaged seeds were discarded. The bioassay was conducted in 100 mm Petri dishes containing Whatman #1 filter paper (90 mm) as a support. *L. sativa* seeds (25 per dish), controls, and test compound solutions (10 mL) at concentrations of 10^−3^ and 10^−5^ M were placed in the dishes. All solutions were prepared with deionized water and the pH values, buffered with 10 mM 2-(*N*-morpholino)ethanesulfonic acid (MES), were adjusted to 6.0–6.5 with NaOH solution. Concentrations lower than 10^−3^ M were obtained by serial dilution. Dishes were wrapped with Parafilm to reduce evaporation and incubated in the dark at 25 °C in an environmental chamber. After 5 days, germinated seeds were counted (a seed was considered to be germinated when the radicle was at least 0.2 mm long) and the lengths of the radicle and shoots were measured using a pachymeter [43]. Dishes were kept at 4 °C during the measurement process to prevent subsequent growth. Osmotic pressure values were measured on a micro-osmometer and ranged between 30 and 38 mOsmolar [45]. The experiments were carried out in triplicate.

The effects of the test compounds on *L. sativa* germination and growth are given as percentage differences from the control, calculated from the differences (in cm) between mean values obtained upon the addition of test compounds and mean values obtained for the control: (seeds grown without addition of tested compounds)/mean values for control × 100. Thus, zero represents the control, positive values represent stimulation of the studied parameter, and negative values represent inhibition. The data were evaluated using Student’s *t*-tests and the differences between the experiment and control were significant at a value of *p* ≤ 0.0517.

## 3. Results and Discussion

The chemical transformations carried out for the preparation of 2-alkoxyclovan-9-ols (**2a**–**2e**) and 2-arylethoxyclovan-9-ols (**2f**–**2i**) are summarized in Figure 2. Compounds **2a**–**2g** were obtained by the treatment of caryophyllene oxide with a catalytic amount of tetracyanoethylene (TCNE), dissolved in the appropriate alcohol, at room temperature [37]. On the other hand, compounds **2h** and **2i** were obtained in a modified procedure, by the treatment of caryophyllene oxide with a catalytic amount of TCNE and 1.3–1.4 equivalents of the appropriate alcohol, dissolved in propan-2-one, at room temperature. These compounds were identified by their ^1^H (see Table 2) and ^13^C (see Table 3) NMR spectra, combined with 1D and 2D NMR techniques and by comparison with authentic samples, where appropriate. All synthesized compounds (**2a**–**2i**) displayed the characteristic pattern of ^1^H and ^13^C NMR signals of clovan-9-ols, bearing different chains at carbon C-2. The compounds **2a** [37], **2c** [48], **2e** [48], and **2i** [24] were previously described in the literature. A revised assignment of ^1^H and ^13^C NMR spectra for compound **2i** together with selected HMBC correlations can be found in the Supporting Information section.

HREIMS of compounds **2b** and **2d** gave molecular ion peaks at *m*/*z* 266.2268 and 294.2520, respectively, which were consistent with the molecular formulas C_17_H_30_O_2_ and C_19_H_34_O_2_. Compound **2b** showed ^1^H NMR signals at δ_H_ 3.56-3.43 (2H) and 1.16 (3H, t, *J* = 7.0 Hz), corresponding to an ethoxy chain at C-2. On the other hand, compound **2d** showed ^1^H NMR signals at δ_H_ 3.44–3.39 (2H), 1.51 (2H, m), 1.36 (2H, m), and 0.89 (3H, t, *J* = 7.3 Hz), assignable to a butoxy chain at C-2. These data led to the assignment of the structures for compounds **2b** and **2d** as (2*S*,9*R*)-2-ethoxyclovan-9-ol and (2*S*,9*R*)-2-butoxyclovan-9-ol, respectively.

Compound **2f** showed ^1^H NMR signals at δ_H_ 3.63–3.53 (2H), 2.79 (2H, t, *J* = 7.2 Hz), and 7.23–7.10 (5H), which suggested the presence of a phenylethoxy moiety. Its HRESIMS gave a sodium adduct ion peak at *m*/*z* 365.2467 [M+Na]^+^, which is consistent with the formula C_23_H_34_O_2_ + Na for the molecular ion. These data led to the assignment of the structure for compound **2f** as (2*S*,9*R*)-2-(2′-phenylethoxy)clovan-9-ol.

The spectroscopy data of compound **2g** were very similar to those of **2f**. A comparison of the ^1^H NMR spectra of both compounds showed as the main difference, the deshielding of the signal corresponding to H-2’ from 2.79 ppm in **2f** to 4.03 ppm in **2g** (Table 2). This, together with the presence in the ^13^C NMR spectrum of two methylenes, one of them bearing an oxygen function, and five aromatic methines, in addition to the fifteen characteristic carbons of the clovane skeleton (Table 3), pointed to the presence of a phenoxyethoxy moiety at C-2. On the other hand, HRESIMS of compound **2g** gave a sodium adduct ion peak at *m*/*z* 381.2404 [M+Na]^+^, which is consistent with the formula C_23_H_34_O_3_ + Na. These data led to the assignment of the structure for compound **2g** as (2*S*,9*R*)-2-(2′-phenoxyethoxy)clovan-9-ol.

Compound **2h** showed a sodium adduct ion at *m*/*z* 410.2300 [M+Na]^+^, consistent with the formula C_23_H_33_NO_4_ + Na, as deduced from the HRESIMS analysis. This, together with ^1^H NMR signals at δ_H_ 7.38 (2H, d, *J* = 8.7 Hz) and 8.12 (2H, d, *J* = 8.7 Hz) and infrared absorptions at 1510 and 1344 cm^−1^, suggested the presence of a nitro-aromatic moiety. These data supported the assignment of the structure of compound **2h** as (2*S*,9*R*)-2-(2′-(*p*-nitrophenyl)ethoxy)clovan-9-ol.

On the other hand, the treatment of (1*R*,5*R*,9*S*)-caryophylla-4(12),8(13)-dien-5-ol (3) with TCNE in methanol leads to (8*R*,9*R*)-8-methoxyisocaryolan-9-ol (**4a**), together with (8*R*,9*R*)-isocaryolan-9-one (5) and (1*S*,2*S*,5*R*,8*S*)-8-methylene-1,4,4-trimethyltricyclo[6 .2.1.0^2,5^]undecane-8-carbaldehyde (6) [36]. Extension of this procedure to higher alcohols led to inseparable mixtures. Alternatively, the treatment of caryophylladienol 3 with a catalytic amount of Sn(OTf)_2_, dissolved in the appropriate alcohol (from methanol to pentan-1-ol), led to the preparation of a series of (8*R*,9*R*)-8-alkoxyisocaryolan-9-ols (**4a**–**4e**), with yields ranging from 34% to 42%, together with variable amounts of isocaryolanone 5 and aldehyde 6 (Figure 3). These compounds were identified by their ^1^H and ^13^C NMR spectra (see Table 5), combined with 1D and 2D NMR techniques and by comparison with authentic samples, where appropriate. While compound **4a** was previously described by us [36], compound **4b** was reported as rumphellol B, a metabolite of the gorgonian coral *Rumphella antipathies* [49].

(8*R*,9*R*)-8-Alkoxyisocaryolan-9-ols **4c** to **4e** displayed the characteristic pattern of ^1^H and ^13^C NMR signals of this family of compounds (Table 5). So, compound **4c** showed signals at δ_H_ 3.56 (1H, dd, *J* = 10.8, 6.3 Hz), corresponding to H-9β, a multiplet between 3.40–3.28 ppm, assigned to the methylene C-1′ of the lateral chain at C-8, and three singlet signals integrating for three protons at δ_H_ 0.97, 0.96, and 0.79 ppm, characteristic of the methyl groups C-13 to C-15. In addition, it can be observed there was a triplet signal integrating for three protons at δ_H_ 0.88 (*J* = 7.4 Hz), which was assigned to the methyl group of the chain at C-8, and two doublets, at δ_H_ 1.87 and 0.89 (*J* = 13.0 Hz), corresponding to each of the protons on carbon C-12. These data led to the assignment of the structure for compound **4c** as (8*R*,9*R*)-8-propoxyisocaryolan-9-ol. All the carbons and their associated proton signals were assigned using the COSY, HSQC and HMBC spectra.

HREIMS of compounds **4d** and **4e**, obtained as colorless oils, indicated the presence of molecular ions at *m*/*z* 294.2592 and 308.2733, which corresponded with the molecular formulas of C_19_H_34_O_2_ and C_20_H_36_O_2_, consistent with the structures proposed respectively, for compounds **4d** and **4e**.

The ^1^H NMR signals of compounds **4d** and **4e** resembled those of the previously described (8*R*,9*R*)-8-alkoxyisocaryolan-9-ols **4a** to **4c**. On the other hand, their ^13^C NMR and HSQC spectra showed significant differences, in addition to the characteristic 15 carbons of the isocaryolane skeleton. While compound **4d** presented three methylenes, one of them bearing an oxygen function, and a methyl group, compound **4e** possessed a methyl group and four methylenes, one of them bearing an oxygen function, which is consistent for the presence of a butoxy chain for compound **4d** and a pentoxy chain in compound **4e**. These observations, together with a careful analysis of the COSY and HMBC experiments, led to the identification of compound 4d as (8*R*,9*R*)-8-butoxyisocaryolan-9-ol and of compound **4e** as (8*R*,9*R*)-8-pentoxyisocaryolan-9-ol.

**Figure 4:**
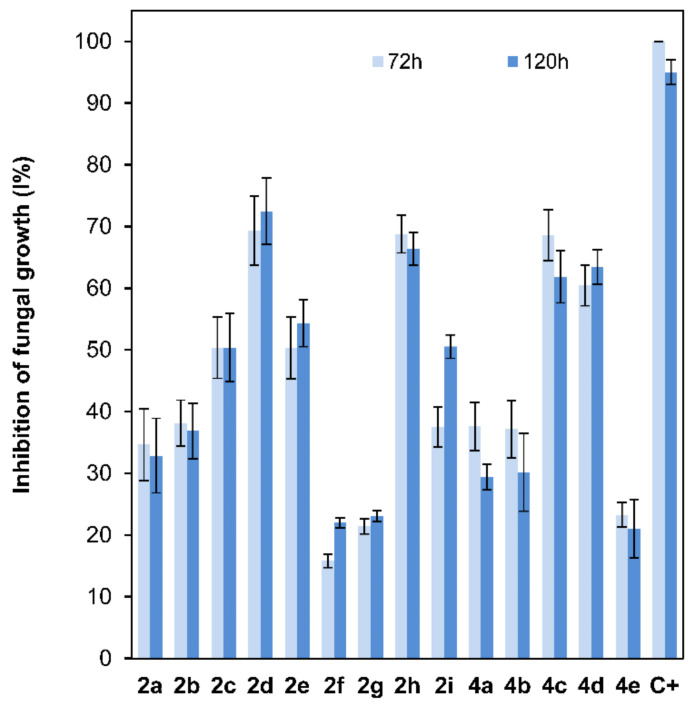
Comparison of the fungal growth inhibition percentage (*B. cinerea*, 72 and 120h*)* among compounds **2a**–**2e** (10^−4^ M dose; 25 ppm **2a**, 27 ppm **2b**, 28 ppm **2c**, 29 ppm **2d**, and 31 ppm **2e**), **2f**–**2i** (10^−4^ M dose; 34 ppm **2f**, 35 ppm **2g**, 38 ppm **2h**, and 40 ppm **2i**), **4a**–**4e** (10^−4^ M dose; 25 ppm **4a**, 27 ppm **4b**, 28 ppm **4c**, 29 ppm **4d**, and 31 ppm **4e**) and dichlofluanid (**C+**) (10^−4^ M dose; 33 ppm **C+**).

The antifungal properties of compounds **2a**–**2i** and **4a**–**4e**, at a 10^−4^ M dose, which is a lower dose than the one previously reported for 2-alkoxyclovan-9-ols [38], were determined against the growth of *B. cinerea* using the poisoned food technique [50]. The commercial fungicide Euparen^®^ (dichlofluanid) was used as a standard for comparison in this test. Several levels of inhibition were observed (Figure 4). For compounds **2a**–**2e**, where the side chain at C-2 varies from methoxy to pentoxy substituents, a tendency of increasing activities was observed, reaching a maximum for (2*S*,9*R*)-2-butoxyclovan-9-ol (**2d**) (Figure 4). These results were consistent with the suggested predictions resulting from a QSAR model published previously for this class of compounds [38]. See a discussion on the correlation between the antifungal activity and log *P* below.

On the other hand, the antifungal activities of clovan-9-ol derivatives *O*-substituted at C-2, do not depend only on the lipophilic character of the side chain [38]. For instance, as we have described in our previous work [38], compounds including nitrogen atoms in this side chain at C-2, such as (2*S*,9*R*)-2-(2′-(*p*-nitrophenoxy)ethoxy)clovan-9-ol (**2i**), display good levels of activity at doses above 50 ppm. In order to evaluate the different contributions of the structural components of the aryl substituent on the antifungal activity, compounds **2f**–**2i** were prepared, as described above, and their antifungal properties were compared with those of compounds **2a**–**2e** (Figure 4). Compounds **2f** and **2g**, which do not present a *p*-nitro group, displayed antifungal activities lower than those of (2*S*,9*R*)-2-methoxyclovan-9-ol (**2a**), the less active compound on the homologous series described above, while *p*-nitro containing compounds showed increased levels of activity. The most active compound of this series, (2*S*,9*R*)-2-(2′-(*p*-nitrophenyl)ethoxy)clovan-9-ol (**2h**), presented an activity profile comparable to the one of (2*S*,9*R*)-2-butoxyclovan-9-ol (**2d**), the most active compound in the above described homologous series (Figure 4), which is an improvement on previously described data [38], where a higher concentration was used (50 ppm). Compounds **2a** and **2i**, also evaluated in a previous study [38], are included here for comparison purposes.

To shed some light on the influence of the length of the alkoxy chain at C-8 on the antifungal activity of isocaryolane derivatives, a structural feature which is expected to undergo metabolism by *B. cinerea* [41,43], a parallel study of the antifungal activities of compounds **4a**–**4e** on the growth of *B. cinerea* was carried out. Compounds (8*R*,9*R*)-8-propoxyisocaryolan-9-ol (**4c**) and (8*R*,9*R*)-8-butoxyisocaryolan-9-ol (**4d**) were the most active compounds in this series. The activity profiles observed for both compounds were similar to the ones shown for (2*S*,9*R*)-2-butoxyclovan-9-ol (**2d**) and (2*S*,9*R*)-2-(2′-(*p*-nitrophenyl)ethoxy)clovan-9-ol (**2h**) (Figure 4).

Most of the active compounds in this study (**2d**, **2h**, **4c** and **4d**) present a calculated [51] log *p* near to the upper limit for this parameter, established by the Lipinsky “rule of five” for the oral availability of drugs—most molecules which show activity via oral administration have log *p* < 5, molecular weight <500 and should contain no more than 10 hydrogen bond acceptors and less than 5 hydrogen bond donors—[52] (compound **2d**, log *p* = 4.499; compound **2h**, log *p* = 4.824; compound **4c**, log *p* = 4.310 and compound **4d**, log *p* = 4.870). Compounds with longer lipophilic aliphatic chains like **2e** and **4e** show a decreased activity, presenting log *p* values above five (compound **2e**, log *p* = 5.004; compound **4e**, log *p* = 5.370). For 2-alkyloxyclovan-9-ols **2a**–**2c**, a roughly linear correlation between log *p* values and activity (Figure 4) can be established (compound **2a**, log *p* = 3.061; compound **2b**, log *p* = 3.437; compound **2c**, log *p* = 3.939). This is not the case for either 2-arylalkyloxyclovan-9-ols (**2f**–**2i**) or 8-alkyloxyisocaryolan-9-ols (**4a**–**4d**), where additional factors, like electronic distribution, must be taken in account.

**Figure 5:**
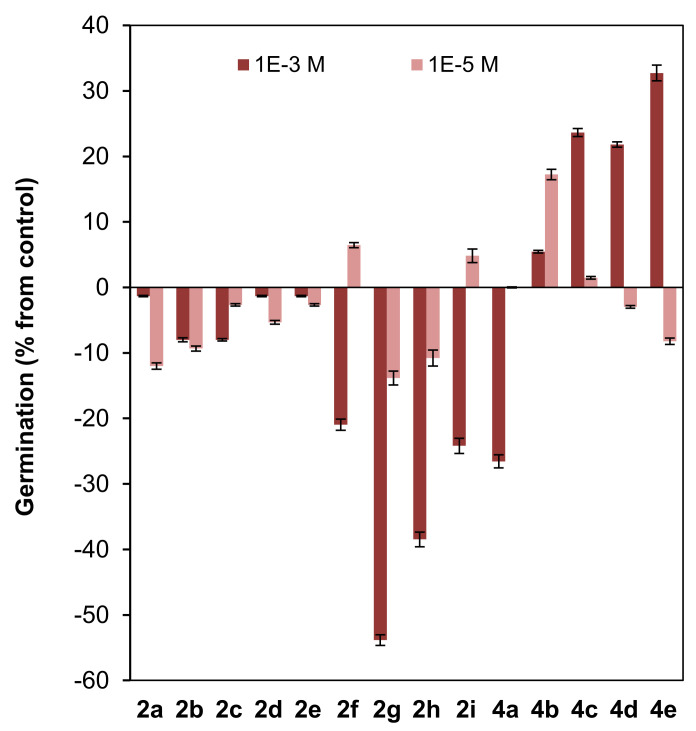
Effect of clovane derivatives **2a**–**2i** and isocaryolane derivatives **4a**–**4e** on the germination of *L. sativa* (see Figure 2 and Figure 3). Values are presented as percentage differences from the control, where a positive value represents stimulation of growth and a negative value represents inhibition.

**Figure 6:**
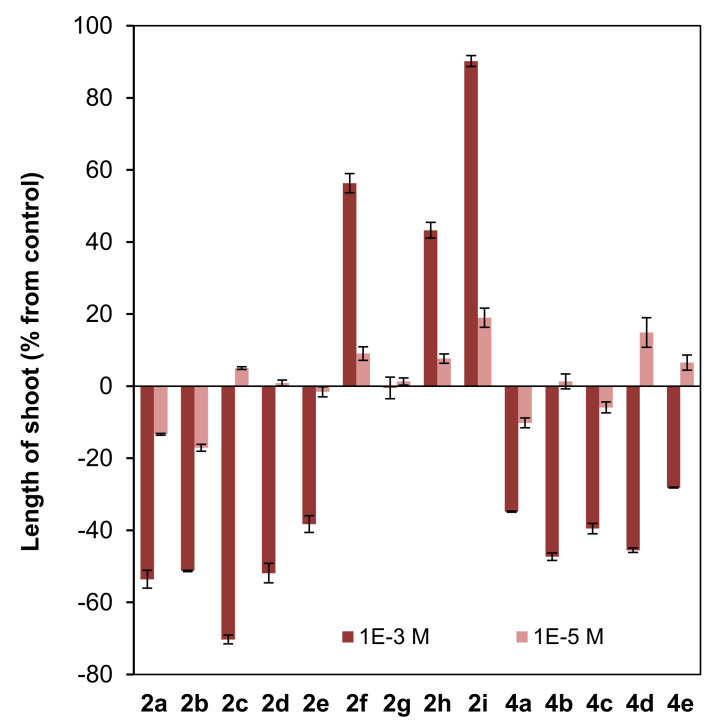

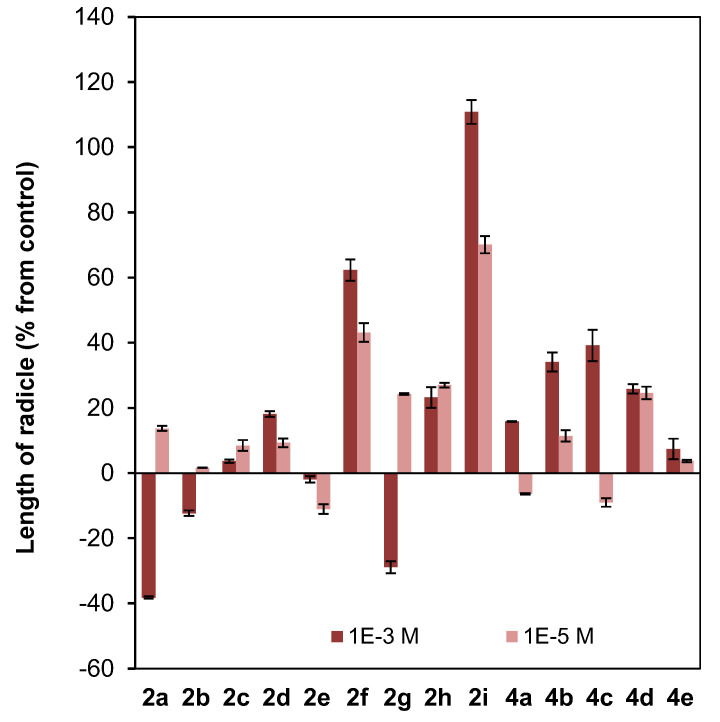
Effect of clovane derivatives **2a**–**2i** and isocaryolane derivatives **4a**–**4e** on radicle and shoot growth of *L. sativa* (see Figure 2 and Figure 3). Values are presented as percentage differences from the control, where a positive value represents stimulation of growth and a negative value represents inhibition.

The effects of previously reported (2*S*,9*R*)-2-metoxyclovan-9-ol (**2a**) [23], (2*S*,9*R*)-2-propoxyclovan-9-ol (**2c**) [48], (2*S*,9*R*)-2-pentoxyclovan-9-ol (**2e**) [48], (2*S*,9*R*)-2-(2′*-(p*-nitrophenoxy)ethoxy)clovan-9-ol (**2i**) [38], (8*R*,9*R*)-8-methoxyisocaryolan-9-ol (**4a**) [36] and (8*R*,9*R*)-8-ethoxyisocaryolan-9-ol (**4b**) [32], as well as the clovane derivatives **2b**, **2d**, and **2f**–**2h**, and isocaryolane derivatives **4c**–**4e**, prepared as described above, were evaluated on the germination (Figure 5) and radicle and shoot growth (Figure 6) of *L. sativa* (lettuce). *L. sativa* seeds are widely used to evaluate phytotoxicity [53,54,55] because of their ready availability and their suitable germination characteristics [45]. Concentrations of 10^−3^ M and 10^−5^ M were evaluated, as they encompassed the doses used for the evaluation of the inhibition of growth of *B. cinerea* (10^−4^ M).

A low inhibitory effect on germination was observed for the homologous series of clovane derivatives **2a**–**2e**, with a maximum effect found for ethoxy and propoxy derivatives (**2b** and **2c**) (Figure 5).

On the other hand, the introduction of an aromatic moiety in the side chain of clovane derivatives (compounds **2f**–**2i**), sharply increased the inhibitory effect on germination at the highest concentration used (10^−3^ M). Furthermore, this effect is exacerbated when either a nitro or an alkoxy moiety is connected to the aromatic ring (see compounds **2g** and **2h**). Noteworthily, the simultaneous presence of both moieties, arranged in a 1,4 substitution pattern, led to a cancellation of the above mentioned effects, as germination inhibition by compound **2i** is similar to that shown by (2*S*,9*R*)-2-(2′-phenylethoxy)clovan-9-ol (**2f**).

Interestingly, and referring also to the highest concentration evaluated (10^−3^ M), while (8*R*,9*R*)-8-methoxyisocaryolan-9-ol (**4a**) showed a germination inhibitory effect comparable to that of the clovane derivatives **2f** and **2i**, an opposite effect was observed for the homologous series of isocaryolane derivatives **4b**–**4e**, where the maximum stimulatory effect was observed for (8*R*,9*R*)-8-pentoxyisocaryolan-9-ol (**4e**).

Different patterns were shown for the effect of clovane derivatives on radicle and shoot growth (Figure 6). On one hand, alkoxyclovanols **2a**–**2e** showed a general inhibitory effect on shoot growth at the highest concentration evaluated (10^−3^ M), with a maximum inhibitory effect observed for (2*S*,9*R*)-2-propoxyclovan-9-ol (**2c**). A different response was found for this homologous series (**2a**–**2e**) on radicle growth, where a progression on the length of the alkyl chain leads to activity changes from inhibitory to stimulatory. A maximum stimulatory effect was found for (2*S*,9*R*)-2-butoxyclovan-9-ol (**2d**), the most effective alkoxyclovanol of the homologous series **2a**–**2e**, for the inhibition of the growth of *B. cinerea* (10^−4^ M dose, see Figure 4). On the other hand, while there was almost no effect of (2*S*,9*R*)-2-(2′-phenoxyethoxy)clovan-9-ol (**2g**) on shoot growth, and a moderate inhibitory effect on radicle growth, there was a general stimulatory effect, on both shoot and radicle growth, for compounds **2f**, **2h**, and **2i**, where **2i** showed the greatest stimulatory effect. Consequently, given that (2*S*,9*R*)-2-(2′-(*p*-nitrophenyl)ethoxy)clovan-9-ol (**2h**) showed an inhibitory effect on the growth of *B. cinerea* of a similar magnitude to that of the most effective alkoxyclovanol of the homologous series (**2d**) (10^−4^ M dose, see Figure 4), this made compound **2h** the most potentially useful of the clovane derivatives evaluated so far, as the inhibition of germination was compensated with stimulation of both radicle and shoot growth. Compound **2d**, with a comparable inhibitory effect on *B. cinerea* growth (see Figure 4), showed inhibitory effects on both germination and shoot growth and only a moderate stimulatory effect on radicle growth (see Figure 6).

On the other hand, different trends could be found for the isocaryolane derivatives evaluated. Along the homologous series **4a**–**4e**, an increase of the stimulation of germination could be found at the highest dose evaluated (10^−3^ M), as discussed above (Figure 5). Additionally, also at the 10^−3^ M dose, different tendencies could be found for radicle and shoot growth (Figure 6); while a general stimulatory effect was apparent for radicle growth, with a maximum effect found for (8*R*,9*R*)-8-propoxyisocaryolan-9-ol (**4c**), a general inhibitory effect on shoot growth was observed. Given that (8*R*,9*R*)-8-propoxyisocaryolan-9-ol (**4c**) had inhibitory activity on the growth of *B. cinerea* of a similar magnitude to the most effective alkoxyclovanol, compound **2d** (see Figure 4), this made compound **4c** the most potentially useful of the isocaryolane derivatives evaluated so far.

## 4. Conclusions

In conclusion, a high activity against *B. cinerea* can be found in 2-alkoxyclovan-9-ols (**2a**–**2e**), 2-arylalkoxyclovan-9-ols (**2f**–**2i**) or 8-alkoxyisocaryolan-9-ols (**4a**–**4d**) at a 10^−4^ M dose, depending on the nature of the substituent side chain (see Figure 4), where compounds **2d**, **2h**, **4c** and **4d** show the best activities, even improving on those described before [38].

On the other hand, in the dose range 10^−3^ M to 10^−5^ M, different phytotoxicity profiles can be associated to the structural classes mentioned above. In general terms, 2-alkoxyclovan-9-ols (**2a**–**2e**) exert a phytotoxic effect, especially on shoot growth (see Figure 6). 2-Arylalkoxyclovan-9-ols (**2f**–**2i**) are phytotoxic on germination (see Figure 5), but stimulate shoot and radicle growth (Figure 6), which would make them suitable candidates for post-germination applications, with compound **2h** being the best candidate. Finally, 8-alkoxyisocaryolan-9-ols (**4a**–**4d**) inhibit shoot growth (Figure 6), but stimulate germination (Figure 5) and radicle growth (Figure 6), which would make them suitable candidates for both pre- and post-germination applications, with compounds **4c** and **4d** being the best candidates.

## Figures and Tables

**Table 1:** Alcoholysis of Caryophyllene Oxide Catalyzed by TCNE. Reaction Conditions, Products and Yields.

Epoxide (mg)	Alcohol (mL)	TCNE (mg)	Time (hr)	Product (Yield)
1019	CH_3_CH_2_OH (20)	63	36	**2b** (15%)
1013	CH_3_(CH_2_)_3_OH (20)	61	36	**2d** (11%)
500	PhCH_2_CH_2_OH (3)	58	103	**2f** (10%)
500	PhO(CH_2_)_2_OH (0.5)	60	114	**2g** (9%)

**Table 2:** ^1^H NMR Spectroscopic Data (CDCl_3_, 400 MHz) for Compounds **2b**, **2d**, and **2f**–**2h**.

	2b	2d	2f	2g	2h
Position	δ_H_, Mult (*J* in Hz)	δ_H_, Mult (*J* in Hz)	δ_H_, Mult (*J* in Hz)	δ_H_, Mult (*J* in Hz)	δ_H,_ Mult (*J* in Hz)
2α	3.40, dd (10.6, 5.6)	3.36, dd (10.3, 5.6)	3.32, dd (10.2, 5.7)	3.44, dd (10.3, 5.6)	3.34, dd (9.8, 5.6)
3α	1.66, dd (11.8, 5.6)	1.65, dd (11.9, 5.5)	1.58, dd (11.9, 5.7)	1.64, dd (12.0, 5.6)	1.61, dd (11.8, 5.6)
3β	1.48, dd (11.8, 10.6)	1.48, m	1.40, dd (11.9, 10.3)	1.47, dd (12.0, 10.3)	1.41, dd (11.8, 9.8)
5β	1.38, m	1.38, m	1.31, m	1.34, m	1.38, m
6a	1.38, m	1.40, m	1.35–1.27, m	1.33, m	1.40–1.20, m
6b	1.31, m	1.30, m	1.35–1.27, m	1.23, m	1.40–1.20, m
7a	1.38, m	1.36, m	1.29, m	1.30, m	1.35, m
7b	1.09, m	1.10, m	1.02, m	1.03, m	1.08, m
9β	3.30, brs	3.29, brs	3.21, brs	3.23, brs	3.29, brs
10α	1.58, m	1.59, m	1.50, m	1.51, m	1.56, m
10β	1.96, tdd (14.2, 4.8, 3.3)	1.96, tdd (14.2, 5.0, 3.2)	1.89, tdd (14.2, 5.9, 3.5)	1.90, tdd (14.3, 5.0, 3.3)	1.93, tdd (14.1, 6.8, 4.5)
11a	1.69, dd (13.7, 4.8)	1.69, m	1.56, m	1.63, m	1.59, m
11b	1.09, m	1.11, m	1.00, m	1.06, m	1.09, m
12a	1.59, d (12.7)	1.57, d (12.7)	1.43, d (12.9)	1.53, d (12.8)	1.49, d (12.7)
12b	0.97, m	0.96, m	0.86, m	0.91, brd (12.8)	0.91, m
13α	0.83, ^a^ s	0.83, ^b^ s	0.75, ^c^ s	0.79, ^d^ s	0.80, ^f^ s
14β	1.00, ^a^ s	1.00, ^b^ s	0.93, ^c^ s	0.95, ^d^ s	0.96, ^f^ s
15	0.94, s	0.94, s	0.86, s	0.87, s	0.92, s
1′a,b	3.56–3.43	3.44-3.39	3.63-3.53	3.79–3.70	3.70-3.62
2′	1.16, t (7.0)	1.51, m	2.79, t (7.2)	4.03, t (5.1)	2.93, t (6.4)
3′		1.36, m			
4′, 8′		0.89, t (7.3)	7.23–7.10	6.89-6.83 ^e^	7.38, d (8.7)
5′, 7′			7.23–7.10	7.23–7.17 ^e^	8.12, d (8.7)
6′			7.23–7.10	6.87, m	

^a–f^ Interchangeable signals.

**Table 3:** ^13^C NMR Spectroscopic Data (CDCl_3_, 100 MHz) for Compounds **2b**, **2d**, and **2f**–**2h**.

	2b	2d	2f	2g	2h
Position	δ_C_, Type	δ_C_, Type	δ_C_, Type	δ_C_, Type	δ_C_, Type
1	44.1, C	44.3, C	44.3, C	44.3, C	44.3, C
2	88.1, CH	88.3, CH	88.6, CH	89.1, CH	88.9, CH
3	44.9, CH_2_	44.7, CH_2_	44.5, CH_2_	44.6, CH_2_	44.5, CH_2_
4	36.9, C	37.0, C	37.1, C	37.0, C	37.2, C
5	50.6, CH	50.5, CH	50.5, CH	50.5, CH	50.4, CH
6	20.5, CH_2_	20.6, CH_2_	20.6, CH_2_	20.6, CH_2_	20.6, CH_2_
7	33.1, CH_2_	33.2, CH_2_	33.1, CH_2_	33.1, CH_2_	33.1, CH_2_
8	34.7, C	34.7, C	34.7, C	34.7, C	34.6, C
9	75.3, CH	75.3, CH	75.2, CH	75.2, CH	75.1, CH
10	26.0, CH_2_	26.1, CH_2_	26.0, CH_2_	26.0, CH_2_	26.1, CH_2_
11	26.7, CH_2_	26.7, CH_2_	26.7, CH_2_	26.7, CH_2_	26.8, CH_2_
12	36.6, CH_2_	36.5, CH_2_	36.4, CH_2_	36.4, CH_2_	36.4, CH_2_
13	25.3, ^a^ CH_3_	25.4, ^b^ CH_3_	25.4, ^c^ CH_3_	25.4, ^d^ CH_3_	25.4, ^f^ CH_3_
14	31.2, ^a^ CH_3_	31.3, ^b^ CH_3_	31.3, ^c^ CH_3_	31.3, ^d^ CH_3_	31.3, ^f^ CH_3_
15	28.4, CH_3_	28.4, CH_3_	28.4, CH_3_	28.4, CH_3_	28.3, CH_2_
1′	65.8, CH_2_	70.3, CH_2_	71.6, CH_2_	68.9, CH_2_	70.2, CH_2_
2′	15.7, CH_3_	32.3, CH_2_	36.9, CH_2_	67.6, CH_2_	36.7, CH_2_
3′		19.4, CH_2_	139.4, C	158.9, C	147.8, C
4′, 8′		14.0, CH_3_	129.0, 2CH	114.7, ^e^ 2CH	129.9, 2CH
5′, 7′			128.2, 2CH	129.3, ^e^ 2CH	123.3, 2CH
6′			126.0, CH	120.7, CH	146.5, C

^a–f^ Interchangeable signals.

**Table 4:** Alcoholysis of Caryophyllene Oxide Catalyzed by Sn(OTf)_2_. Reaction Conditions, Products and Yields.

Alcohol	Products (Yield)
CH_3_OH	**4a** (42%), **5** (13%), **6** (27%)
CH_3_CH_2_OH	**4b** (39%), **5** (14%), **6** (29%)
CH_3_(CH_2_)_2_OH	**4c** (35%), **5** (16%), **6** (31%)
CH_3_(CH_2_)_3_OH	**4d** (34%), **5** (19%), **6** (42%)
CH_3_(CH_2_)_4_OH	**4e** (35%), **5** (20%), **6** (37%)

**Table 5:** NMR Spectroscopic Data for Compounds **4c**–**4e** in CDCl_3_ (*J* in Hz).

	4c		4d		4e	
Position	δ_H_ (400 MHz)	δ_C_ (100 MHz), Type	δ_H_ (400 MHz)	δ_C_ (100 MHz), Type	δ_H_ (400 MHz)	δ_C_ (100 MHz), Type
1		32.83, C		32.83, ^a^ C		32.88, C
2α	2.08, ddd (11.9, 10.3, 8.0)	36.57, CH	2.07, ddd (12.4, 11.8, 7.9)	36.55, CH	2.07, ddd (12.0, 10.5, 7.9)	36.55, CH
3α	1.44, m	35.52, CH_2_	1.45, m	35.52, CH_2_	1.44, dd (9.8, 7.9)	35.51, CH_2_
3β	1.27, t (10.3)		1.27, t (10.2)		1.26, m	
4		34.88, C		34.88, C		34.87, C
5β	1.72, m	43.96, CH	1.71, m	43.95, CH	1.70, m	43.94, CH
6a	1.50, m	21.70, CH_2_	1.48, m	21.71, CH_2_	1.49, m	21.72, CH_2_
6b	1.61, m		1.61, m		1.61, m	
7a	1.53, m	29.29, CH_2_	1.52, m	29.29, CH_2_	1.53, m	29.28, CH_2_
7b	1.87, m		1.86, m		1.86, m	
8		79.96, C		79.95, C		79.96, C
9β	3.56, dd (10.8, 6.3)	76.86, CH	3.55, dd (11.4, 5.8)	76.85, CH	3.55, dd (11,3, 5.8)	76.82, CH
10a	1.84–1.70, m	27.09, CH_2_	1.83–1.70, m	27.08, CH_2_	1.82–1.70, m	27.10, CH_2_
10b	1.84–1.70, m		1.83–1.70, m		1.82–1.70, m	
11α	1.21, td (13.2, 5.3)	36.65, CH_2_	1.21, td (12.8, 4.9)	36.64, CH_2_	1.20, td (13.6, 5.1)	36.63, CH_2_
11β	1.37, m		1.37, m		1.36, m	
12a	1.87, brd (13.0)	42.70, CH_2_	1.86, brd (12.5)	42.67, CH_2_	1.87, m	42.67, CH_2_
12b	0.89, d (13.0)		0.91, d (12.5)		0.90, m	
13	0.97, s	26.24, CH_3_	0.97, s	26.24, CH_3_	0.96, s	26.23, CH_3_
14α	0.96, s	20.84, CH_3_	0.96, s	20.84, CH_3_	0.95, s	20.83, CH_3_
15β	0.79 s	30.67, CH_3_	0.79, s	30.67, CH_3_	0.78, s	30.66, CH_3_
1′a,b	3.40–3.28	62.68, CH_2_	3.44–3.31	60.68, CH_2_	3.43–3.30	61.03, CH_2_
2′	1.49, m	23.93, CH_2_	1.44, m	32.85, ^a^ CH_2_	1.46, m	30.45, CH_2_
3′	0.88, t (7.4)	10.78, CH_3_	1.33, m	19.47, CH_2_	1.27, m	28.50, CH_2_
4′			0.89, t (7.4)	13.96, CH_3_	1.29, m	22.56, CH_2_
5′					0.89, t (7.0)	14.07, CH_3_

^a^ Interchangeable signals.

## Data Availability

Not applicable.

## Supplementary Materials

The following are available online at https://www.mdpi.com/article/10.3390/jof7121079/s1, Revised assignments of ^1^H and ^13^C NMR spectra for compound **2i**, together with selected HMBC correlations. Table S1. Figures S1–S20a. ^1^H- and ^13^C NMR spectra of compounds **2b**, **2d**, **2f**–**2i**, and **4b**–**4e**.
