# Supplementary material for: Synthesis, Fungitoxic Activity against Botrytis cinerea and Phytotoxicity of Alkoxyclovanols and Alkoxyisocaryolanols
Source: J Fungi (Basel). 2021 Dec 15;7(12):1079. doi: 10.3390/jof7121079 (PMC8709304; doi:10.3390/jof7121079)
Supplement: Supplementary file 1 [file jof-07-01079-s001.zip › jof-1498433-supplementary.pdf]

Supplementary Materials

Synthesis, fungitoxic activity against *Botrytis cinerea* and phytotoxicity of alkoxyclovanols and alkoxyisocaryolanols

Adriana de Almeida Pinto Bracarense,[1,2,#] Jociani Ascari,[1,3,#] Giovanni Gontijo de Souza,[1] Thays Silva Oliveira,[1] Antonio Ruano-González,[4] Ana A. Pinto,[4] Maria Amélia Diamantino Boaventura,[1] Jacqueline Aparecida Takahashi,[1] Isidro G. Collado,[4,5] Rosa Durán-Patrón,[*,4] and Antonio J. Macías-Sánchez[*,4,5]

[1] Departamento de Química, Instituto de Ciências Exatas, Universidade Federal de Minas Gerais, Av. Presidente Antônio Carlos, 6627, 31270-901, Belo Horizonte, MG, Brazil.

[2] Departamento de Química, Centro Federal de Educação Tecnológica de Minas Gerais, Av. Amazonas, 5253, 30421-169, Belo Horizonte, MG, Brazil.

[3] Coordenaçao Ciências Biológicas, Universidade Tecnológica Federal do Paraná, Prolongamento da Rua Cerejeira, s/n, 85892-000 Santa Helena, PR, Brazil.

[4] Departamento de Química Orgánica, Facultad de Ciencias, Universidad de Cádiz, 11510 Puerto Real, Cádiz, Spain.

[5] Instituto de Investigación en Biomoléculas (INBIO), Universidad de Cádiz, 11510 Puerto Real, Cádiz, Spain.

* Correspondence: R D-P rosa.duran@uca.es, AJ M-S antoniojose.macias@uca.es Tel.: (+35 956 012704).

[#] These authors contributed equally to this work.

Table of contents

Revised assignment of ^{1}H and ^{13}C NMR spectra and selected HMBC correlations of compound **2i**. Table S1 ……………………………………………………………..**S5-6**

Figure S1. ^{1}H NMR spectrum of compound **2b** in $CDCl_3$ (400MHz)…………………......**S7**

Figure S1a. Expansion (δ_H 3.7-0.5) of ^{1}H NMR spectrum of compound **2b** in $CDCl_3$ (400 MHz)……………………………………………………………………...……...**S8**

Figure S2. ^{13}C NMR spectrum of compound **2b** in $CDCl_3$ (100 MHz)………...…...**S9**

Figure S2a. Expansion (δ_C 95-10) of ^{13}C NMR spectrum of compound **2b** in $CDCl_3$ (100 MHz)……………………………………………………………………………...**S10**

Figure S3. ^{1}H NMR spectrum of compound **2d** in $CDCl_3$ (400 MHz)………………...**S11**

Figure S3a. Expansion (δ_H 3.7-0.5) of ^{1}H NMR spectrum of compound **2d** in $CDCl_3$ (400 MHz)……………………………………………………………………………....**S12**

Figure S4. ^{13}C NMR spectrum of compound **2d** in $CDCl_3$ (100 MHz)………...…...**S13**

Figure S4a. Expansion (δ_C 95-10) of ^{13}C NMR spectrum of compound **2d** in $CDCl_3$ (100 MHz)……………………………………………...…..………………...**S14**

Figure S5. ^{1}H NMR spectrum of compound **2f** in $CDCl_3$ (400 MHz)………….……...**S15**

Figure S5a. Expansion (δ_H 3.7-0.5) of ^{1}H NMR spectrum of compound **2f** in $CDCl_3$ (400 MHz)………………..………………………………………...……………...**S16**

Figure S6. ^{13}C NMR spectrum of compound **2f** in $CDCl_3$ (100 MHz)………………...**S17**

Figure S6a. Expansion (δ_C 95-10) of ^{13}C NMR spectrum of compound **2f** in $CDCl_3$ (100 MHz)………………..…………………………………………...………...**S18**

Figure S7. ^{1}H NMR spectrum of compound **2g** in $CDCl_3$ (400 MHz)……………………………….………….………………….……………...**S19**

Figure S7a. Expansion (δ_H 4.2-0.5) of ^{1}H NMR spectrum of compound **2g** in $CDCl_3$ (400 MHz)…………………………………………………..………………...**S20**

Figure S8. ^{13}C NMR spectrum of compound **2g** in $CDCl_3$ (100 MHz).....……...…...**S21**

Figure S8a. Expansion (δ_C 95-10) of ^{13}C NMR spectrum of compound **2g** in $CDCl_3$ (100 MHz)……**S22**

Figure S9. ^{1}H NMR spectrum of compound **2h** in $CDCl_3$ (400 MHz)……**S23**

Figure S9a. Expansion (δ_H 4.2-0.5) of ^{1}H NMR spectrum of compound **2h** in $CDCl_3$ (400 MHz)……**S24**

Figure S10. ^{13}C NMR spectrum of compound **2h** in $CDCl_3$ (100 MHz)……**S25**

Figure S10a. Expansion (δ_C 95-10) of ^{13}C NMR spectrum of compound **2h** in $CDCl_3$ (100 MHz)……**S26**

Figure S11. ^{1}H NMR spectrum of compound **2i** in $CDCl_3$ (400 MHz)……**S27**

Figure S11a. Expansion (δ_H 4.5-0.5) of ^{1}H NMR spectrum of compound **2i** in $CDCl_3$ (400 MHz)……**S28**

Figure S12. ^{13}C NMR spectrum of compound **2i** in $CDCl_3$ (100 MHz)……**S29**

Figure S12a. Expansion (δ_C 95-10) of ^{13}C NMR spectrum of compound **2i** in $CDCl_3$ (100 MHz)……**S30**

Figure S13. ^{1}H NMR spectrum of compound **4b** in $CDCl_3$ (400 MHz)……**S31**

Figure S13a. Expansion (δ_H 3.8-0.5) of ^{1}H NMR spectrum of compound **4b** in $CDCl_3$ (400 MHz)……**S32**

Figure S14. ^{13}C NMR spectrum of compound **4b** in $CDCl_3$ (100 MHz)……**S33**

Figure S14a. Expansion (δ_C 95-10) of ^{13}C NMR spectrum of compound **4b** in $CDCl_3$ (100 MHz)……**S34**

Figure S15. ^{1}H NMR spectrum of compound **4c** in $CDCl_3$ (400 MHz)……**S35**

Figure S15a. Expansion (δ_H 3.8-0.5) of ^{1}H NMR spectrum of compound **4c** in $CDCl_3$ (400 MHz)……**S36**

Figure S16. ^{13}C NMR spectrum of compound **4c** in $CDCl_3$ (100 MHz)……**S37**

Figure S16a. Expansion (δ_C 95-10) of ^{13}C NMR spectrum of compound **4c** in $CDCl_3$ (100 MHz)……**S38**

Figure S17. ^{1}H NMR spectrum of compound **4d** in $CDCl_3$ (400 MHz)……………....**S39**

Figure S17a. Expansion (δ_H 3.8-0.5) of ^{1}H NMR spectrum of compound **4d** in $CDCl_3$ (400 MHz)………………..…………………………………………………..**S40**

Figure S18. ^{13}C NMR spectrum of compound **4d** in $CDCl_3$ (100 MHz)..…………...**S41**

Figure S18a. Expansion (δ_C 95-10) of ^{13}C NMR spectrum of compound **4d** in $CDCl_3$ (100 MHz)………………..…………………………………………...…...**S42**

Figure S19. ^{1}H NMR spectrum of compound **4e** in $CDCl_3$ (400 MHz)………..…...**S43**

Figure S19a. Expansion (δ_H 3.8-0.5) of ^{1}H NMR spectrum of compound **4e** in $CDCl_3$ (400 MHz)………………..…………………………………………...…...**S44**

Figure S20. ^{13}C NMR spectrum of compound **4e** in $CDCl_3$ (100 MHz).…….……...**S45**

Figure S20a. Expansion (δ_C 95-10) of ^{13}C NMR spectrum of compound **4e** in $CDCl_3$ (100 MHz)……………………………………………………..………………...**S46**

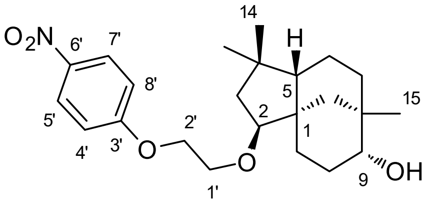

2i

(2S,9R)-2-(2'-(p-nitrophenoxy)ethoxy)clovan-9-ol (***2i***)*:*[1] ^{1}H NMR ($CDCl_3$, 400 MHz), see Table S1; ^{13}C NMR ($CDCl_3$, 100 MHz), see Table S1; HMBC (selected correlations): C-1 → H-2α, H-3α, H-5β, H-12a, H-12b; C-2 → H-3α, H-3β, H-12a, H-12b, H-1'a,b; C-9 → H-7a, H-7b, H-11a, H-12a, H-12b, H_3-15β; C-1' → H-2α, H_2-2'; C-2' → H-1'a,b; C-3' → H_2-2', H-4', H-8', H-5', H-7'; C-6' → H-4', H-8', H-5', H-7'.

[1] Saiz-Urra, L.; Racero, J. C.; Macias-Sanchez, A. J.; Hernandez-Galan, R.; Hanson, J. R.; Perez- Gonzalez, M.; Collado, I. G. *J. Agric. Food Chem.* **2009**, *57*, 2420–2428.

Table S1. ^{1}H NMR ($CDCl_3$, 400 MHz) and ^{13}C NMR ($CDCl_3$, 100 MHz) Spectroscopic Data for Compound 2i

	**2i**	
Position	δ_H, mult (*J* in Hz)	δ_C, type
2α	3.47, dd (10.0, 5.6)	44.38, C
3α	1.67, dd (11.5, 5.6)	89.27, CH
3β	1.51 dd (11.5, 10.5)	44.45, CH_2
4		37.10, C
5β	1.41, m	50.46, CH
6a	1.40, m	20.52, CH_2
6b	1.30, m	
7a	1.37, m	33.00, CH_2
7b	1.10, m	
8		34.65, C
9β	3.29, brs	75.04, CH
10α	1.59, m	26.00, CH_2
10β	1.94, tdd (14.3, 4.6, 3.4)	
11a	1.69, m	26.70, CH_2
11b	1.11, m	
12a	1.57, d (12.8)	36.41, CH_2
12b	0.96, brd (12.8)	
13α	0.84,[a] s	25.35,[b] CH_3
14β	1.00,[a] s	31.22,[b] CH_3
15	0.92, s	28.30, CH_3
1'a,b	3.87-3.79	68.57, CH_2
2'	4.17, t (5.0)	68.48, CH_2
3'		164.03, C
4', 8'	6.96, d (9.3)	114.65, 2CH
5', 7'	8.16,d (9.3)	125.77, 2CH
6'		141.41, C

a-b Interchangeable signals

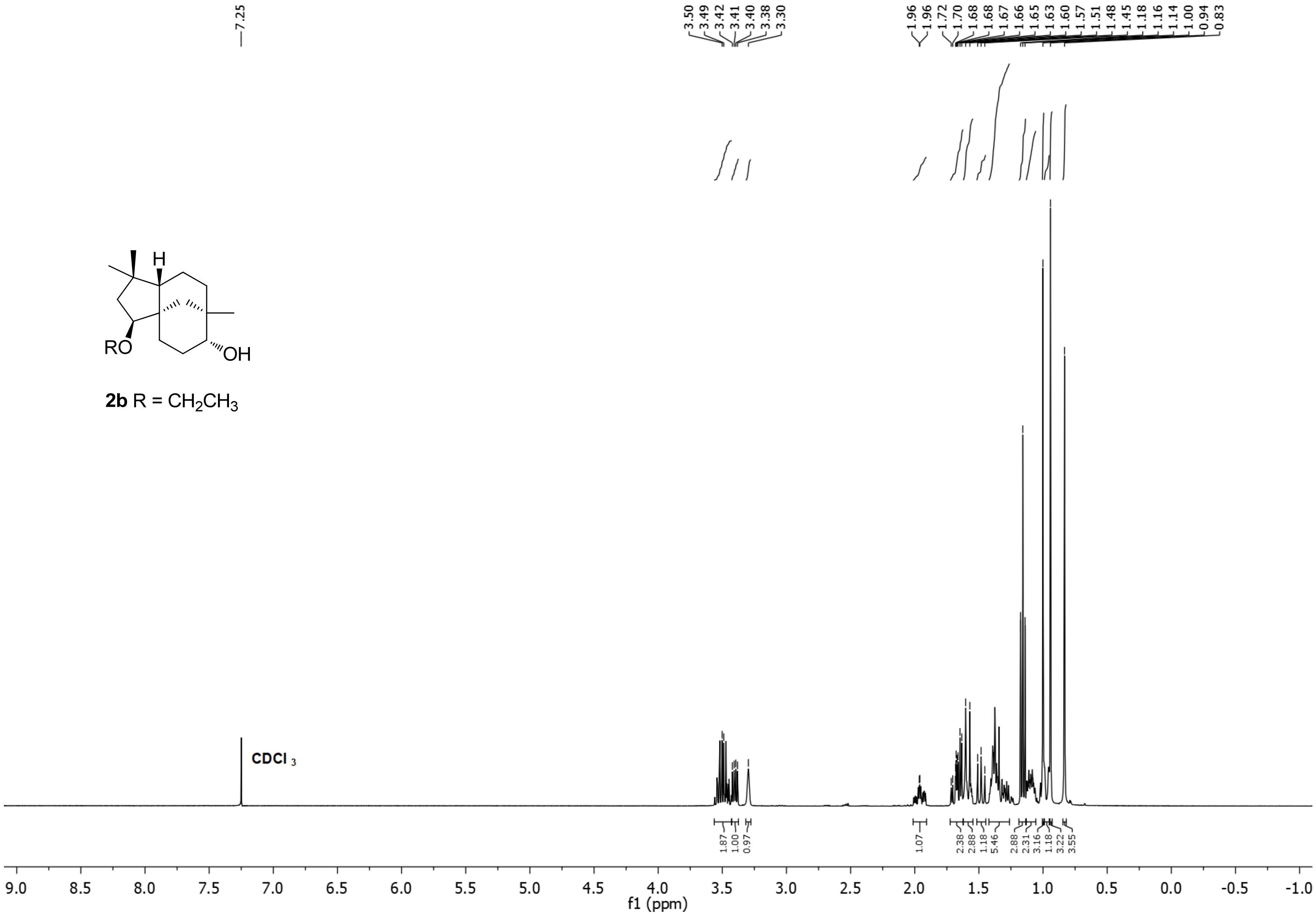

Figure S1. ^{1}H NMR spectrum of compound **2b** in $CDCl_3$ (400 MHz).

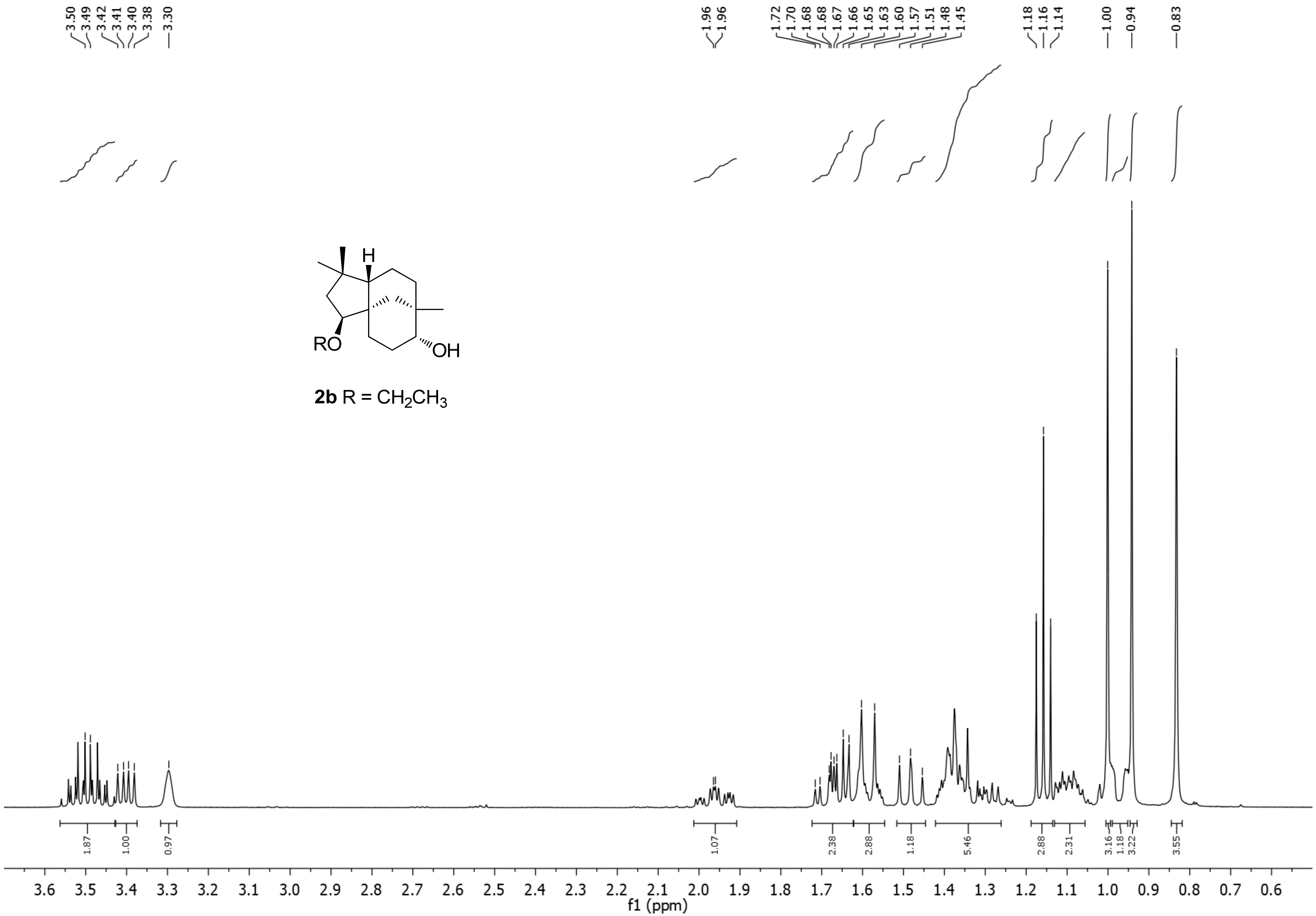

Figure S1a. Expansion (δ_H 3.7-0.5) of ^{1}H NMR spectrum of compound **2b** in $CDCl_3$ (400 MHz).

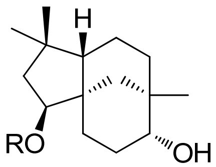

2b R = CH_2CH_3

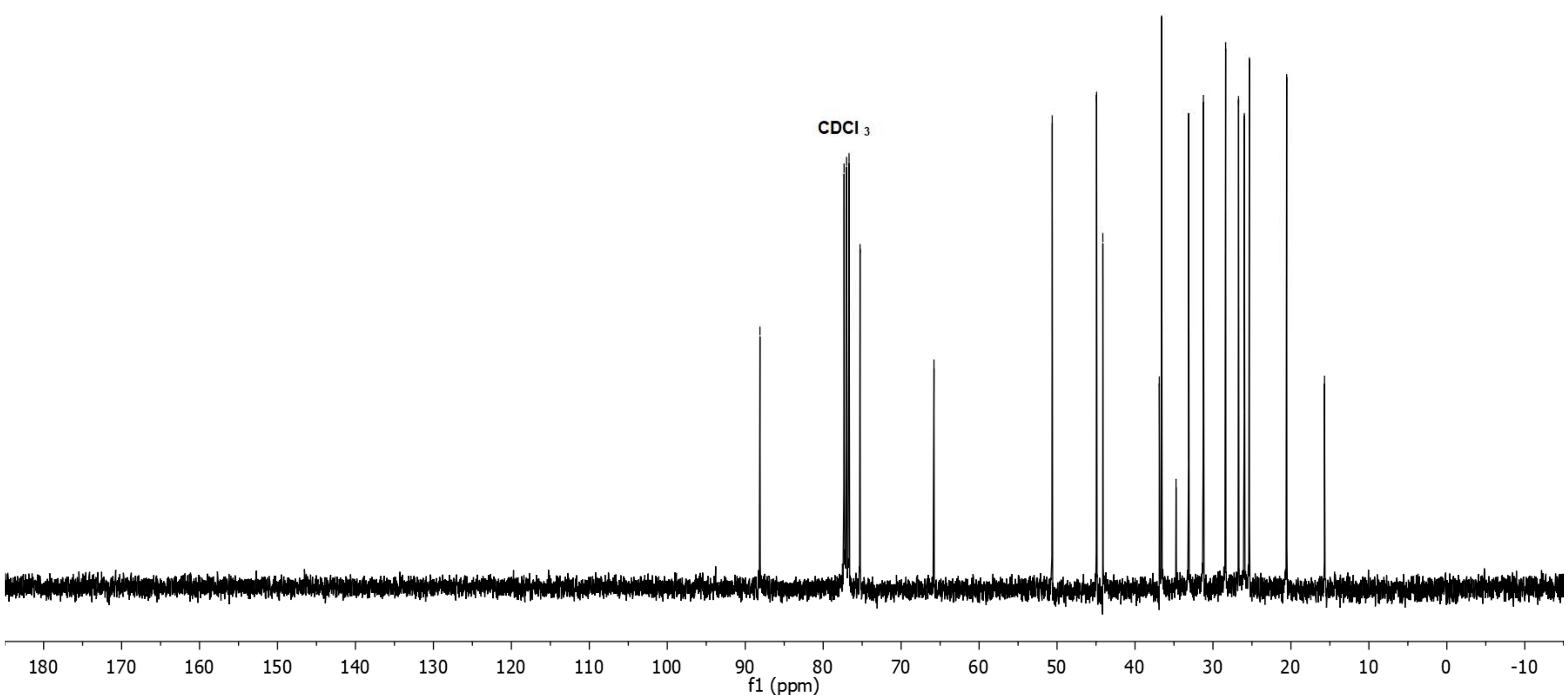

Figure S2. ^{13}C NMR spectrum of compound **2b** in $CDCl_3$ (100 MHz).

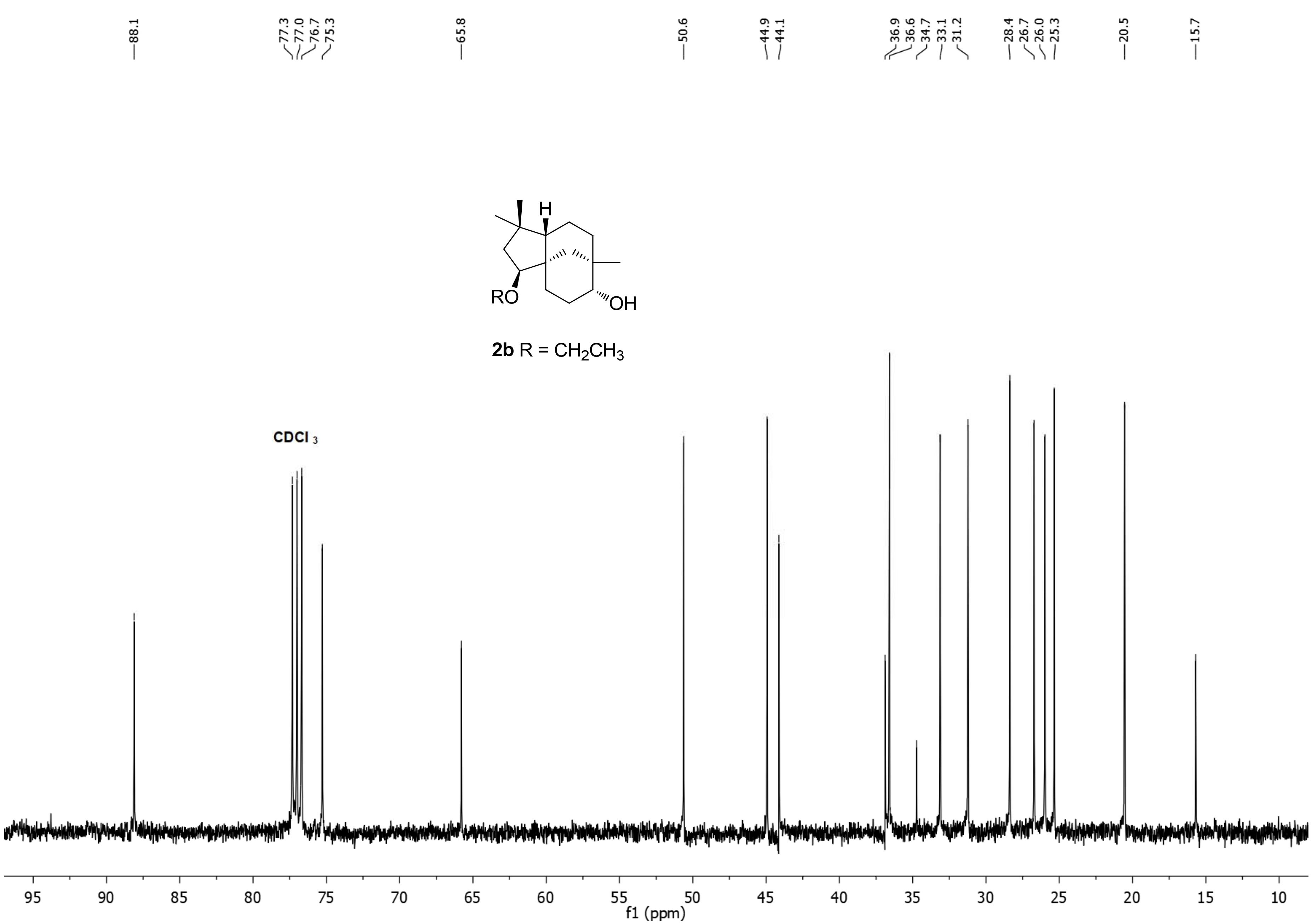

Figure S2a. Expansion (δ_C 95-10) of ^{13}C NMR spectrum of compound **2b** in $CDCl_3$ (100 MHz).

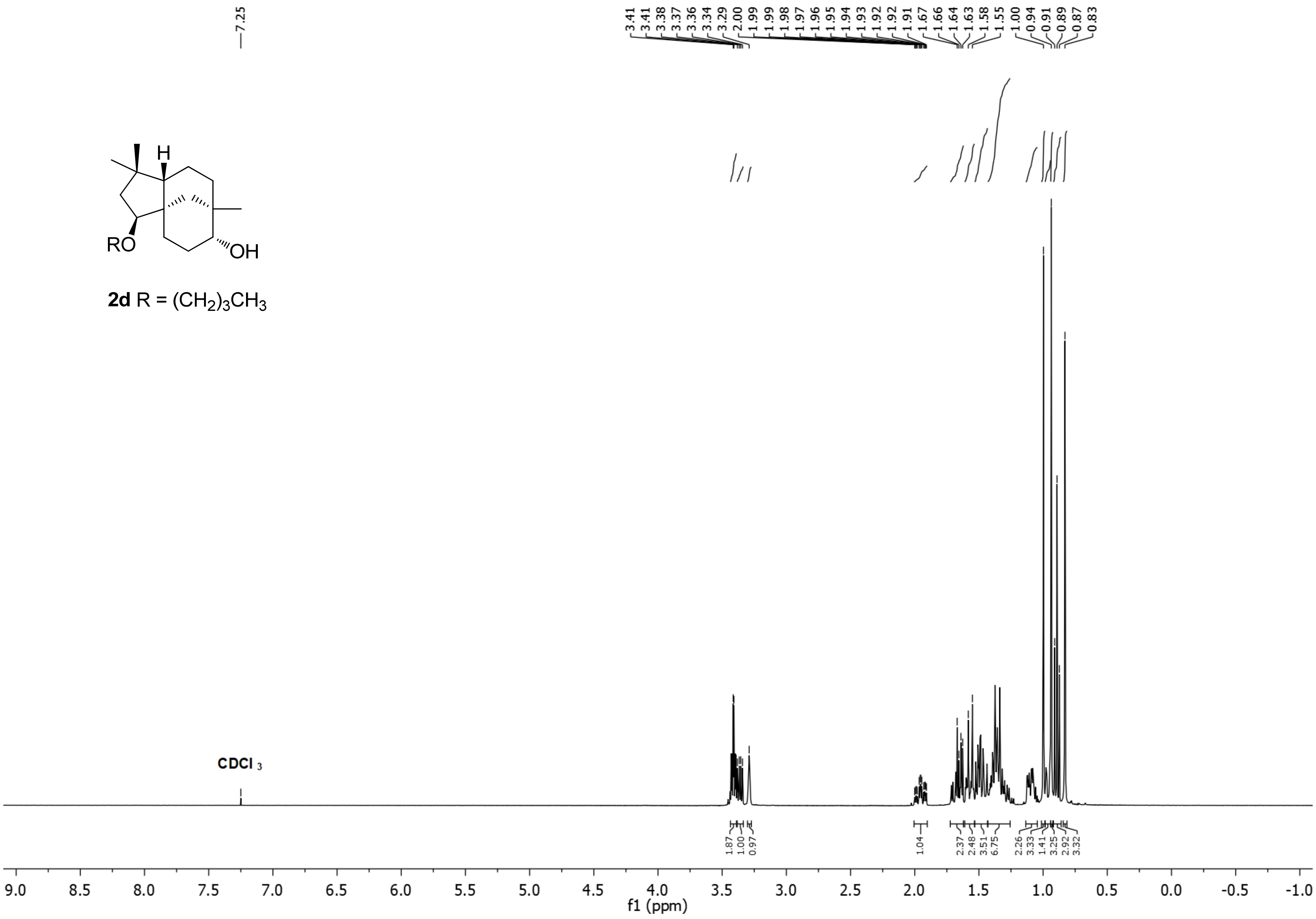

Figure S3. ^{1}H NMR spectrum of compound **2d** in $CDCl_3$ (400 MHz).

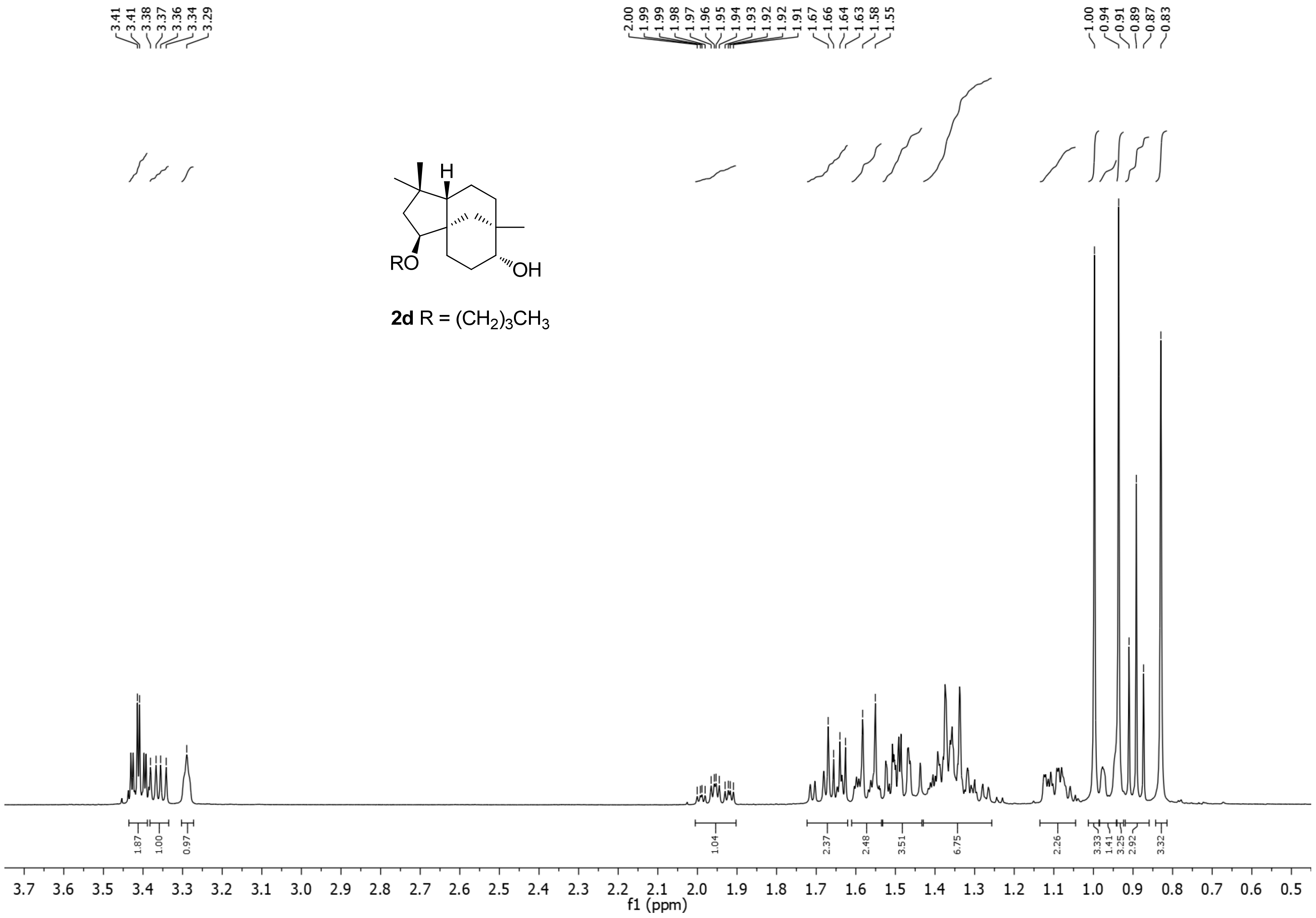

Figure S3a. Expansion (δ_H 3.7-0.5) of ^{1}H NMR spectrum of compound **2d** in $CDCl_3$ (400 MHz).

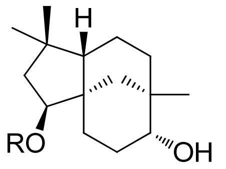

2d R = $(CH_2)_3CH_3$

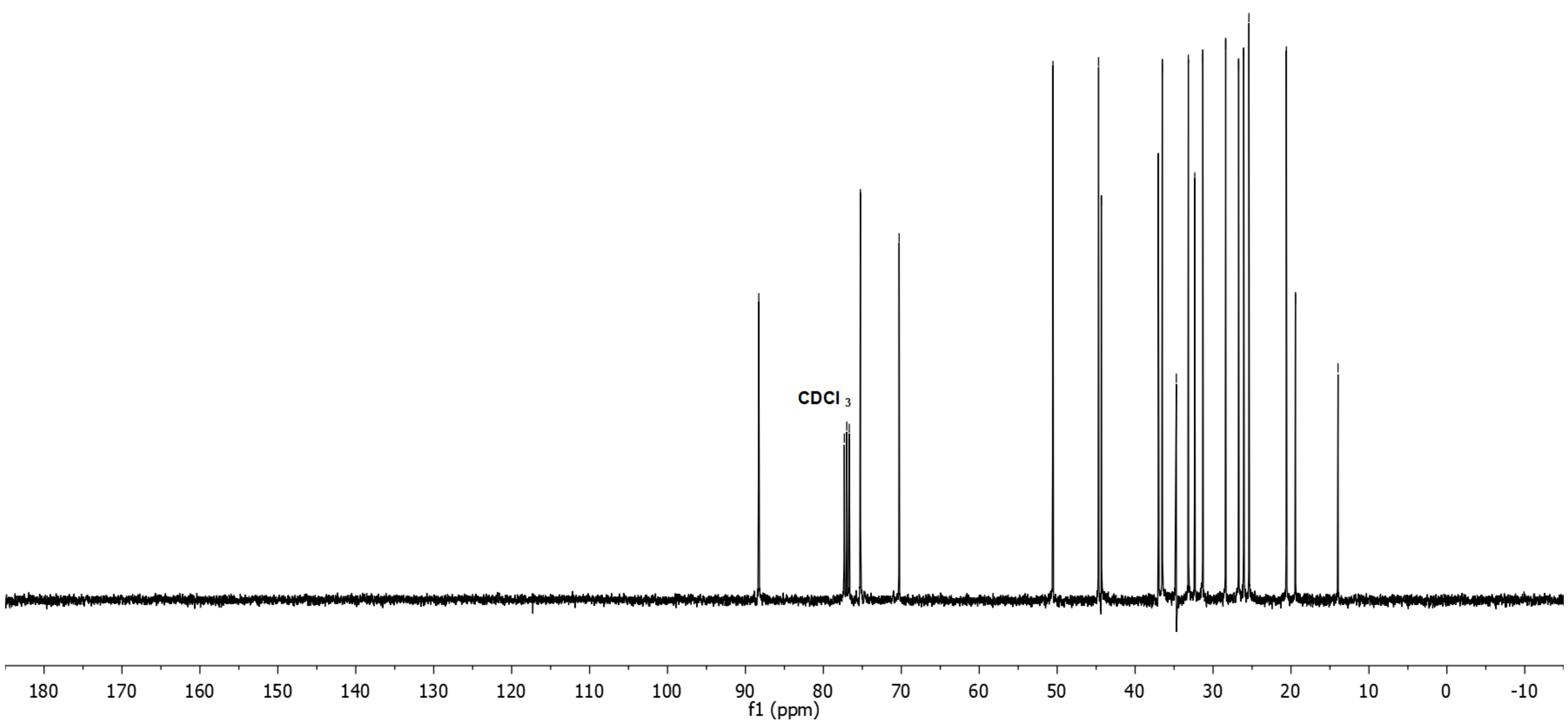

Figure S4. ^{13}C NMR spectrum of compound **2d** in $CDCl_3$ (100 MHz).

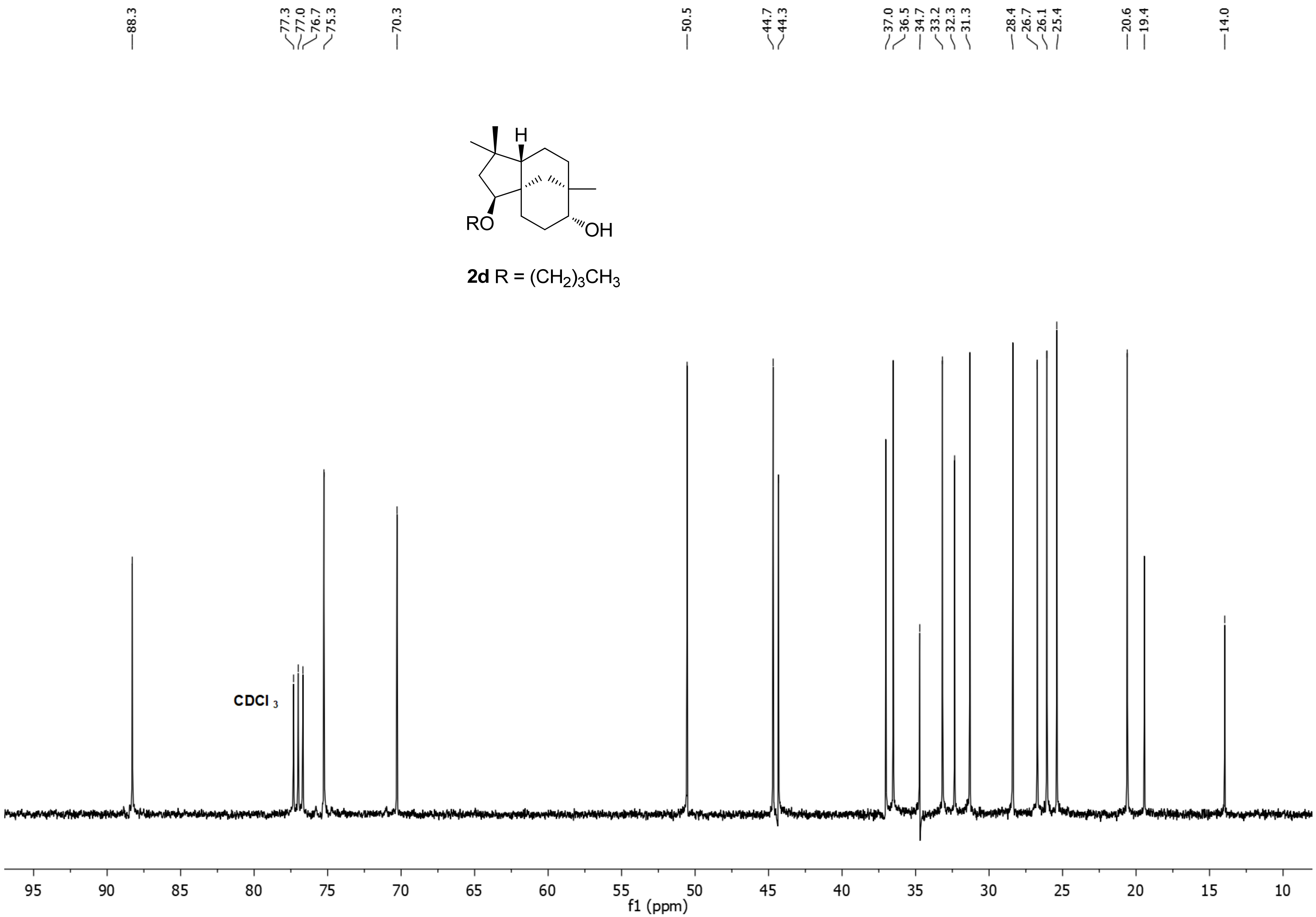

Figure S4a. Expansion (δ_C 95-10) of ^{13}C NMR spectrum of compound **2d** in $CDCl_3$ (100 MHz).

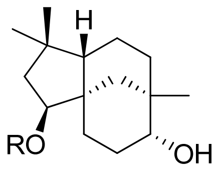

2f R = $(CH_2)_2Ph$

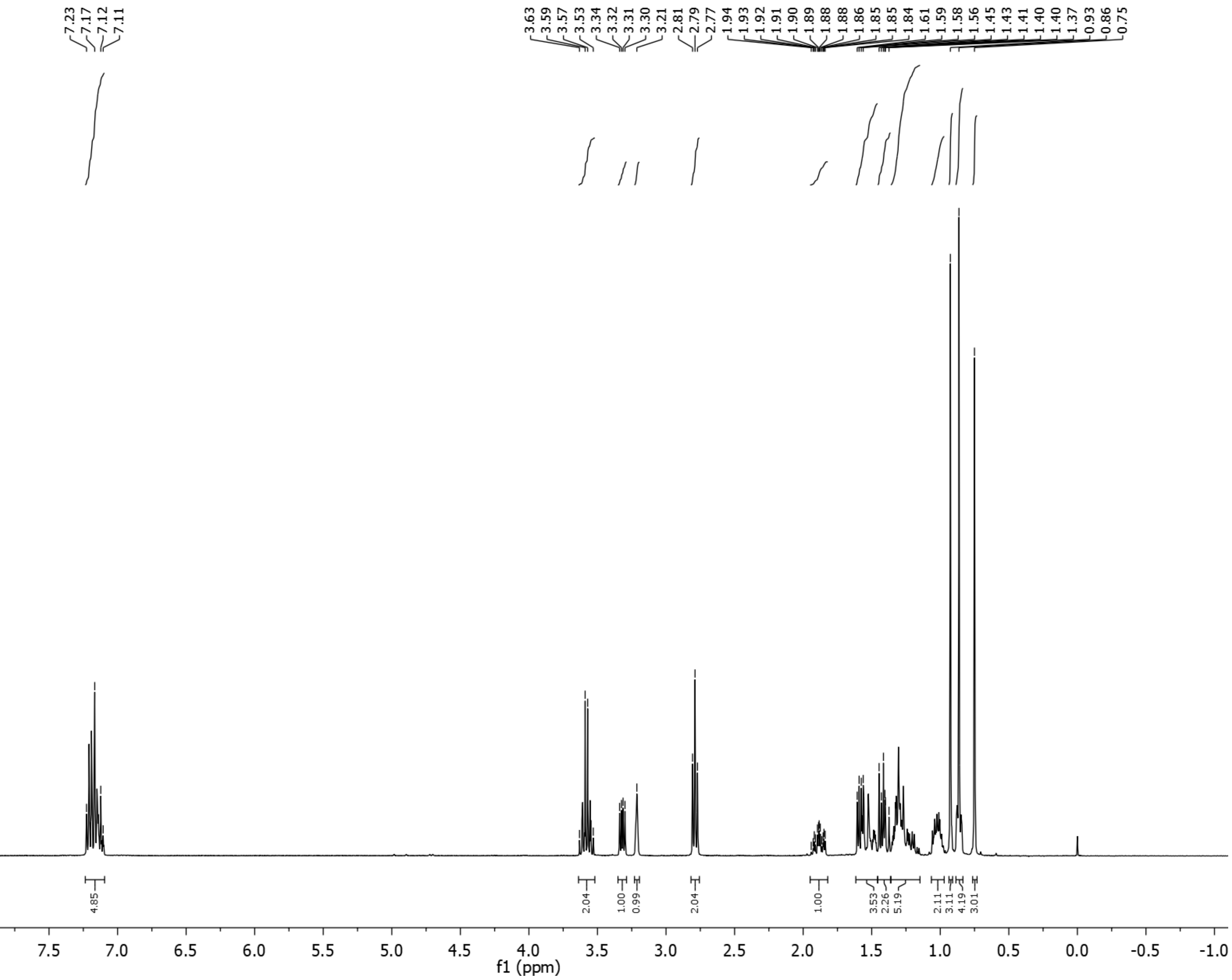

Figure S5. ^1H NMR spectrum of compound **2f** in $CDCl_3$ (400 MHz).

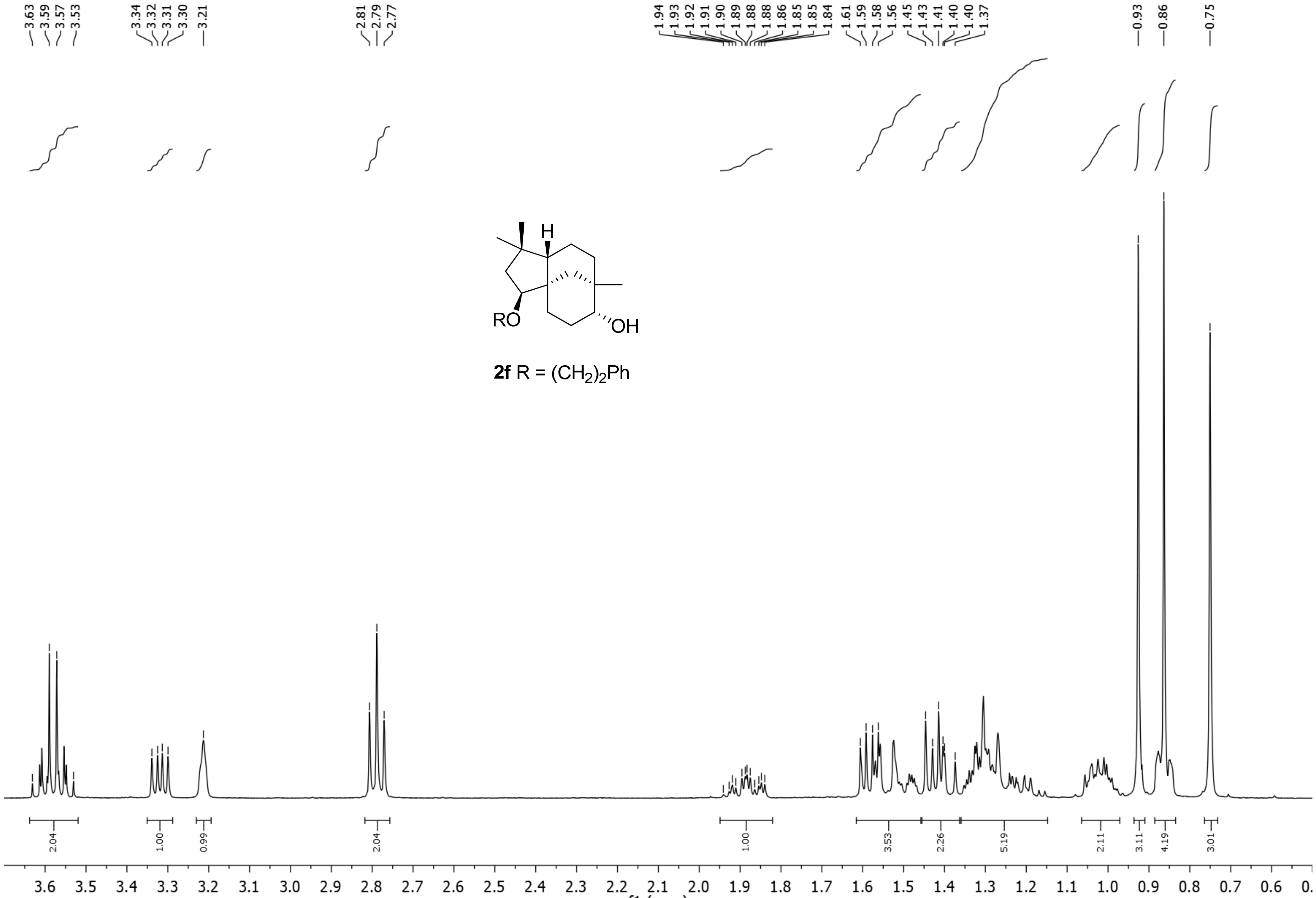

Figure S5a. Expansion (δ_H 3.7-0.5) of ^1H NMR spectrum of compound **2f** in $CDCl_3$ (400 MHz).

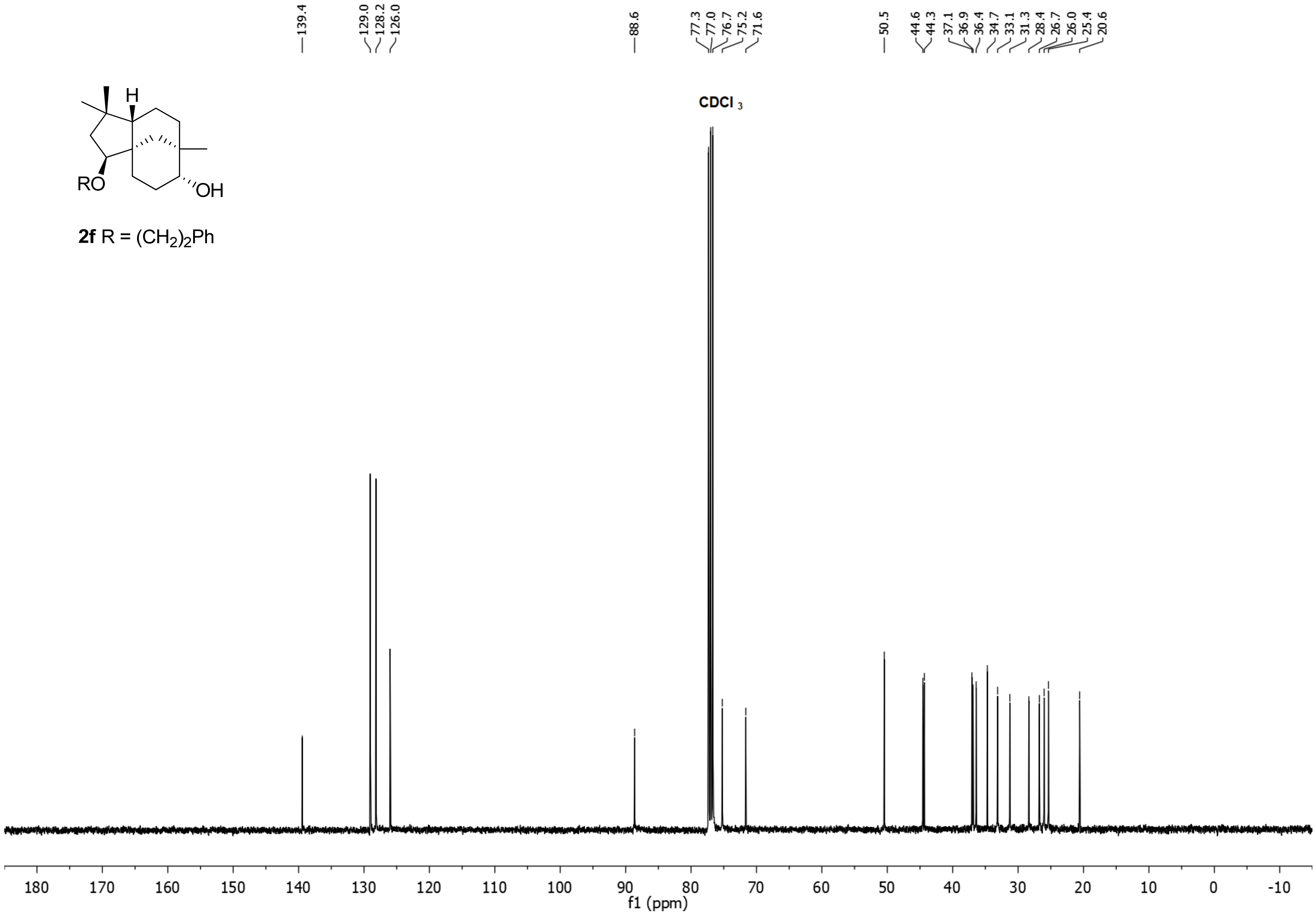

Figure S6. ^{13}C NMR spectrum of compound **2f** in $CDCl_3$ (100 MHz).

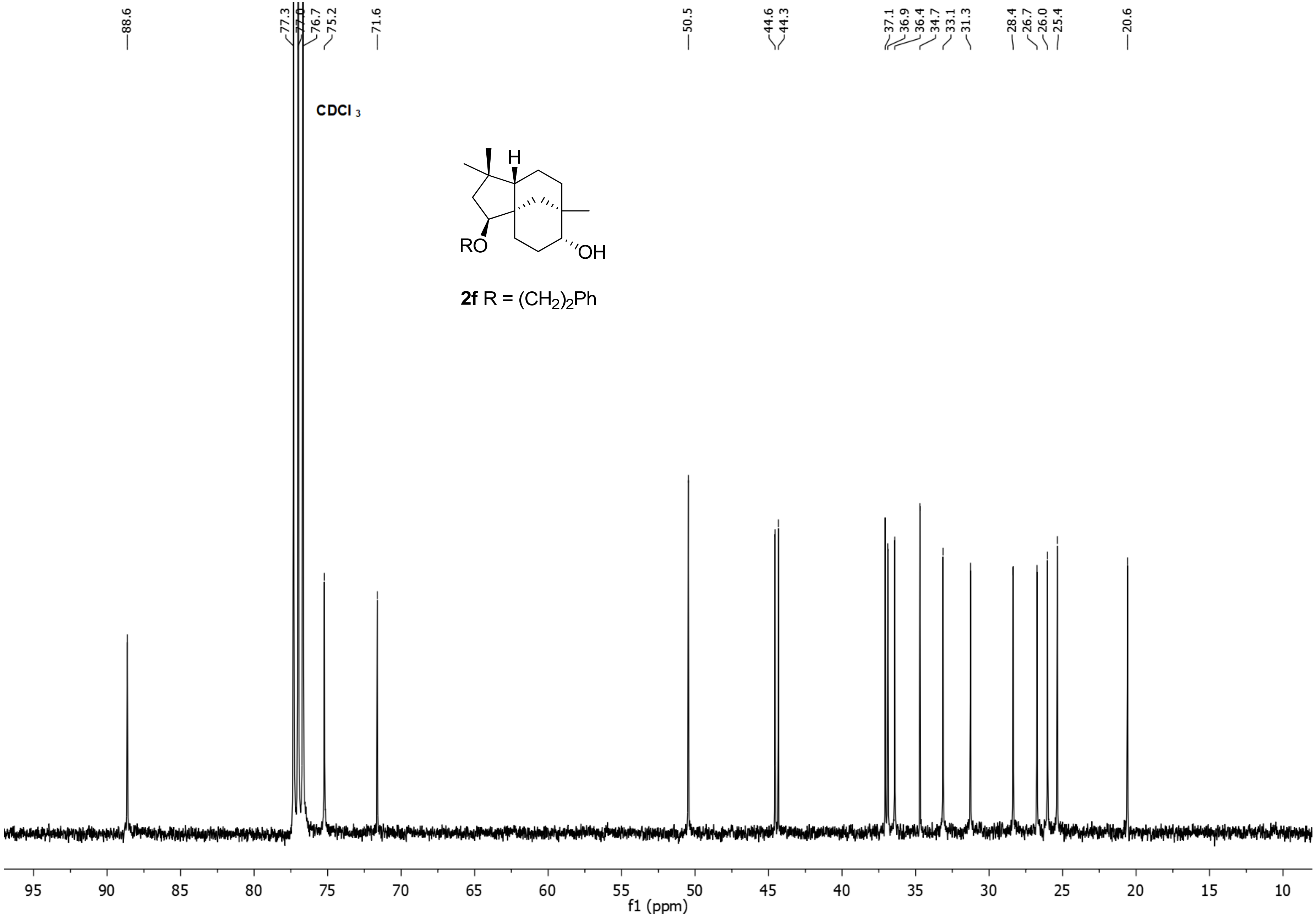

Figure S6a. Expansion (δ_C 95-10) of ^{13}C NMR spectrum of compound **2f** in $CDCl_3$ (100 MHz).

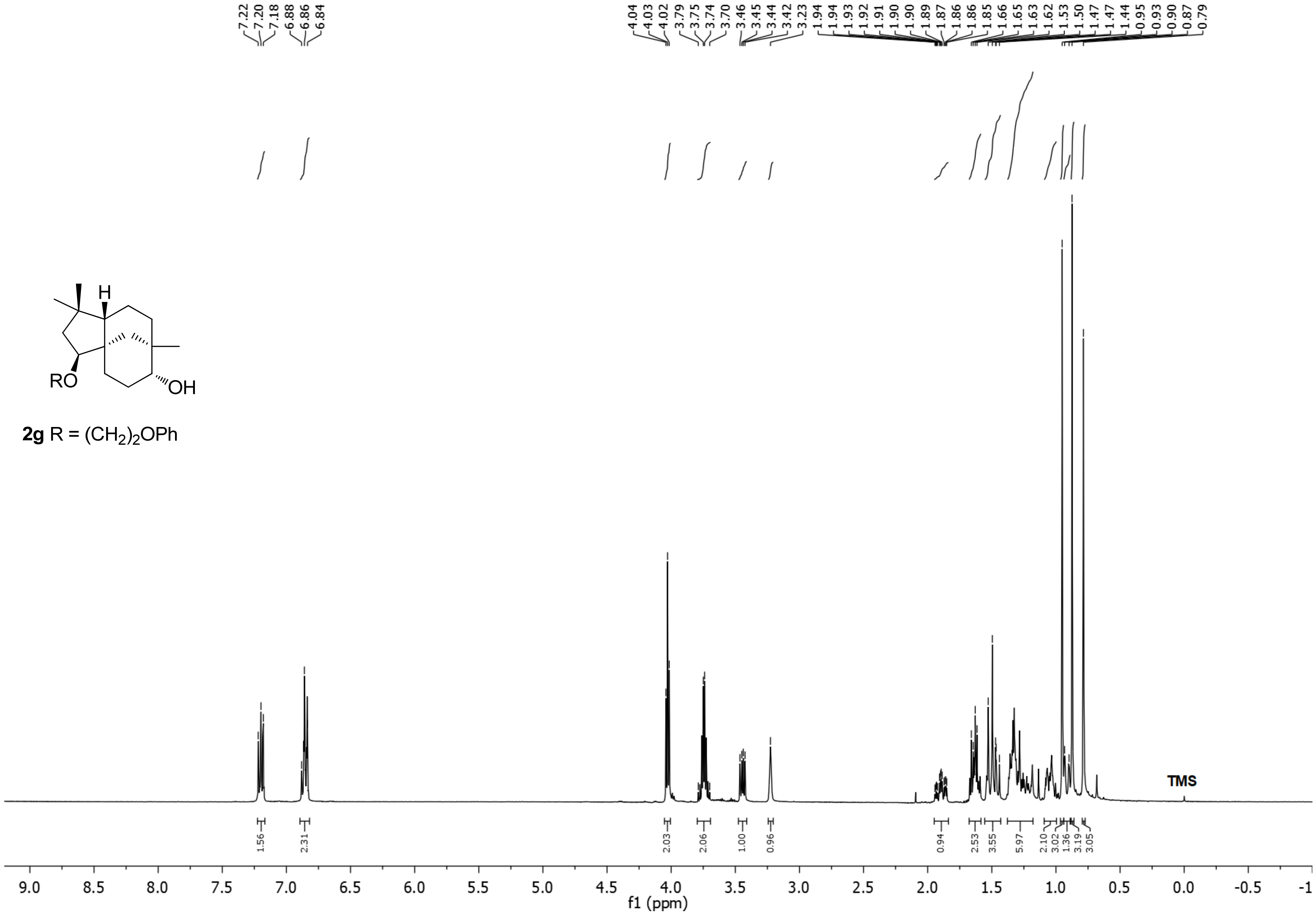

Figure S7. ^{1}H NMR spectrum of compound **2g** in $CDCl_3$ (400 MHz).

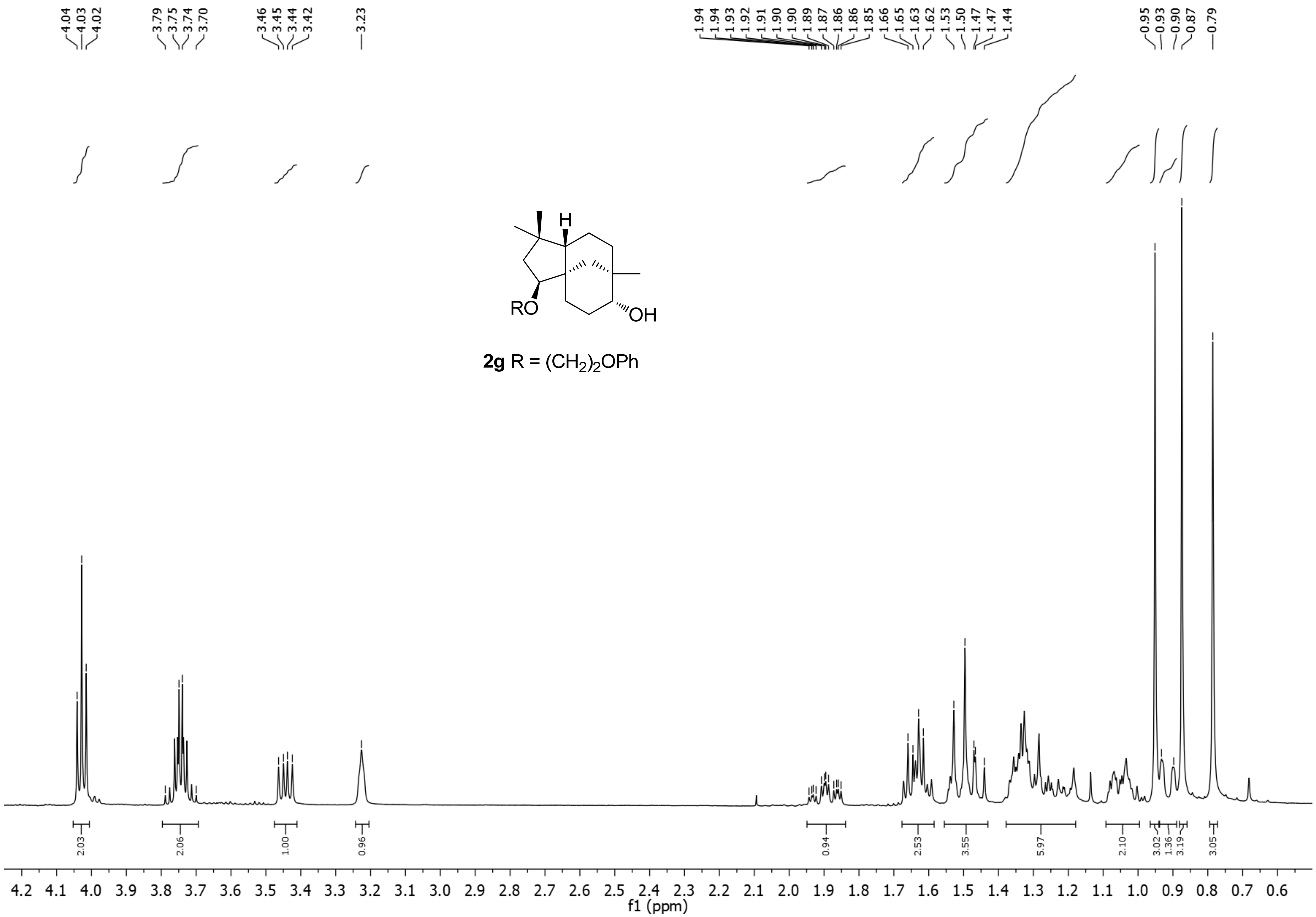

Figure S7a. Expansion (δ_H 4.2-0.5) of ^{1}H NMR spectrum of compound **2g** in $CDCl_3$ (400 MHz).

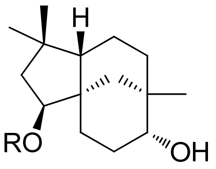

2g R = $(CH_2)_2OPh$

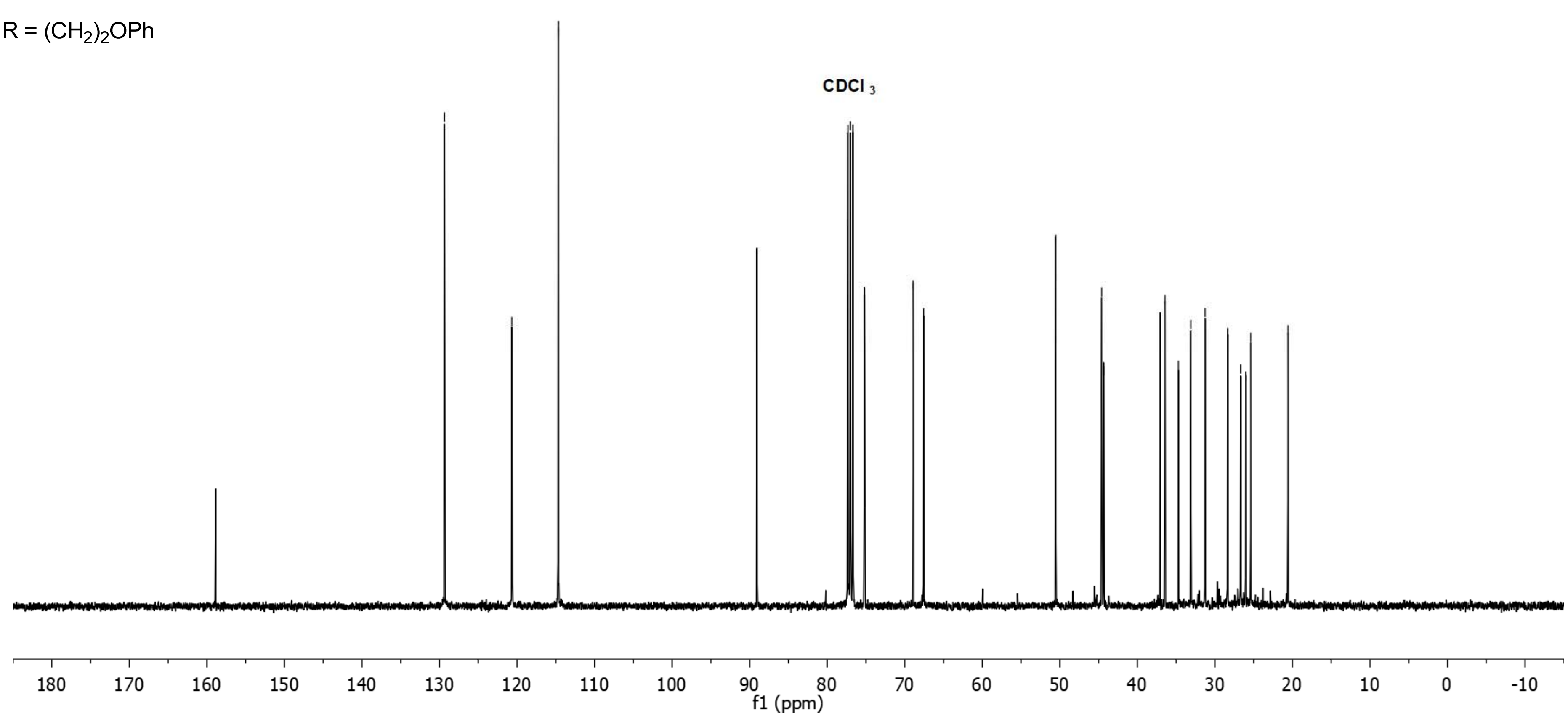

Figure S8. ^{13}C NMR spectrum of compound **2g** in $CDCl_3$ (100 MHz).

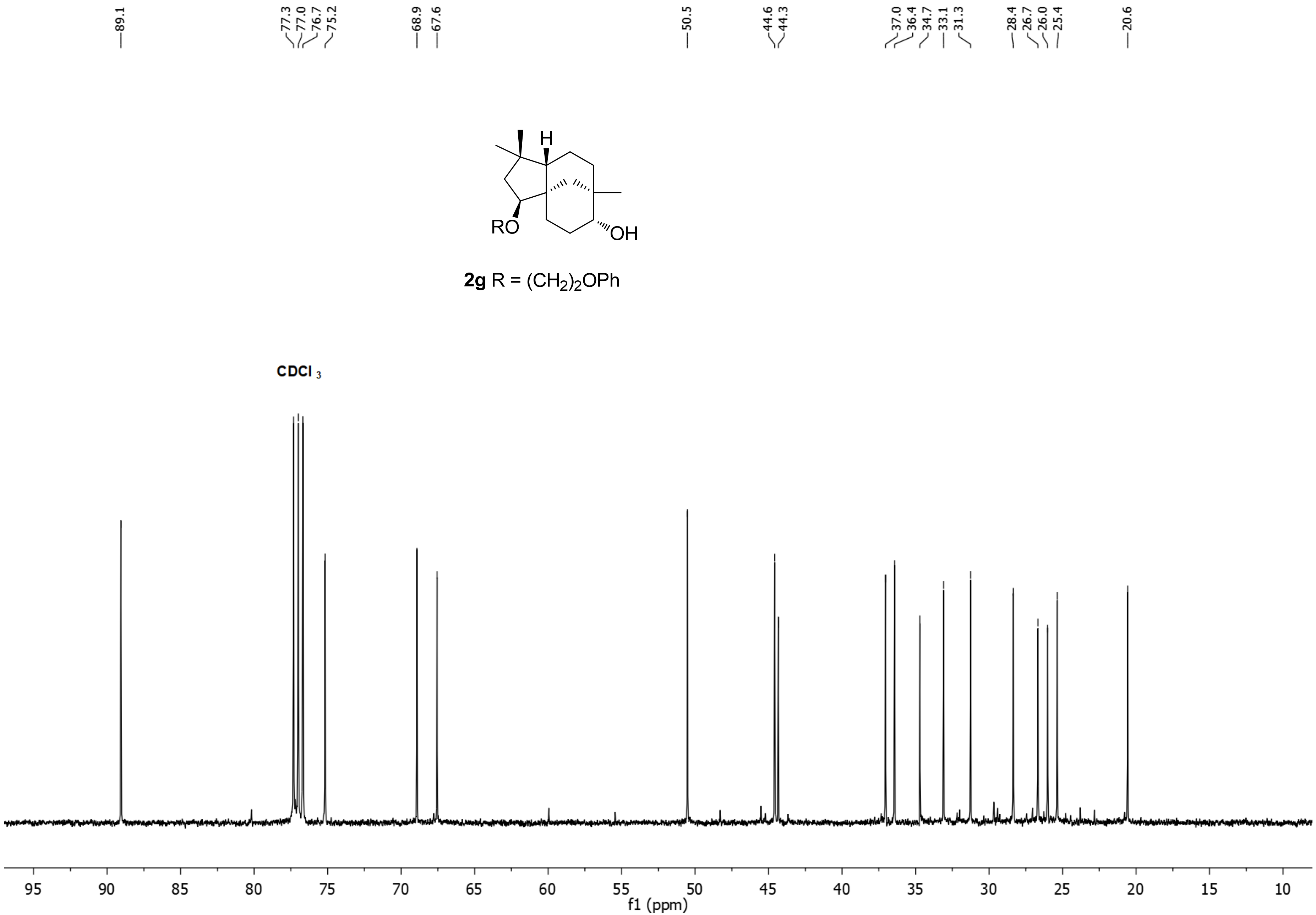

Figure S8a. Expansion (δ_C 95-10) of ^{13}C NMR spectrum of compound **2g** in $CDCl_3$ (100 MHz).

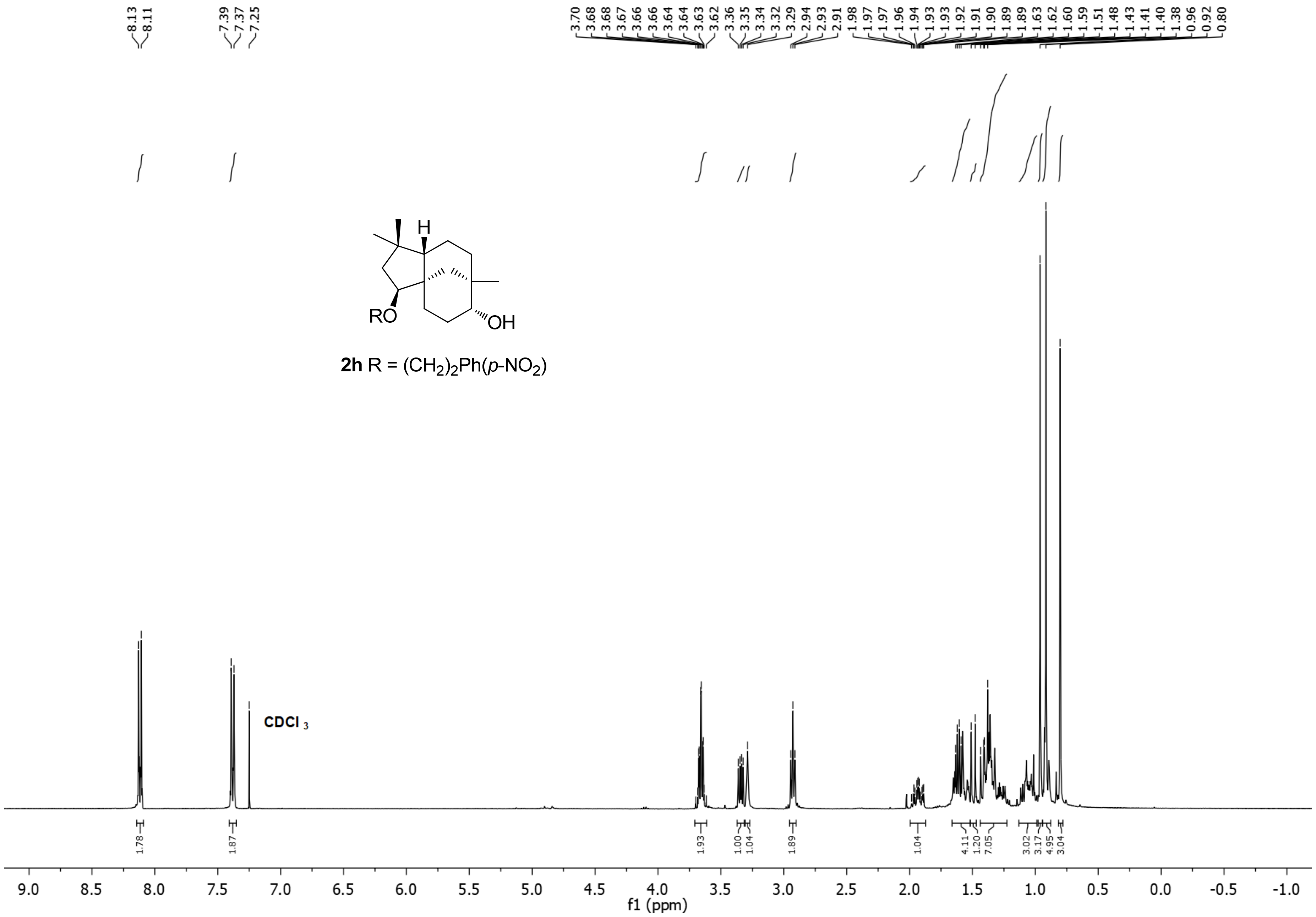

Figure S9. ^{1}H NMR spectrum of compound **2h** in $CDCl_3$ (400 MHz).

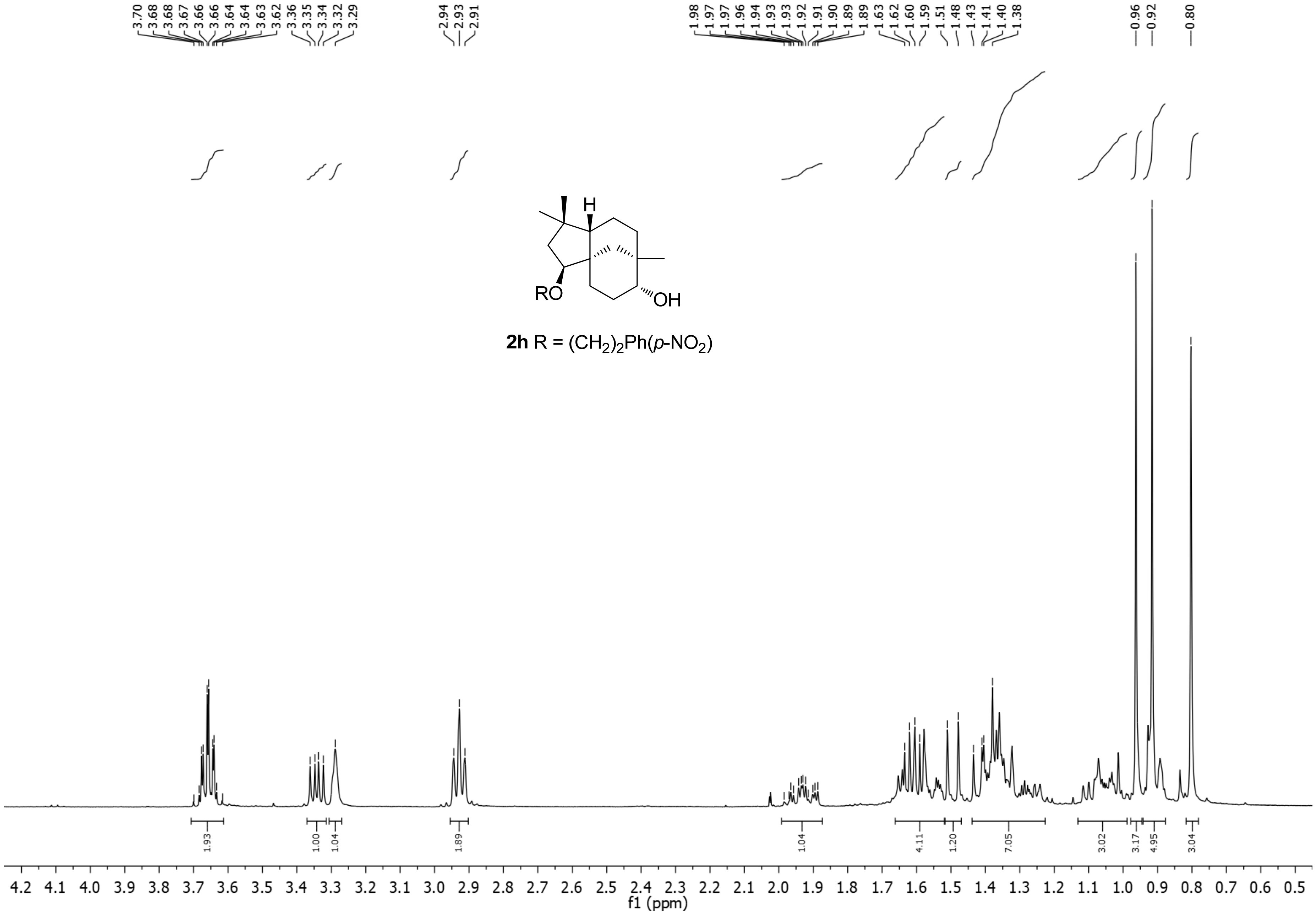

Figure S9a. Expansion (δ_H 4.2-0.5) of ^{1}H NMR spectrum of compound **2h** in $CDCl_3$ (400 MHz).

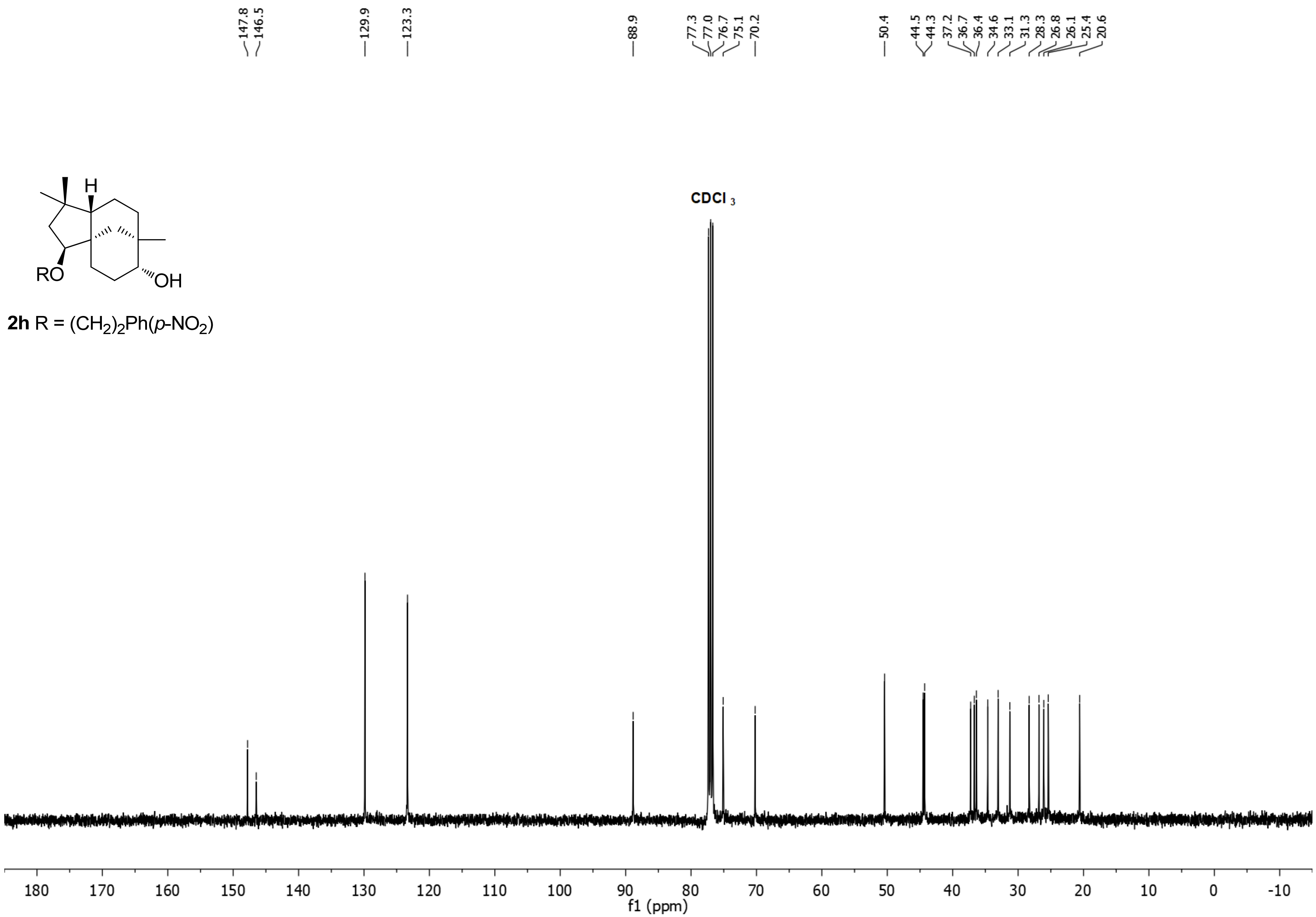

Figure S10. ^{13}C NMR spectrum of compound **2h** in $CDCl_3$ (100 MHz).

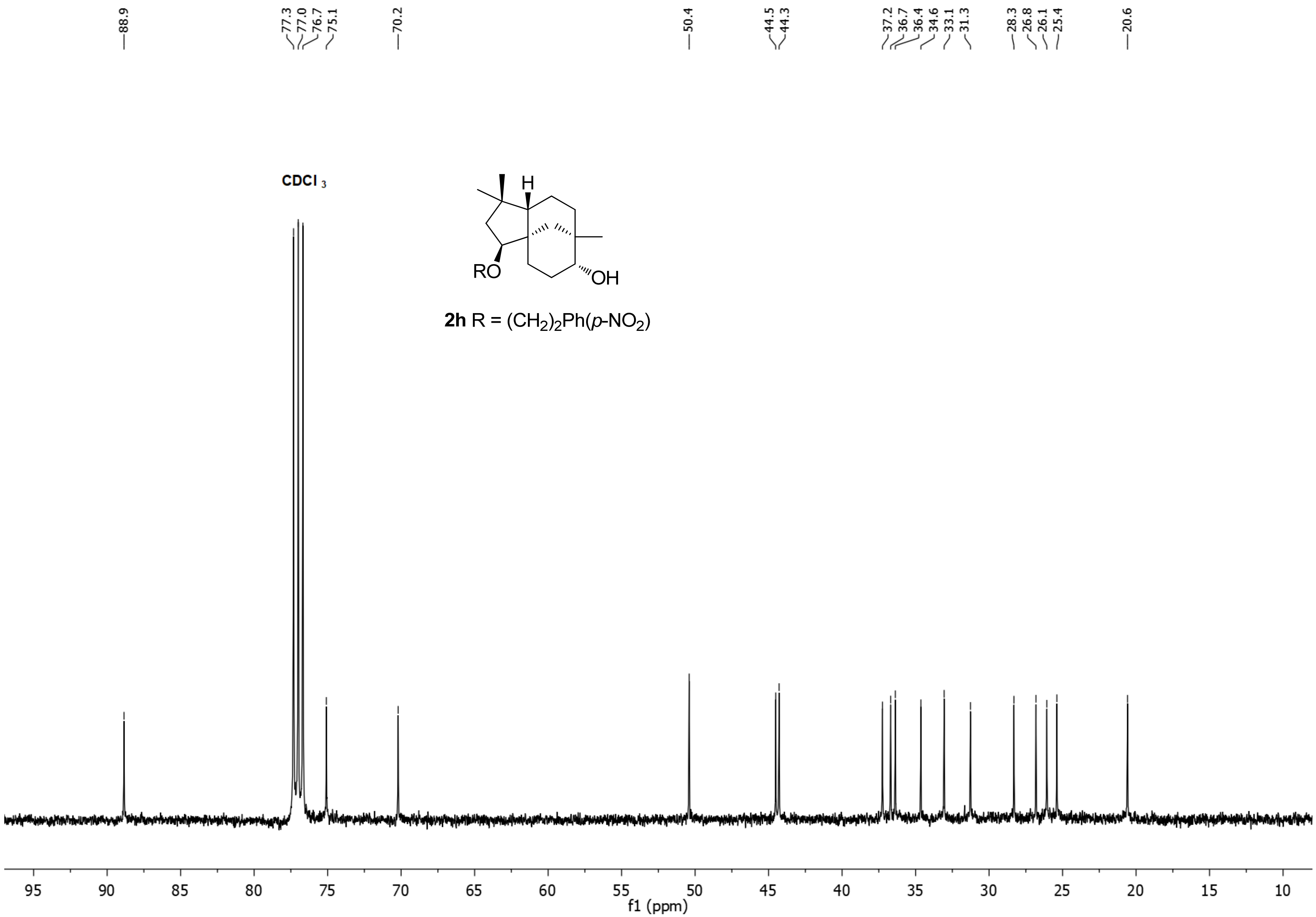

Figure S10a. Expansion (δ_C 95-10) of ^{13}C NMR spectrum of compound **2h** in $CDCl_3$ (100 MHz).

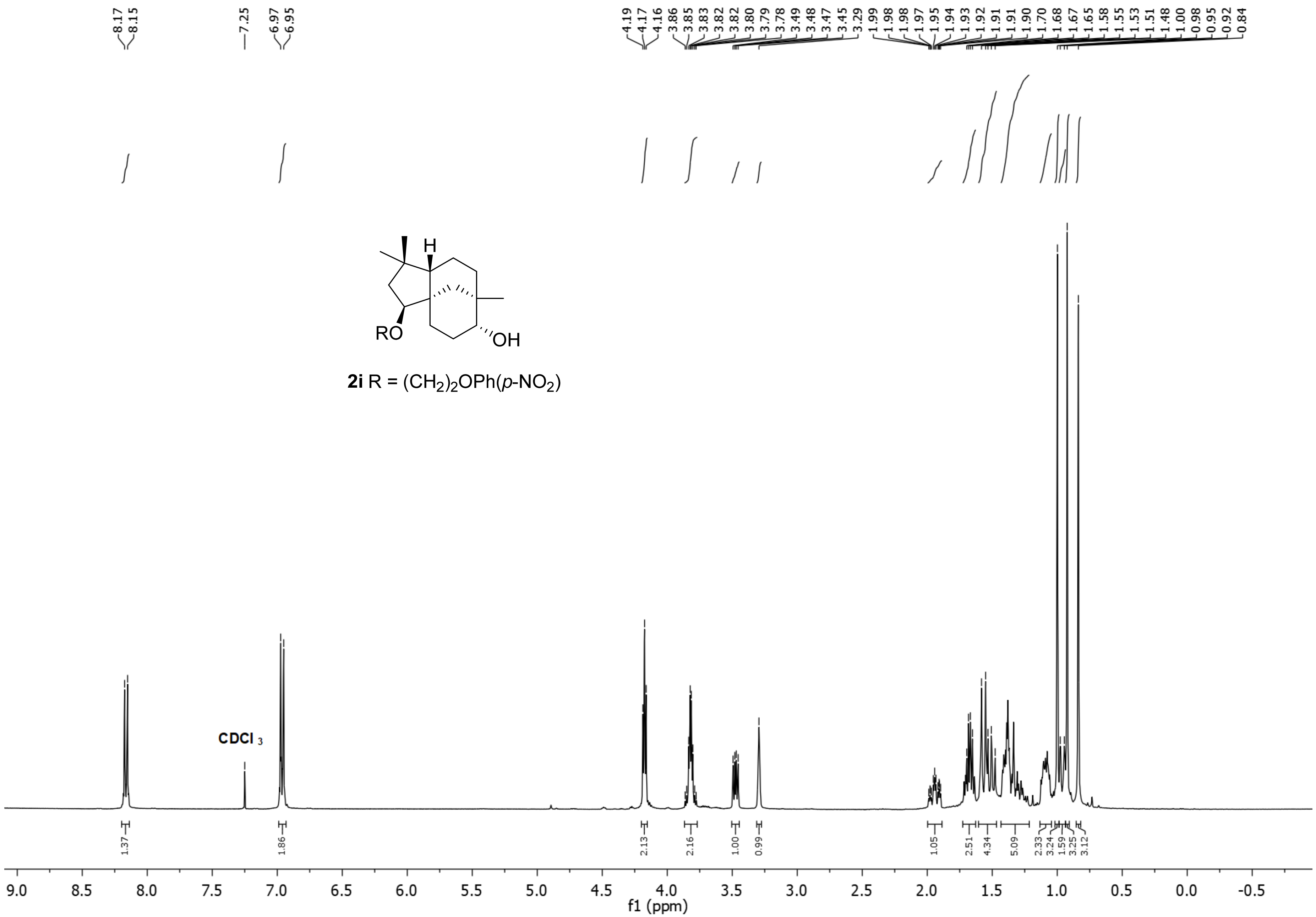

Figure S11. ^{1}H NMR spectrum of compound **2i** in $CDCl_3$ (400 MHz).

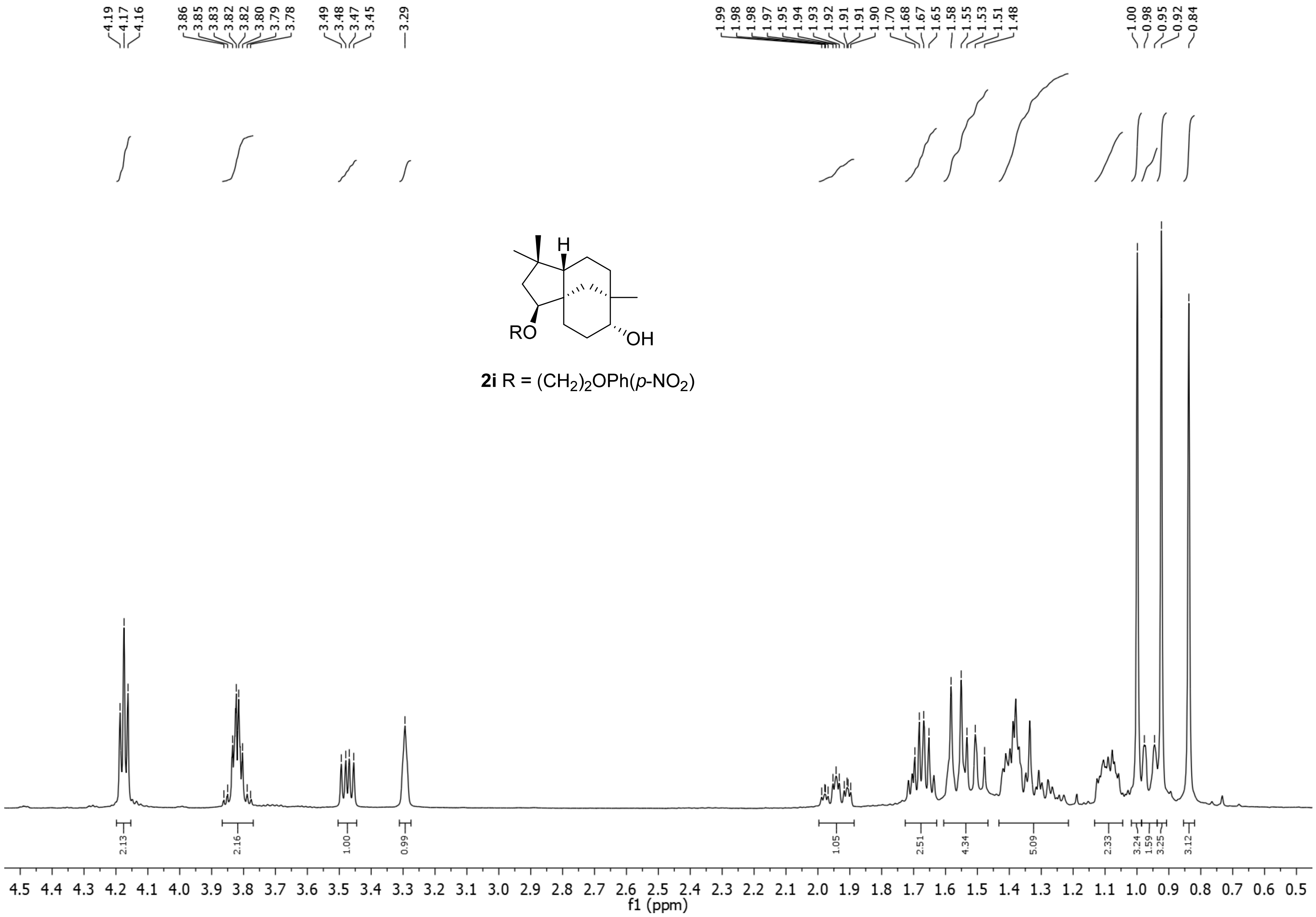

Figure S11a. Expansion (δ_H 4.5-0.5) of ^{1}H NMR spectrum of compound **2i** in $CDCl_3$ (400 MHz).

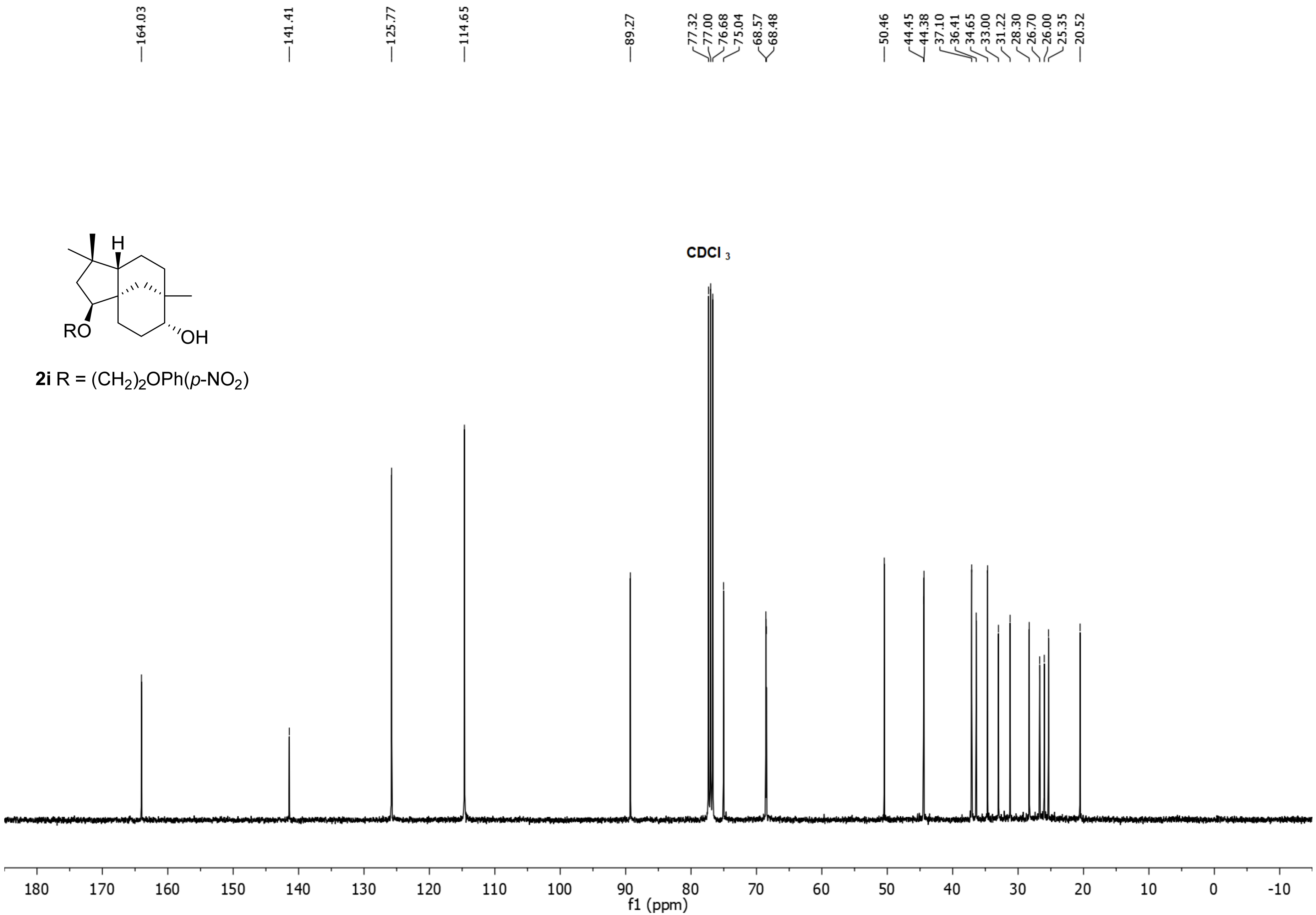

Figure S12. ^{13}C NMR spectrum of compound **2i** in $CDCl_3$ (100 MHz).

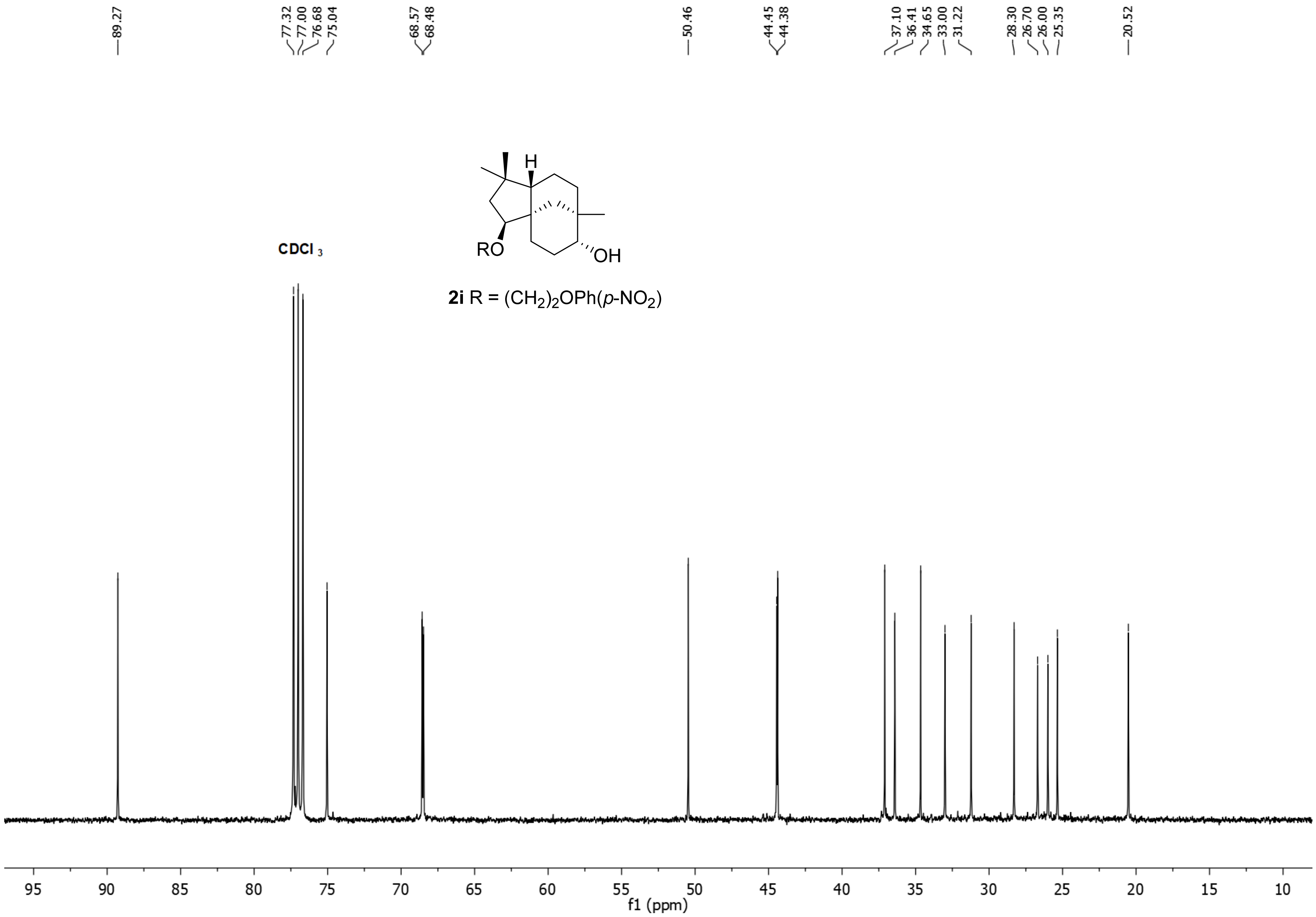

Figure S12a. Expansion (δ_C 95-10) of ^{13}C NMR spectrum of compound **2i** in $CDCl_3$ (100 MHz).

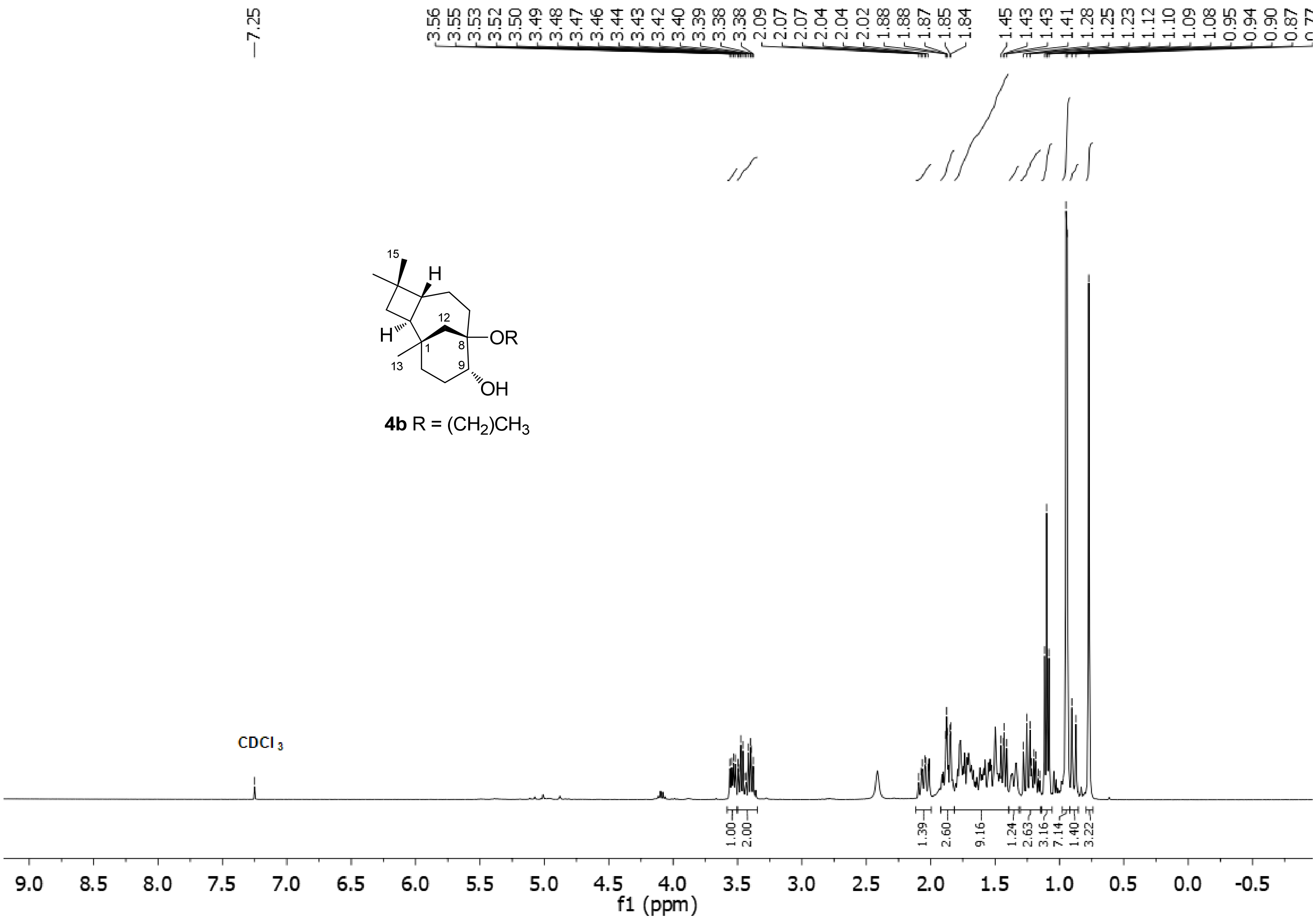

Figure S13. ^{1}H NMR spectrum of compound **4b** in $CDCl_3$ (400 MHz).

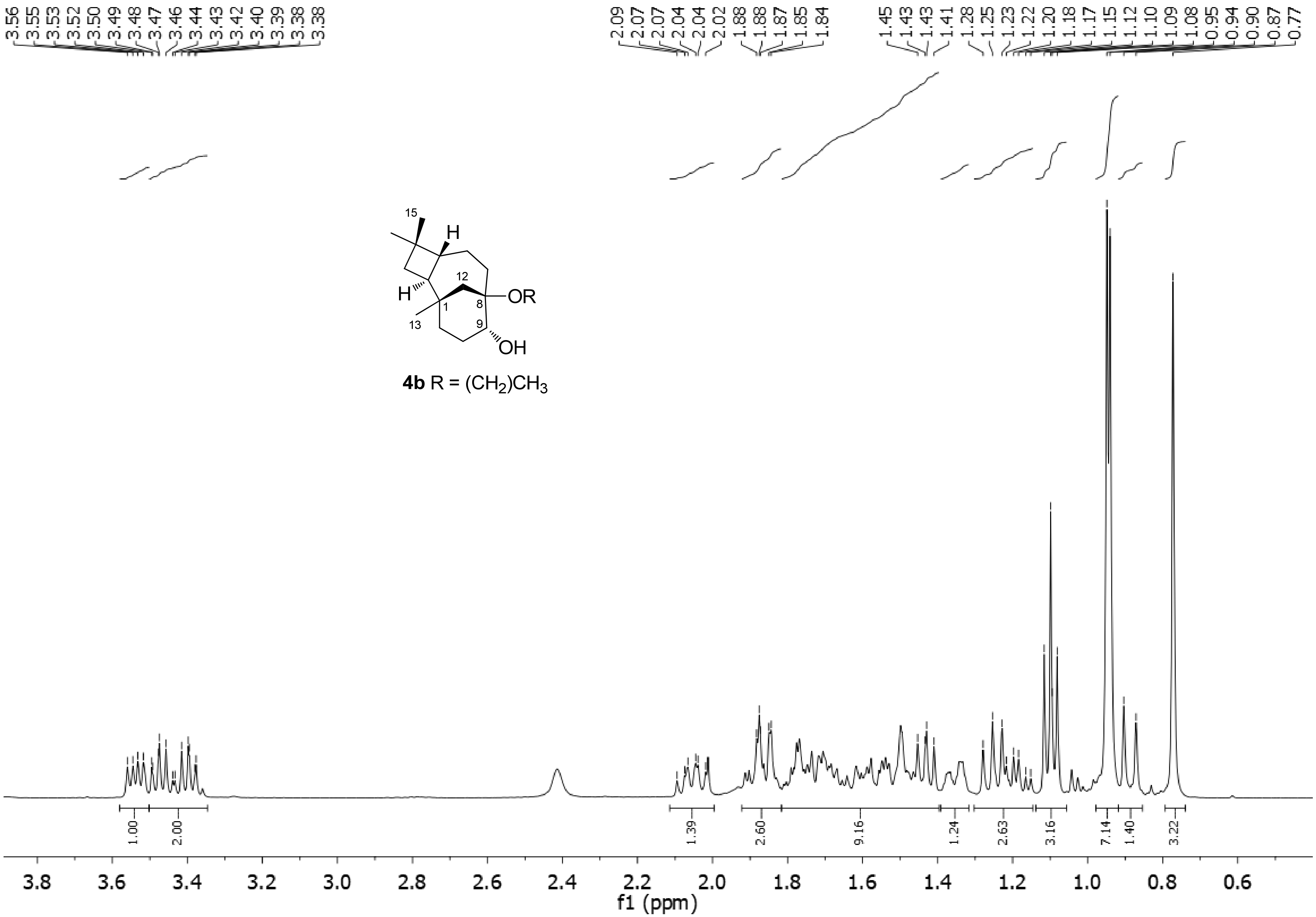

Figure S13a. Expansion (δ_H 3.8-0.5) of ^{1}H NMR spectrum of compound **4b** in $CDCl_3$ (400 MHz).

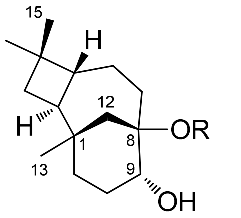

4b R = $(CH_2)CH_3$

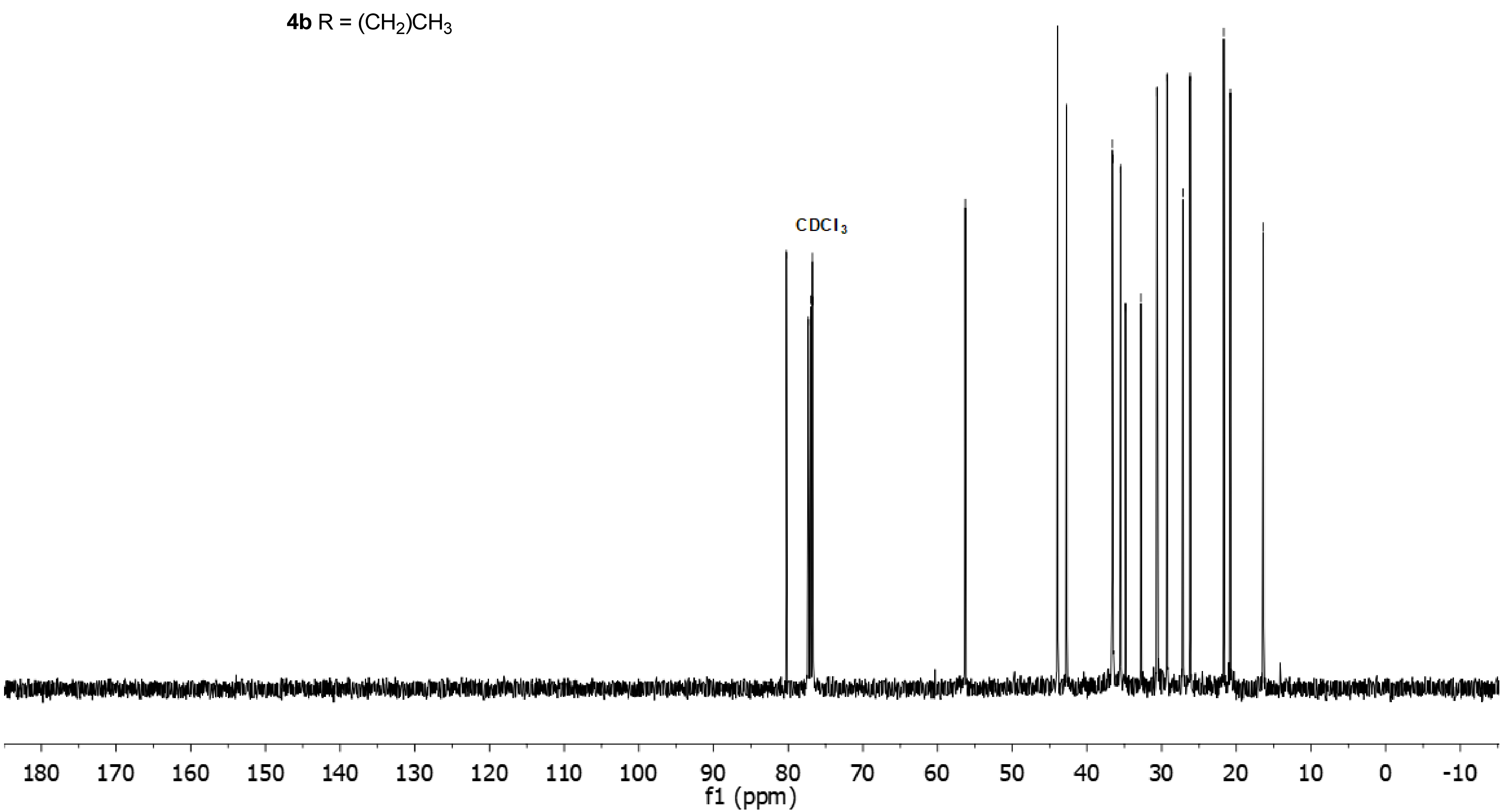

Figure S14. ^{13}C NMR spectrum of compound **4b** in $CDCl_3$ (100 MHz).

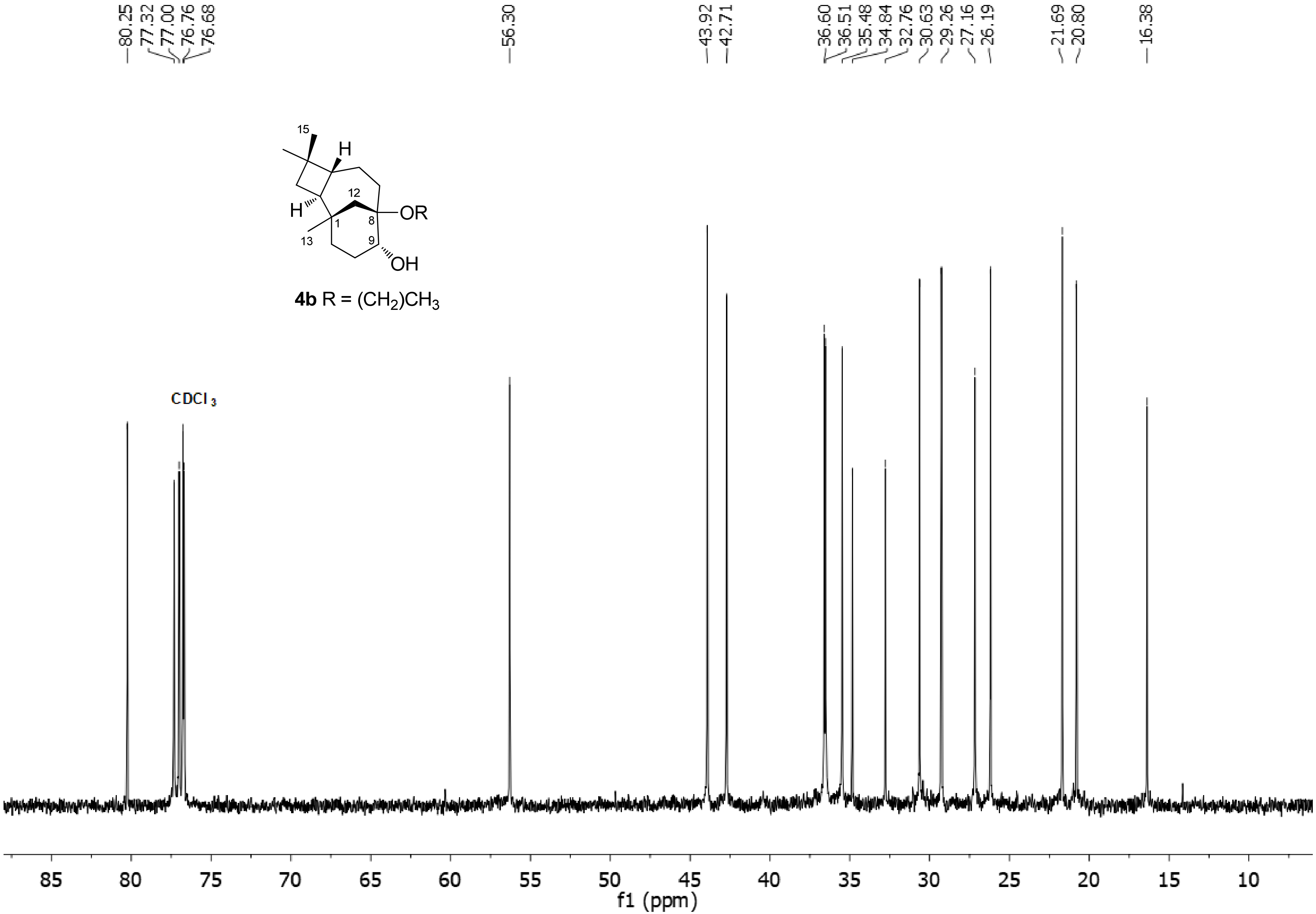

Figure S14a. Expansion (δ_C 95-10) of ^{13}C NMR spectrum of compound **4b** in $CDCl_3$ (100 MHz).

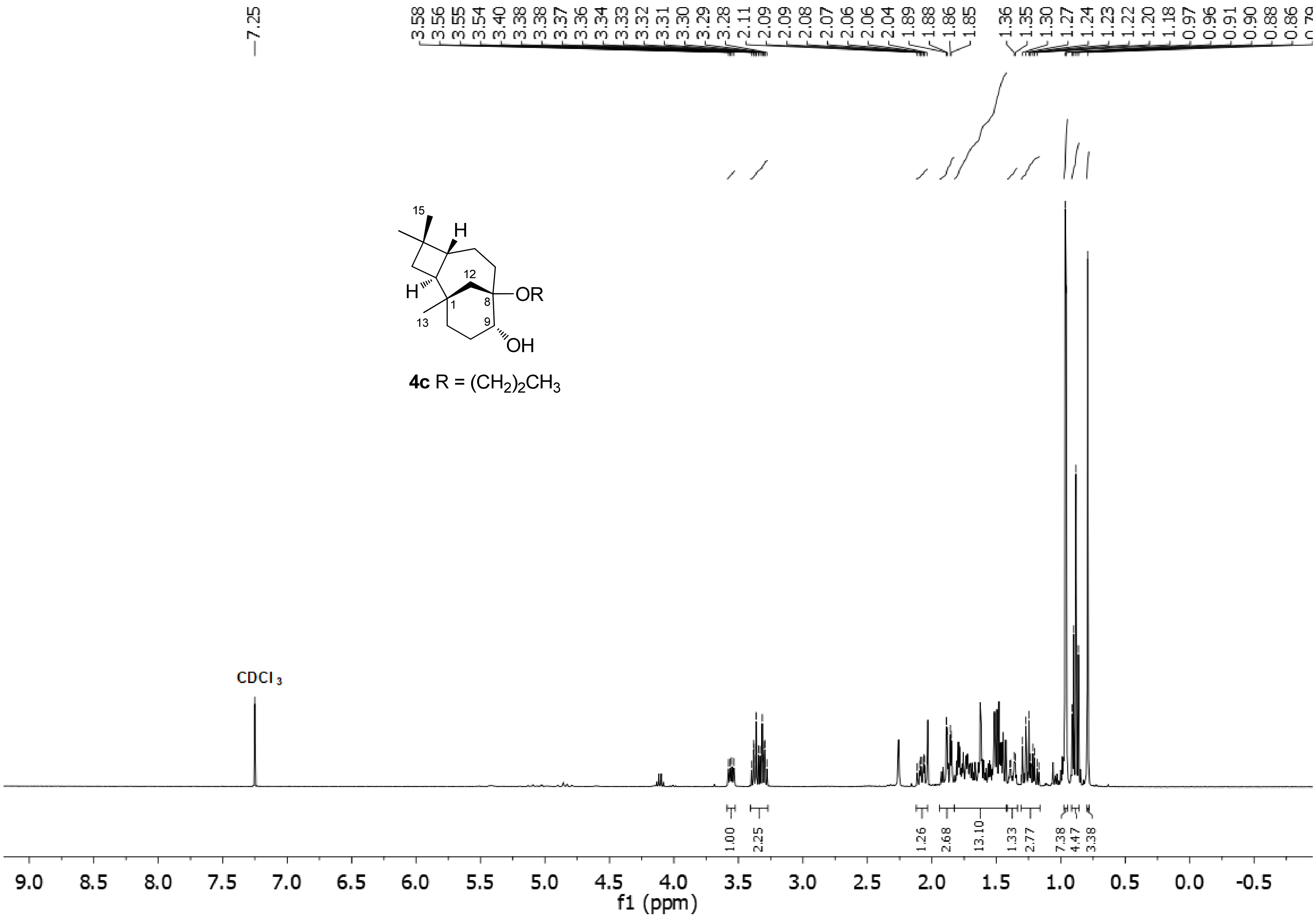

Figure S15. ^{1}H NMR spectrum of compound **4c** in $CDCl_3$ (400 MHz).

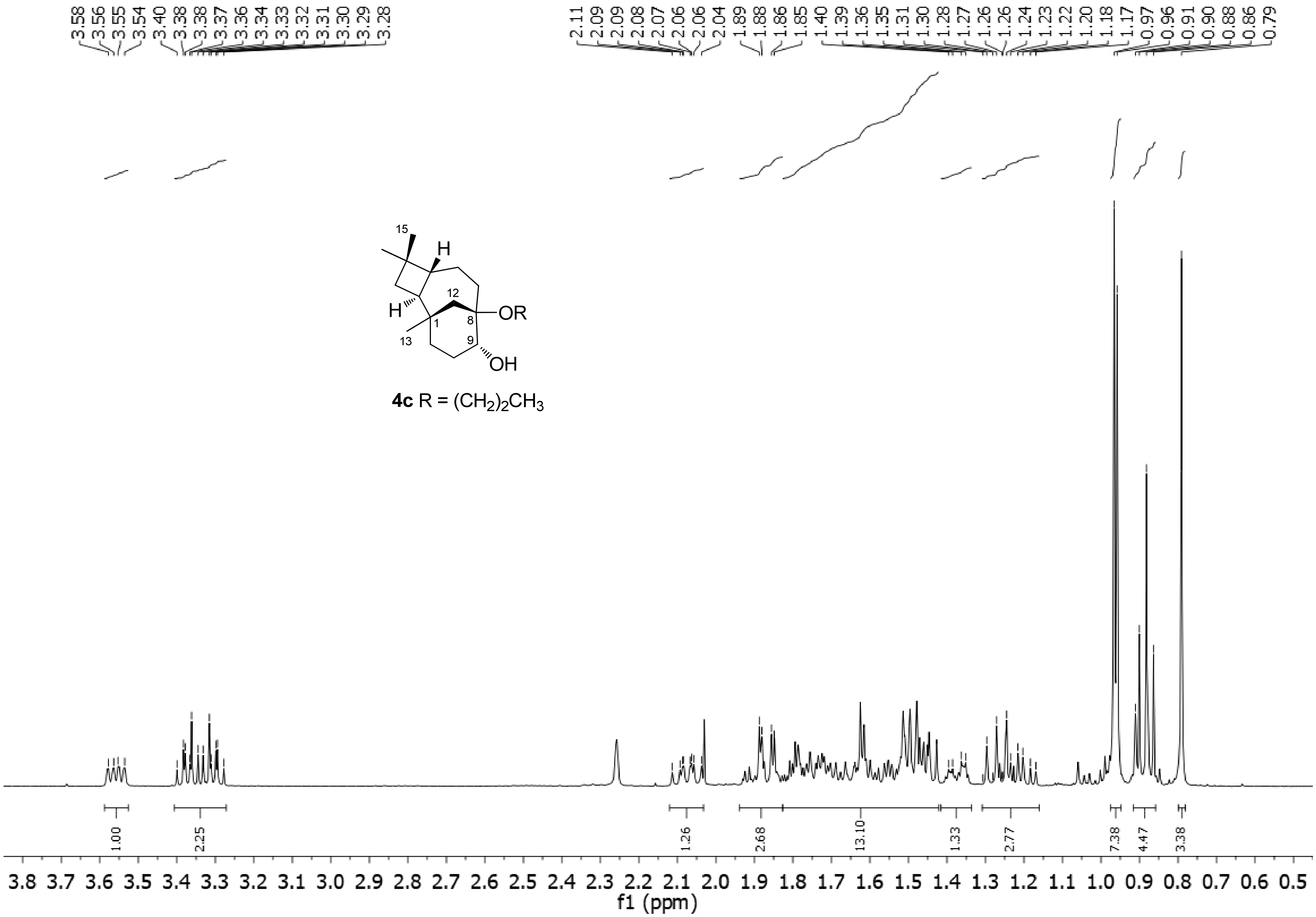

Figure S15a. Expansion (δ_H 3.8-0.5) of ^{1}H NMR spectrum of compound **4c** in $CDCl_3$ (400 MHz).

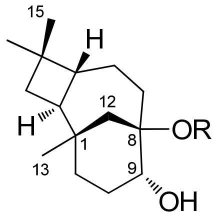

4c R = $(CH_2)_2CH_3$

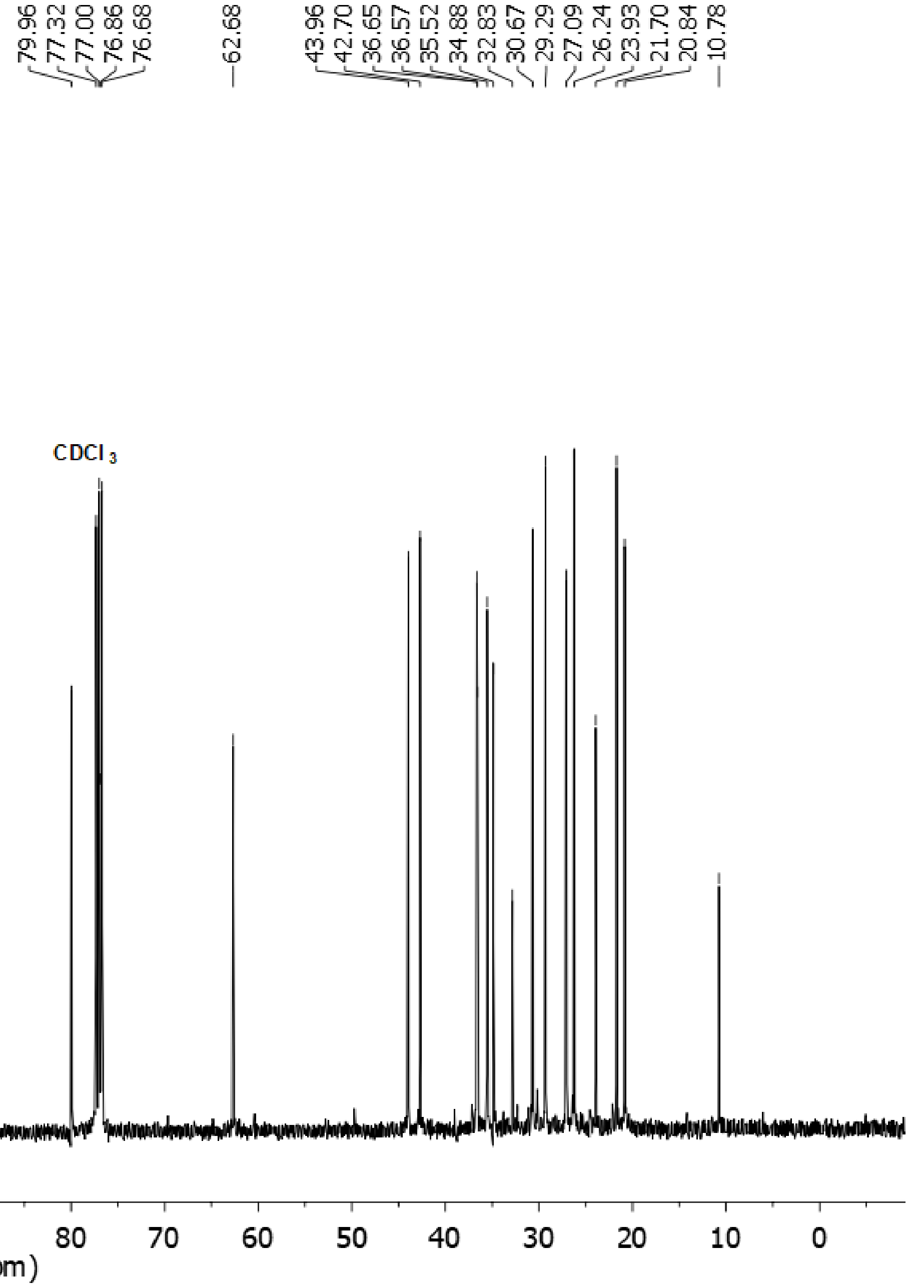

Figure S16. ^{13}C NMR spectrum of compound **4c** in $CDCl_3$ (100 MHz).

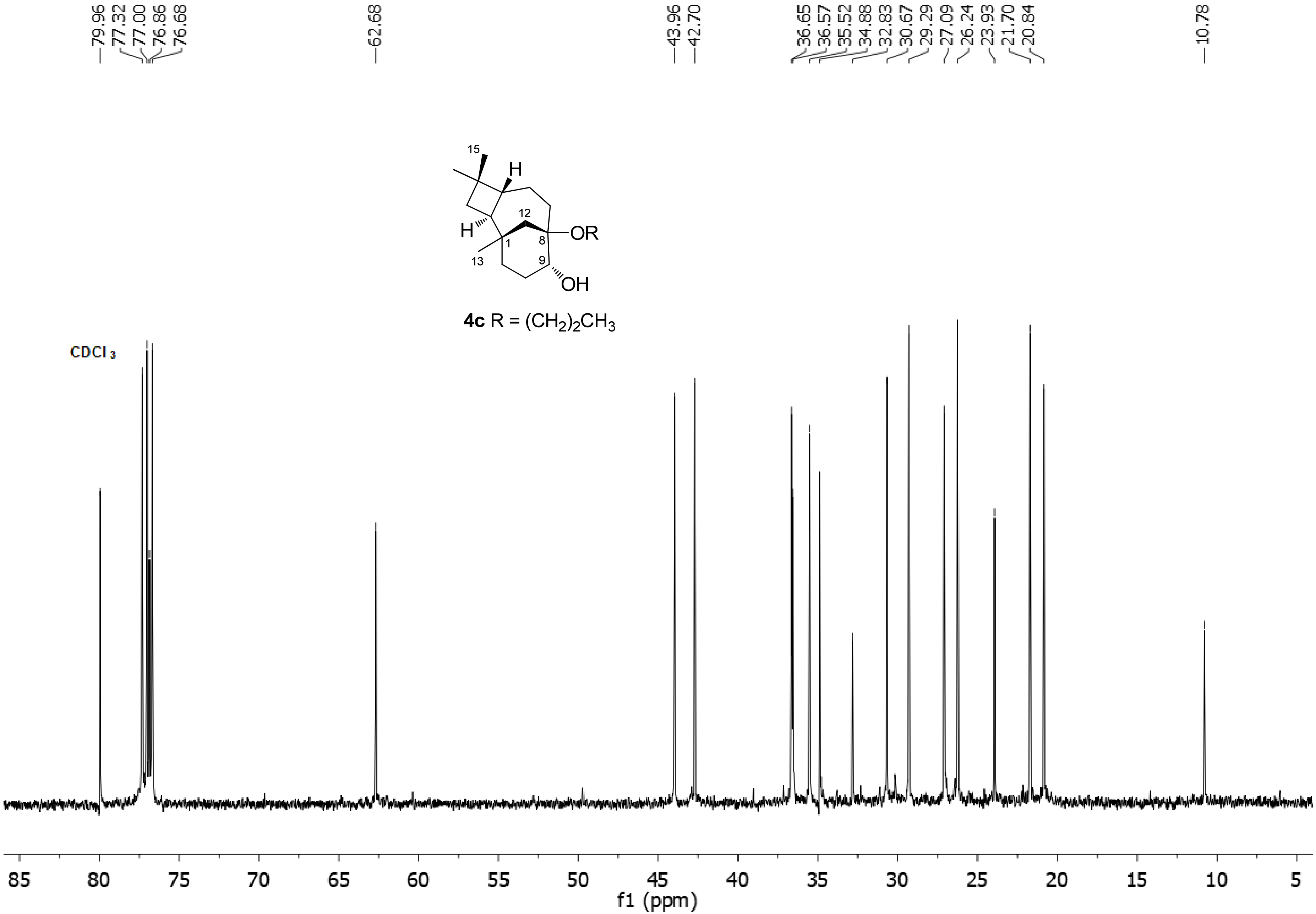

Figure S16a. Expansion (δ_C 85-5) of ^{13}C NMR spectrum of compound **4c** in $CDCl_3$ (100 MHz).

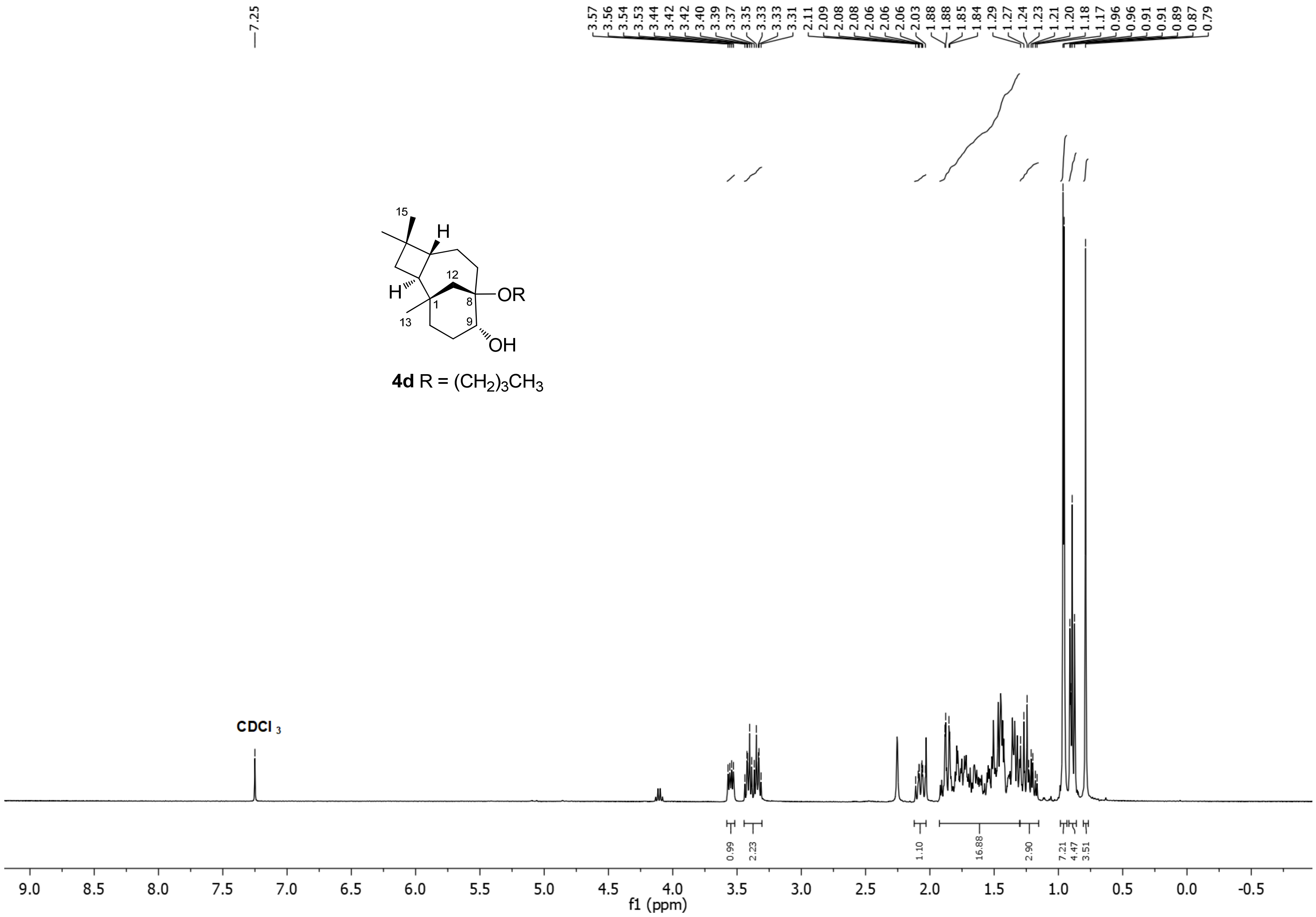

Figure S17. ^{1}H NMR spectrum of compound **4d** in $CDCl_3$ (400 MHz).

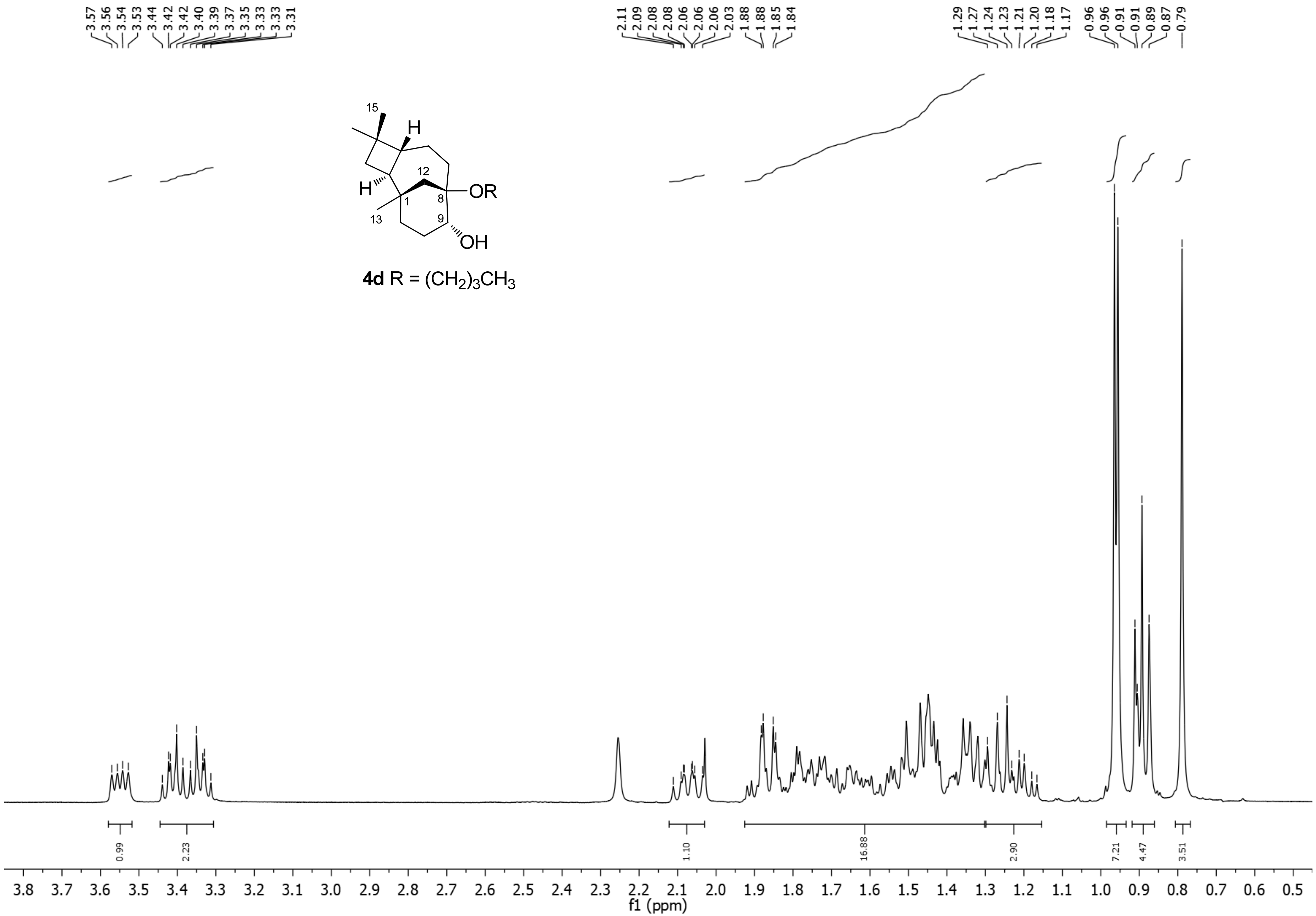

Figure S17a. Expansion (δ_H 3.8-0.5) of ^{1}H NMR spectrum of compound **4d** in $CDCl_3$ (400 MHz).

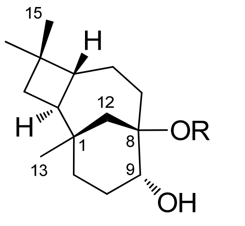

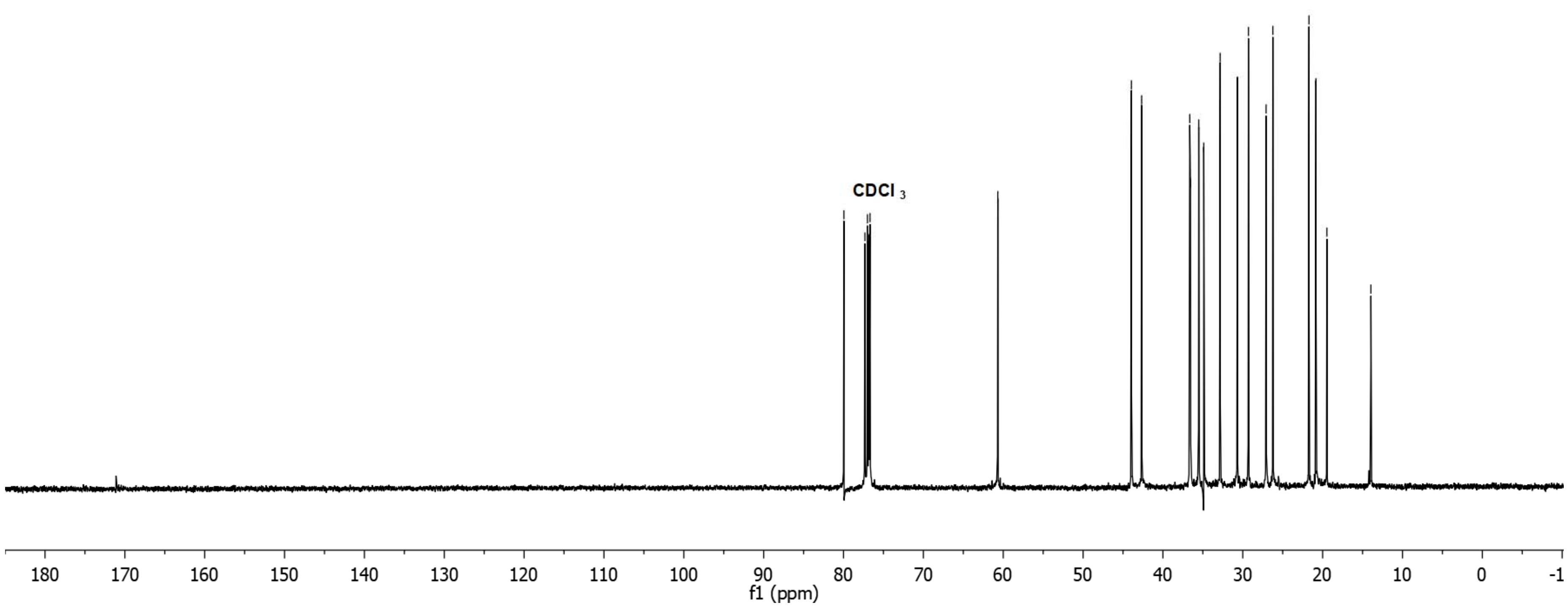

Figure S18. ^{13}C NMR spectrum of compound **4d** in $CDCl_3$ (100 MHz).

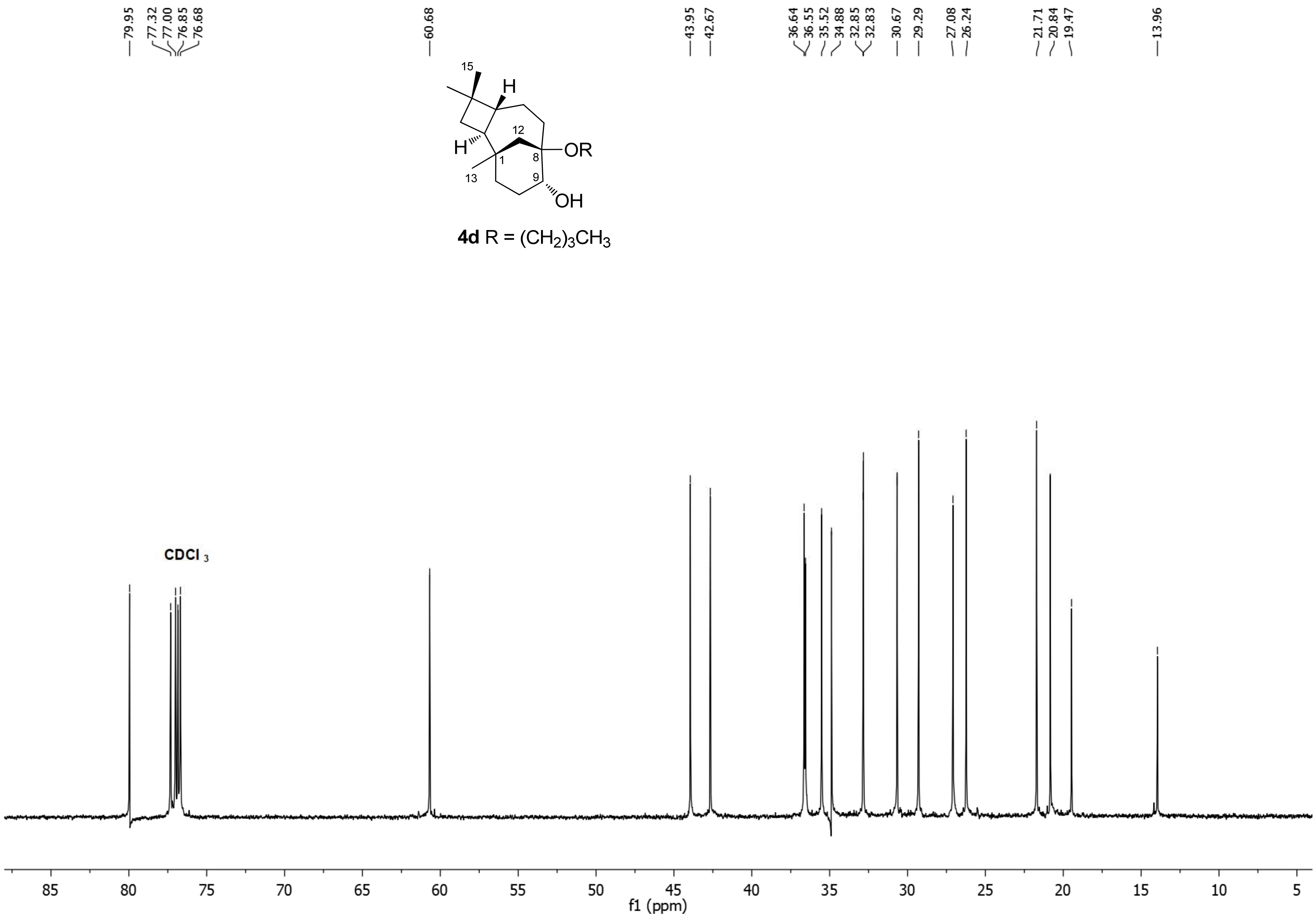

Figure S18a. Expansion (δ_C 85-5) of ^{13}C NMR spectrum of compound **4d** in $CDCl_3$ (100 MHz).

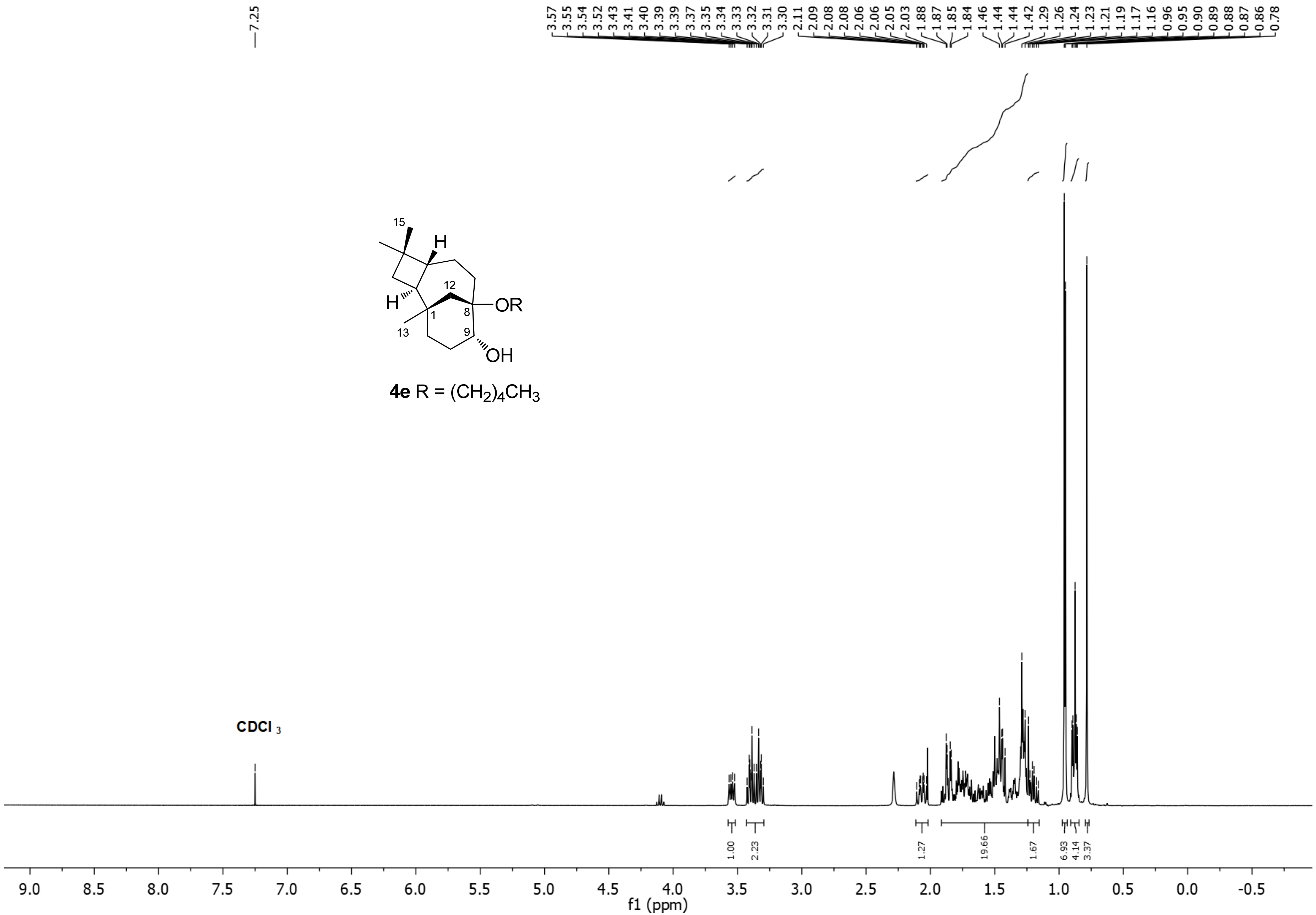

Figure S19. ^{1}H NMR spectrum of compound **4e** in $CDCl_3$ (400 MHz).

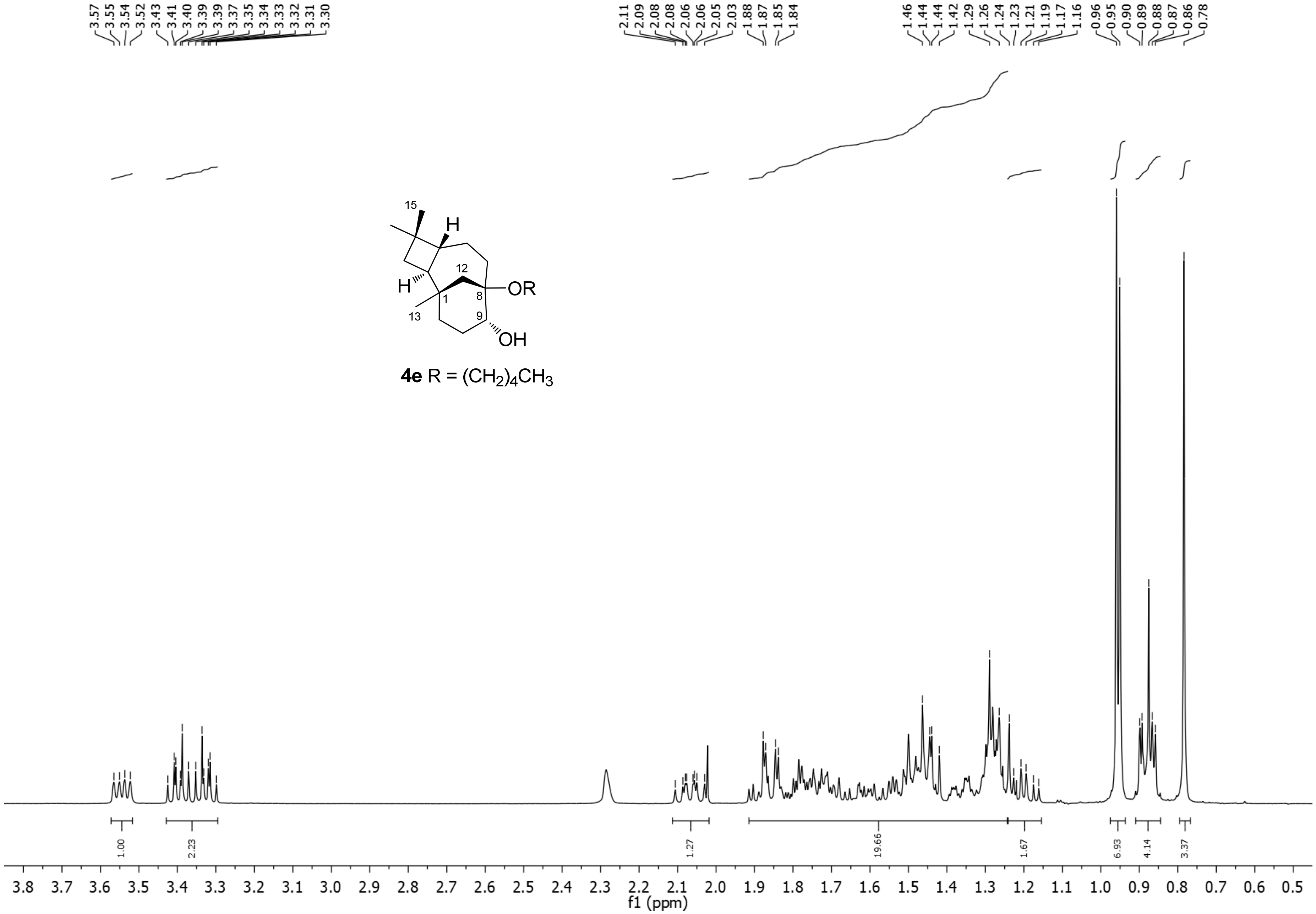

Figure S19a. Expansion (δ_H 3.8-0.5) of ^{1}H NMR spectrum of compound **4e** in $CDCl_3$ (400 MHz).

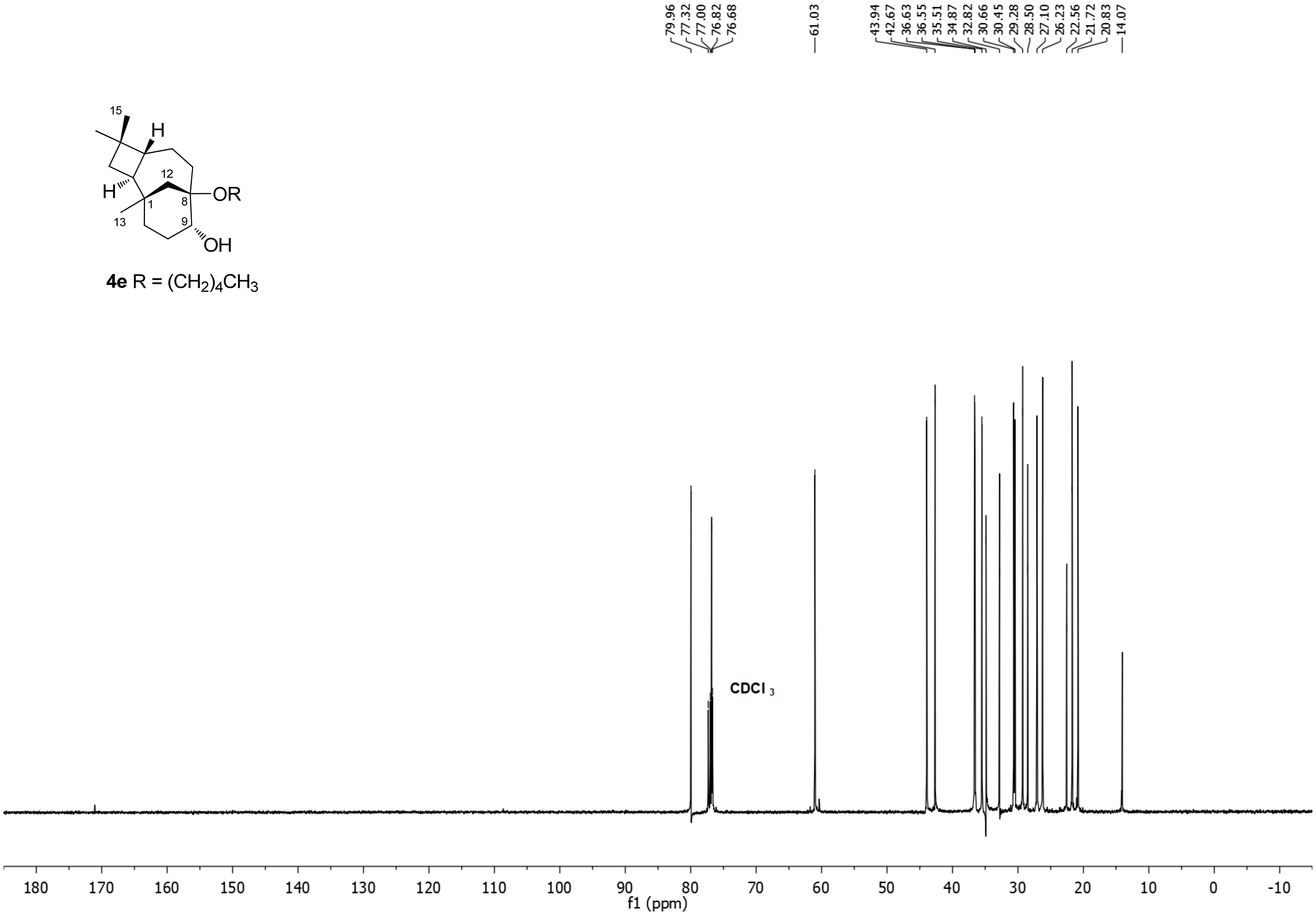

Figure S20. ^{13}C NMR spectrum of compound **4e** in $CDCl_3$ (100 MHz).

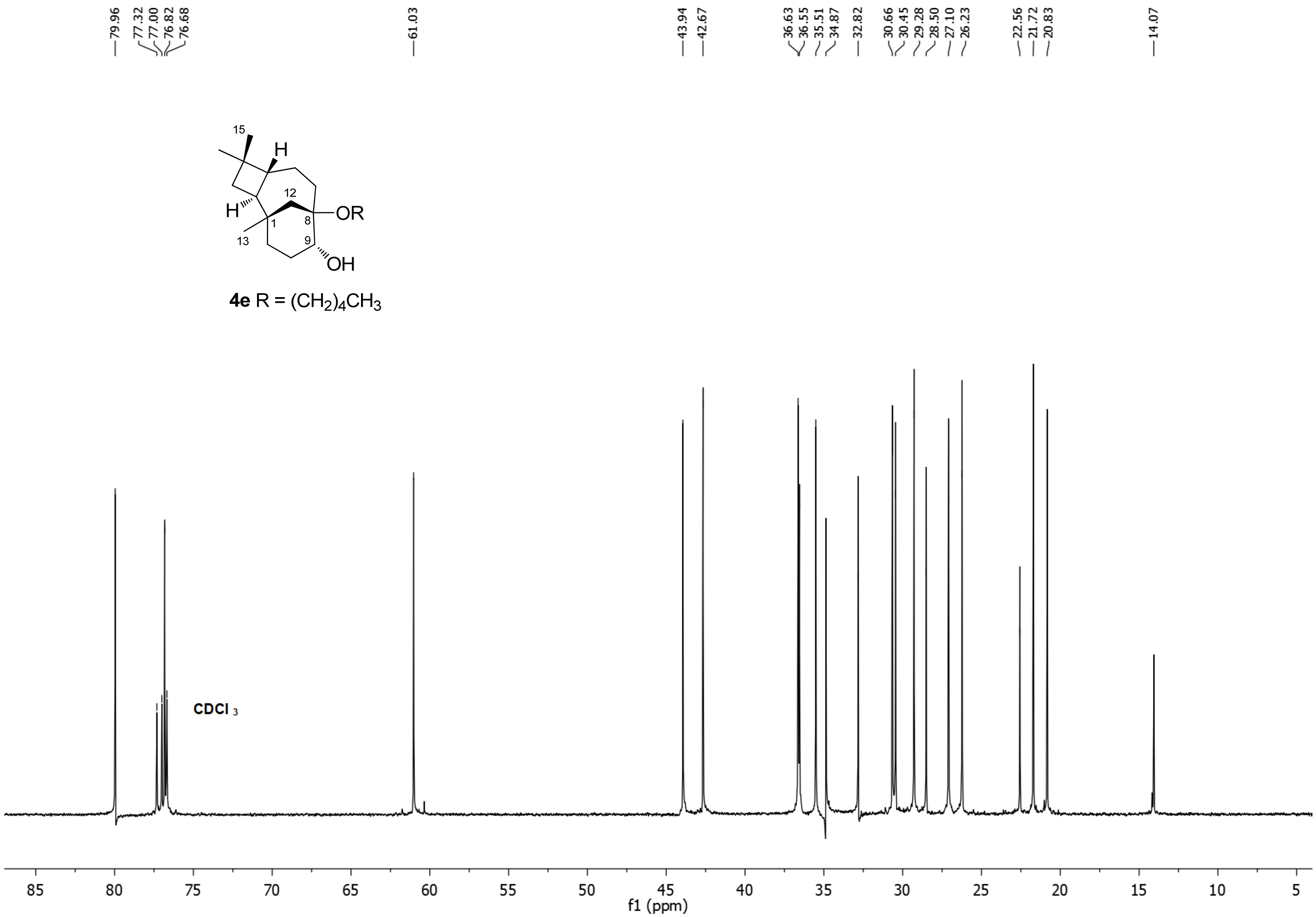

Figure S20a. Expansion (δ_C 85-5) of ^{13}C NMR spectrum of compound **4e** in $CDCl_3$ (100 MHz).
